# Rising tree mortality in France is associated with distinct seasonal climate anomalies

**DOI:** 10.1038/s41467-026-74613-9

**Published:** 2026-06-26

**Authors:** Pascal Schneider, Agnès Pellissier-Tanon, Chuanlong Zhou, Philippe Ciais, Christian Piedallu, Alba Viana-Soto, J. Jelle Lever, Arthur Gessler

**Affiliations:** 1 Forest Dynamics, Swiss Federal Institute for Forest, Snow and Landscape Research (WSL), Birmensdorf, Switzerland; 2 Institute of Terrestrial Ecosystems, ETH Zurich (Swiss Federal Institute of Technology), Zurich, Switzerland; 3 Laboratoire des Sciences du Climat et de l’Environnement, CEA CNRS UVSQ, Gif-sur-Yvette, France; 4 Silva, AgroParisTech, INRAE, Université de Lorraine, Nancy, France; 5 Earth Observation for Ecosystem Management, School of Life Sciences, Technical University of Munich, Freising, Germany

**Keywords:** Forest ecology, Forestry, Macroecology

## Abstract

Tree mortality is rising across European forests, yet its dominant drivers remain difficult to disentangle. Analyzing over 500,000 trees from the French National Forest Inventory (2015-2023), we observe increasing mortality rates across major species. Using ensemble modelling and explainable machine learning, we identify tree size, competition, and seasonal climate anomalies as the primary mortality drivers. Warmer winters and springs are the most frequent and influential climate-related predictors, likely reflecting disturbed phenology and enhanced pest survival during milder cold seasons. Warmer summers are less frequently selected but similarly influential, possibly related to increased pest activity and drought stress. We further identify three drought-related anomaly patterns, each consistent with a distinct mortality pathway: long, intense droughts likely induce acute hydraulic failure; on-average drier summers might drive gradual carbon starvation; and insufficient post-drought rainfall possibly constrains recovery. Anomalously wet springs also frequently emerge as strong predictors of elevated mortality risk, particularly in tall temperate tree species. We hypothesize that this reflects the influence of structural overshoot, whereby wet spring conditions promote rapid canopy growth that may increase vulnerability to subsequent drought. Together, our findings indicate that tree mortality is shaped by interacting and potentially compounding seasonal climate anomalies rather than a single extreme event.

## Abstract (web summary)

This study explores climate-driven tree mortality risk beyond summer drought. It links rising tree mortality in France to distinct seasonal climate anomalies, including winter and spring warming, different drought types, and even wet springs.

## Introduction

Europe’s forests are fundamental to climate regulation, biodiversity conservation, and the provision of essential ecosystem services that sustain both society and the economy^1^. However, climate change is increasingly disrupting these functions through rising temperatures, altered precipitation patterns, more frequent and more severe extreme weather events, and disturbances like wildfires and insect attacks^2–6^. These stressors not only reduce forest productivity^7,8^ but also increase tree mortality risk^9–11^. In addition to acute extreme events, there is mounting evidence that background mortality, the gradual tree death in the absence of immediate disturbances, is rising due to persistent temperature and water stress^12–17^.

Despite growing awareness of climate-driven tree dieback caused by both extreme events and persistent shifts, a more detailed assessment is needed to identify which seasonal climatic changes are most critical and how they interact with species-specific traits and forest dynamics to drive mortality. Seasonal dynamics are particularly important, as each season presents distinct risks and challenges. In winter, warmer temperatures can promote pest survival^5^ and, when combined with reduced precipitation, may limit water recharge, thereby predisposing trees to early-season water stress^18^. In spring, warmer conditions can trigger earlier bud break, increasing vulnerability to late-spring frosts^19^ and the likelihood of experiencing drought^20^, while precipitation deficits can constrain leaf development and early growth^18^. In summer, low rainfall can reduce photosynthesis^7,8^ and cause drought-induced hydraulic failure^11,18,21^, while warmer conditions can intensify drought impacts through hotter droughts^3^ and exacerbate pest outbreaks^22,23^. In fall, warmer temperatures may prolong the growing season^20^, but interfere with dormancy and spring phenology^24^. Meanwhile, reduced fall precipitation may impair carbohydrate storage^18^ and limit post-drought recovery, thus increasing the risk of stress carryover into the next year^9,25^. The type of stress further shapes these seasonal risks^21,26^. For example, when spring and summer become progressively warmer and drier, they limit photosynthesis and weaken trees through carbon starvation, heightening their susceptibility to disturbances like pest outbreaks or droughts^6,23^. In contrast, a short and intense summer drought can cause mortality directly through desiccation and hydraulic failure^21^. Importantly, depending on their genetic makeup, life history, and local forest conditions, trees respond differently to each of these distinct seasonal stressors^26–28^. Thus, understanding forest mortality requires not only identifying the most critical seasonal climate anomalies and stress types, but also understanding how they interact with species traits and vary across ecological contexts.

Although different seasons and stress types expose trees to distinct risks, large-scale mortality studies often simplify these seasonal dynamics by relying on annually aggregated climate variables (e.g., mean annual temperature) to predict tree mortality across species^10,29^. Even when seasonal anomalies are considered (e.g., mean summer temperature), researchers often select a single variable based on statistical model performance^14–17,30,31^, which can obscure seasonal interactions and may contribute to contradictory results on tree mortality across Europe. For example, one study^15^ found higher mortality rates in beech and oak under drier conditions, whereas another^32^ reported higher mortality rates under wetter conditions for the same species. Other studies have shown that the effect of precipitation on mortality varies depending on species and ecological context^15,33,34^. Such inconsistencies may stem from mortality being more sensitive to increasing climate variability than to shifts in annual means^35^, from regionally varying climate sensitivities within species^17,33^, or from structural differences in datasets that capture different ecological contexts. This latter explanation is supported by recent evidence showing that using different subsets of the same tree mortality dataset can substantially alter model outcomes and their interpretation^36,37^. Motivated by these observations, we aim to resolve inconsistencies in mortality patterns, following an approach that captures seasonal climate variability and confronts the challenges posed by heterogeneous datasets.

To predict mortality, we use random forest models, a flexible yet underutilized approach well suited to modeling mortality under complex environmental conditions^37,38^. Compared to classic methods like logistic regression, random forests can better capture nonlinear responses and are generally more robust against outliers and correlated variables^37,39^. Although they have shown promising results in predicting tree mortality in a few studies from the United States^40,41^ and South Korea^42^, they have not been applied to European forests. In this study, we leverage the potential of random forests using data from the French National Forest Inventory (NFI)—a comprehensive dataset covering over 500,000 trees across more than 50 species surveyed from 2015 to 2023, with semi-permanent plots that are revisited after 5 years. The NFI encompasses a range of forested climatic regions representative of the climatic conditions found throughout Europe, including temperate oceanic, subcontinental, alpine, and Mediterranean zones. The objective of this study is to answer the following research questions: (RQ1) How has tree mortality in France changed over the past decade? (RQ2) Which biotic and abiotic factors explain tree mortality best? (RQ3) How did seasonal climate anomalies affect tree mortality across and within species, and which known mortality mechanisms can be attributed to these changes?

**Fig. 1:**
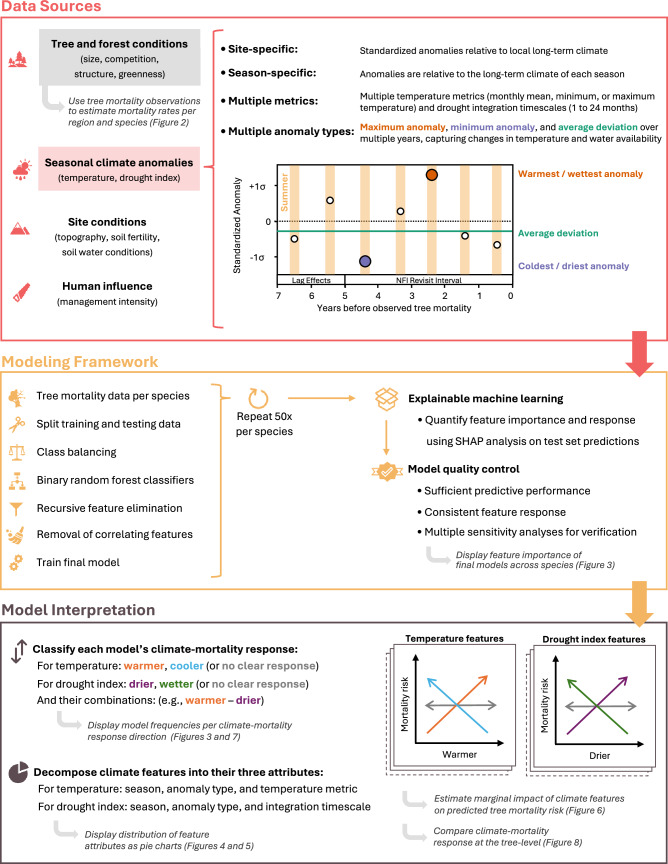
Graphical summary of the methodological framework used to estimate observed tree mortality rates and model tree mortality risk. The upper panel shows the different data streams entering the analysis and highlights seasonal climate anomalies as the central explanatory component. Climate anomalies refer to temperature and drought index anomalies that are standardized relative to site-specific long-term climate and for each season separately. The small figure within the box illustrates the definition of different anomaly types for the temperature or drought index anomalies during summer in the years preceding observed tree mortality. The middle panel summarizes the species-specific recursive modeling framework, in which repeated binary random forest models are used to isolate robust mortality signals from heterogeneous inventory data. The lower panel illustrates the interpretation of model outputs. First, we assessed the climate-mortality response of each temperature and drought index feature by testing whether warmer or cooler, and drier or wetter conditions, respectively, increased predicted mortality risk (see the two inset figures). Then, temperature and drought index features were decomposed into their defining attributes to analyze the most common and influential seasonal climate anomalies. Grey arrows and italic annotations indicate how individual elements of the analysis are displayed in the figures of this study.

To answer these questions, we build species-specific random forest classification models to predict the probability of a tree dying between two NFI visits (i.e., a tree’s mortality risk). We assemble more than 180 candidate features spanning key mortality driver categories, including tree and stand characteristics, seasonal climate anomalies, edaphic and topographic variables, and management intensity. Using recursive feature selection, we then identify the single most influential feature in each category to obtain parsimonious, interpretable models. To assess feature importance, we use a hold-out dataset and Shapley Additive Explanations (SHAP), a game-theoretic approach that quantifies the influence of each feature on a tree’s predicted mortality risk^43^. Since previous studies have shown that results are sensitive to data heterogeneity^36,37^, we repeat the entire modeling process 50 times per species with different random initializations, allowing us to identify robust emerging mortality response patterns across species. Figure 1 provides a visual guide to the analysis, linking the main result figures to the data sources and modeling components from which they originate.

## Results

### Rising tree mortality across France over the past decade

**Fig. 2:**
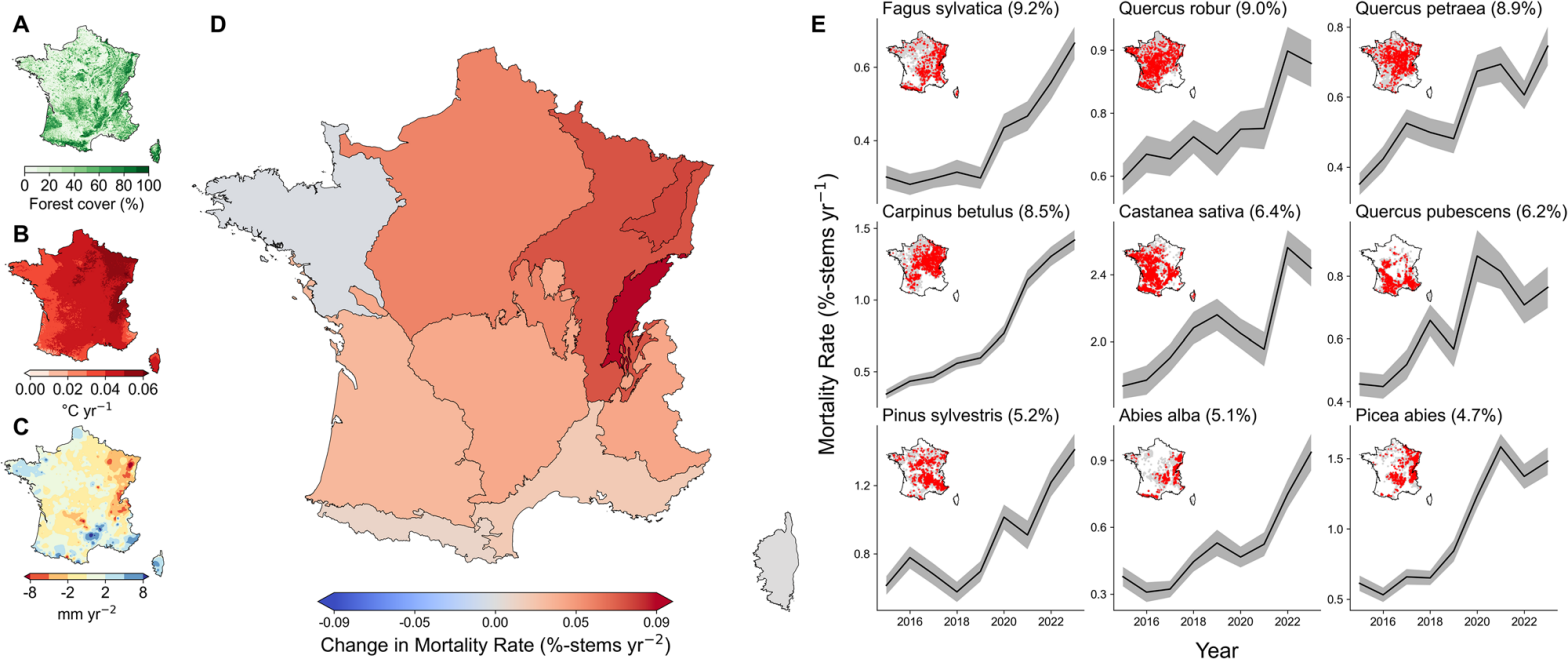
Tree mortality and climate trends across France. **A** Forest cover across France. **B** Change in mean annual temperature from 1980 to 2020. **C** Change in total annual precipitation from 1980 to 2020. **D** Change in tree mortality rates from 2015 to 2023 across France’s greater ecoregions, averaged across the nine most common species by number of trees in the dataset. The change in mortality rate per greater ecoregion was calculated by estimating species-weighted mean annual mortality rates through bootstrapping and then extracting the slopes from linear regressions of annual tree mortality rates against time (see Fig. S2 for regional trends based on all species analyzed in this study). **E** Annual tree mortality rates for the nine most common species, arranged in order of decreasing number of trees per species from top left to bottom right (see Fig. S1 for all species). The percentage in parentheses after each species’ name represents the proportion of trees for that species relative to the total number of trees in the dataset analyzed in this study. The black line represents the bootstrapped mean, with the shaded band indicating ±1 standard deviation (note that the y-axis scale differs across species). The inset maps show each species’ distribution, with grey areas representing sampled trees and red areas indicating where tree deaths occurred between censuses.

Analysis of the French NFI revealed a marked rise in tree mortality between 2015 and 2023 (Fig. 2). Among the nine most common species—*Fagus sylvatica* (European beech), *Quercus robur* (pedunculate oak), *Quercus petraea* (sessile oak), *Carpinus betulus* (European hornbeam), *Castanea sativa* (sweet chestnut), *Quercus pubescens* (downy oak), *Pinus sylvestris* (Scots pine), *Abies alba* (silver fir), and *Picea abies* (Norway spruce) - mortality increased 1.5- to 4-fold (*p* < 0.01, Supplementary Data 1), with hotspots concentrated in northeastern France, especially in Jura, Vosges, and Grand Est (Fig. 2). These regions also experienced sustained warming and annual precipitation declines since 1980 (Fig. 2), suggesting a climatic imprint on mortality patterns. Across all 52 species analyzed here, nearly half (48%) showed significant increases (*p* < 0.01, Supplementary Data 1 and Fig. S1), with spatial distributions broadly consistent across common and less abundant species (Fig. S2).

These observed mortality rates are based on the NFI’s 5-year revisit interval and are conservative estimates because trees that were harvested between visits were treated as survivors (see “Methods”). If harvests largely reflected salvage logging, actual mortality rates could therefore be substantially higher (Fig. S3). Consistent increases also appeared when trees were separated by size, with abundant species showing rising mortality across all diameter classes (Fig. S4). Basal area trends reinforced this pattern: losses from mortality increased steadily while growth gains declined, resulting in net reductions for several dominant species, including *Fagus sylvatica*, *Carpinus betulus*, *Abies alba*, and *Picea abies* (Fig. S5). Harvest-related losses remained stable or decreased, except in *Picea abies*, where they rose sharply, likely reflecting bark beetle-driven salvage logging^44,45^. Taken together, these results suggest that elevated mortality is not offset by increased growth or recruitment, indicating a broader decline in forest health rather than a shift in forest demography.

### Tree size, competition, and climate anomalies drive tree mortality

We compiled 183 candidate features across eleven categories that represent potential mortality drivers, including tree and forest properties, standardized anomalies in temperature and climatic water balance (CWB), site conditions, and management (see “Methods” and Supplementary Data 2). Random forest classification models predicted whether a tree would die during the NFI’s 5-year revisit interval. Following a model-ensemble approach, we fitted for each species 50 model replicates to capture inherent randomness in data structure and model fitting^37^ (i.e., accounting for variation in train-test splits, class balancing, and random forest fitting). For parsimony and interpretability, each model replicate consisted only of eleven non-correlated features, one selected from each driver category through recursive feature selection (Fig. S6 and Supplementary Data 3). Across the 52 species we analyzed, our models showed good predictive power with a mean area under the receiver operating characteristic curve (ROC-AUC) of 0.73 ± 0.05, and within species, replicates varied on average by only 0.04 (Supplementary Data 4 and see “Methods” for additional metrics). This modeling approach thus yielded equifinal models that consistently captured mortality patterns despite differences in feature combinations.

**Fig. 3:**
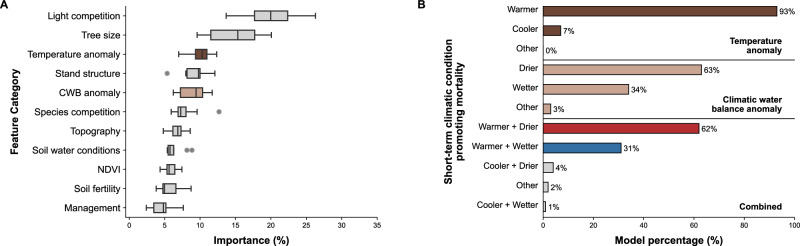
Feature category importance and the short-term climate conditions promoting tree mortality. **A** Boxplots illustrate the importance of each feature category in predicting a tree’s probability of dying between censuses using random forest models. “Importance” refers to the average importance of all features within the same feature category, calculated across all models for a given species (see “Methods”). Boxplots show the median, 25th and 75th percentiles with whiskers extending to the minimum and maximum values within 1.5 times the interquartile range from the quartiles, and dots represent species outliers. The categories “temperature anomaly” and “CWB anomaly” are dark and light brown, respectively, matching the colors in (**B**) (CWB climatic water balance, NDVI normalized difference vegetation index). **B** Bar charts show the percentage of models associating mortality with different temperature anomalies, CWB anomalies, or both combined. “Other” denotes models where no significant association was found between a feature and mortality risk or where the same feature did not show a consistent response direction across models (see “Methods”). The term “short-term conditions” refers to standardized climate anomalies during the years preceding the second tree census. Results for the nine most common species are shown (*N* = 442 models; see Fig. S7 for results across all 52 analyzed species).

We interpreted feature importance and behavior using SHAP values, a game-theoretic approach to analyze the output of machine learning models by quantifying each feature’s marginal contribution to predictions (see “Methods”). Feature importance was summarized per category by averaging results across all model replicates per species and grouping features within the same category. For the nine most common species, tree size and forest properties (light competition, species competition, stand structure) explained 52% of total importance (Fig. 3A). Short-term climate anomalies ranked second (19%). We defined these climate anomalies as site- and season-specific standardized anomalies of temperature and CWB, calculated relative to the 1960–2020 local baseline. They thus express in standard deviations how much warmer or cooler, and drier or wetter, conditions were in a given season at a given location. To account for uncertain mortality timing within the NFI’s 5-year revisit interval and for potential lag effects, these anomalies were summarized over the 7 years preceding a tree’s mortality assessment (see “Methods” and Fig. 1).

Focusing on the nine most common species, the temperature feature in almost all models (93%) associated higher mortality risk with warmer anomalies during these 7 years (Fig. 3B). In 63% of models, the CWB feature associated higher mortality risk with drier anomalies, and in 34% of models with wetter anomalies. When combining the temperature and CWB feature responses within each model, 62% of models associated higher mortality risk with warmer-drier conditions and 31% with warmer-wetter conditions. Such a strong association with warmer-drier conditions was expected, as these conditions are known to increase tree mortality^3^, whereas the warmer-wetter response was surprising and suggests that even seemingly favorable anomalies may elevate mortality risk^21^.

Other feature categories (topography, soil water capacity and fertility, normalized difference vegetation index (NDVI), and management) were generally less important, each contributing under 10%, which matches the results of previous tree mortality studies^12,15^. Expanding this analysis to all 52 species, the results remained similar: tree and forest properties (42%) and climate anomalies (23%) dominated in importance, with 51% of models associating higher mortality risk with warmer-drier anomalies and 22% with warmer-wetter anomalies (Fig. S7).

### Distinct seasonal anomalies shape mortality risk

Because the majority of models (93%; Fig. 3B) associated increased tree mortality risk with warmer temperature anomalies, we focus here on the characteristics of these models. To characterize the seasonal climate signals underlying mortality risk, we decomposed the temperature and CWB features in each model into their three atomic attributes and calculated their frequency across models (see “Methods”). For temperature features, these attributes were (i) the season when the anomaly occurred; (ii) the anomaly type, which defines whether the feature described the maximum, minimum, or average anomaly over the 7 years before a tree’s mortality assessment (representing the warmest, coldest, or average deviation, respectively; Fig. 1); and (iii) the temperature metric describing the monthly maximum, minimum, or mean temperature. For CWB features, the three attributes were (i) the season when the anomaly occurred; (ii) the anomaly type describing the maximum, minimum, or mean water availability deviation (i.e., the wettest, driest, or average deviation); and (iii) the integration timescale indicating the number of months over which the CWB was aggregated.

**Fig. 4:**
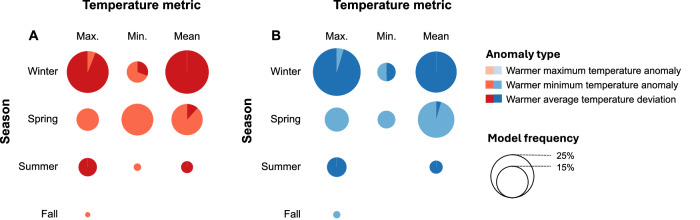
Tree mortality risk is associated with on-average warmer winter and summer conditions and with warmer spring minimum temperatures. The figure shows the distribution of temperature anomaly attributes from models associating increased mortality risk with warmer temperature anomalies. Models are split by whether the concurrent CWB feature associated mortality with drier (**A** red shades, *N* = 274 models) or wetter (**B** blue shades, *N* = 137 models) anomalies of the climatic water balance. To derive these distributions, the selected temperature feature in each model was decomposed into its three attributes: season, temperature metric, and anomaly type (see “Methods”). The “season” (rows) corresponds to the months over which anomalies were calculated (e.g., winter refers to December–February), while the “temperature metric” (columns) denotes whether anomalies were based on monthly maximum, minimum, or mean temperature. The “anomaly type” (color shades) denotes whether a temperature feature quantified the maximum anomaly, minimum anomaly, or the average deviation during the 7 years preceding the second tree census. The “model frequency” (pie chart size) reflects the percentage of models within each panel that belong to a given season-temperature metric combination. Pie slices indicate the distribution of different anomaly types within each combination. Results are shown for the nine most common species (see Fig. S10 for all species). Example interpretation: the pie chart in the upper left of (**A**) indicates that approx. 25% of all warmer-drier models identified winter (first row) monthly maximum temperature (first column) as the main mortality-associated temperature anomaly. Among these models, most features represented warmer average temperature deviations (red slice), while a small fraction represented warmer minimum temperature anomalies (orange slice).

For temperature, mortality risk was most frequently linked to warmer winters and springs, and less frequently to warmer summers (Fig. 4). Specifically, 42% of models associated higher mortality risk with on-average warmer winter temperatures, primarily based on monthly maximum or mean temperature (first row in Fig. 4, across both subpanels). Another 35% of models linked mortality risk to warmer spring minimum temperature anomalies, mostly based on monthly minimum or mean temperature (second row in Fig. 4). A smaller share of models (8%) associated mortality risk with on-average warmer summer temperatures, mainly based on the monthly maximum or mean temperature (third row in Fig. 4). The distribution of these temperature anomaly attributes remained similar irrespective of whether the concurrent CWB feature in a model linked mortality to drier or wetter conditions, indicating that the seasonal structure of warming-related mortality risk was largely independent of moisture context (compare Fig. 4A, B).

**Fig. 5:**
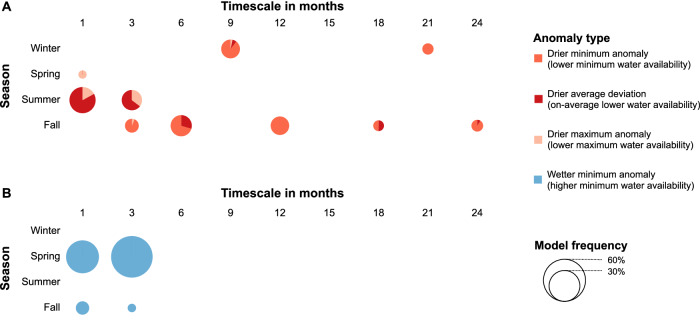
Tree mortality risk is associated with three distinct types of droughts and with anomalously wet spring conditions. The figure shows the distribution of the attributes of climatic water balance (CWB) features from models associating increased mortality risk with either drier (**A** red shades, *N* = 274 models) or wetter (**B** blue shades, *N* = 137 models) anomalies. Only models in which warmer temperature anomalies concurrently increased mortality risk are shown; reported model frequencies therefore refer to the subsets of warmer-drier and warmer-wetter models, respectively. To derive these distributions, the CWB feature in each model was decomposed into its three attributes: season, integration timescale, and anomaly type (see “Methods”). The “season” (rows) refers to the final month of the season (e.g., February for winter), while the “timescale” (columns) denotes the number of months over which the CWB was integrated prior to that month (e.g., “fall, 6” corresponds to June–November). The “anomaly type” (color shades) denotes whether a CWB feature quantified the maximum anomaly, minimum anomaly, or average deviation during the 7 years preceding the second tree census. The “model frequency” (pie chart size) reflects the percentage of models within each panel that belong to a given season-timescale combination. Pie slices indicate the distribution of different anomaly types within each combination. Results are shown for the nine most common species (see Fig. S11 for all species). Example interpretation: the largest pie chart in (**B**) shows that among models linking mortality to warmer-wetter conditions, approx. 60% associated increased risk with wetter minimum anomalies, i.e., higher minimum water availability (March–May, 3-month integration timescale).

For models linking mortality to drier conditions, three distinct drought-related patterns emerged from the CWB attributes (Fig. 5A). First, 46% of models associated mortality with reduced minimum water availability integrated over more than 6 months, typically bottoming in fall or winter, reflecting prolonged multi-month droughts. Second, 28% linked mortality to on-average drier summer conditions, reflecting chronic summer water stress. Third, 22% associated mortality with reduced maximum water availability in summer, reflecting reduced summer water surplus. In contrast, among models linking mortality to wetter conditions, almost all (86%) identified higher minimum water availability in spring (Fig. 5B), suggesting that reduced spring water deficits (or the absence of water stress) increased mortality risk.

**Fig. 6:**
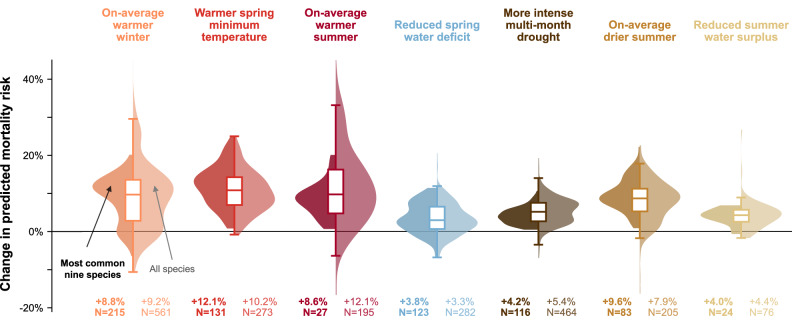
Local marginal effects of dominant seasonal climate anomalies on predicted tree mortality risk. Violin plots show the change in predicted mortality risk when a single temperature or water-balance feature is increased by one standard deviation, while all other predictors in the model remain constant (see “Methods”). The perturbed features correspond to the main temperature and CWB anomalies identified in Figs. 4 and 5. For each violin plot, the darker (left) distribution summarizes responses across the nine most common species, and the lighter (right) distribution summarizes responses across all 52 species. Values below each distribution indicate the mean change in predicted mortality risk and the number of contributing models (*N*); in bold for the nine most common species and in normal font for all 52 species. Boxplots show the median, 25th and 75th percentiles for model outputs across all 52 species; whiskers extend to the minimum and maximum values within 1.5 times the interquartile range from the quartiles.

To quantify and compare the effect of these frequently identified seasonal anomalies, we recalculated predictions for each model after increasing its respective temperature or CWB feature by one standard deviation, while keeping all other variables constant, thereby estimating local marginal effects on predicted mortality risk (Fig. 6 and see “Methods”). Warmer winter, spring, and summer temperature anomalies showed the strongest marginal effects, increasing predicted mortality risk on average by 8.6–12.1%. The different drought anomalies increased risk by 4.2–9.6%, whereas wetter spring anomalies increased it by 3.3–3.8%. Although temperature and CWB anomalies typically occurred in different seasons, models in which they coincided showed amplified marginal effects, for example, when a summer was modeled to be both warmer and drier (Fig. S8). Although these estimates align with previous studies using logistic regression models^15^, it should be noted that random forest responses are inherently nonlinear, meaning these estimates reflect local sensitivity rather than effects that scale linearly with anomaly magnitude.

Furthermore, the importance of these different seasonal climate anomalies for predicting mortality risk changed over time (Fig. S9). The importance of on-average warmer winters and summers increased throughout the study period, whereas warmer spring minimum temperatures became important mainly after 2020. The importance of on-average drier summers and intense multi-month drought rose mainly around 2018, with drier summers continuing to increase thereafter. The importance of reduced spring water deficits also showed a pronounced jump in 2018, while reduced summer water surplus became important primarily after 2020. Together, these temporal patterns indicate that mortality risk reflects the changing contribution of multiple seasonal anomalies rather than a single extreme event.

Extending the analysis of seasonal climate anomalies to all 52 species revealed interspecific variation, while the main patterns described above persisted (Figs. S10–S12 and Supplementary Data 5–8). For example, the mortality of *Fagus sylvatica* and *Pinus sylvestris* was often associated with wet spring conditions, whereas *Quercus pubescens* was exclusively associated with intense multi-month droughts. As another example, mortality of *Castanea sativa* was frequently associated with warmer spring minimum temperatures, whereas mortality of *Picea abies* was mainly associated with on-average warmer winters.

### Wetter springs elevate mortality risk in tall, temperate species

**Fig. 7:**
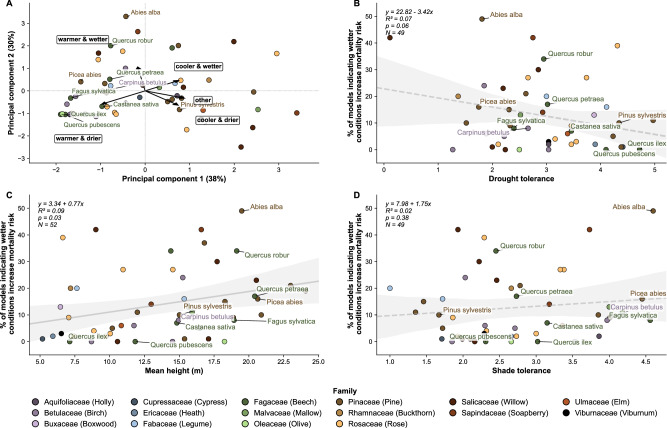
Species-specific climate sensitivity and its links to ecological traits. **A** Principal component analysis (PCA) based on the frequency of models associating increased mortality with different types of short-term climate anomalies. Arrows indicate the loadings of each anomaly category (i.e., each species’ share of models associating mortality with warmer, cooler, wetter, drier, or other conditions), and thus, species are positioned according to their overall climate sensitivity profile. Axis labels include the percentage of variance explained by each principal component. The remaining panels show the relationships between each species’ percentage of models associating mortality with wetter spring conditions and its drought tolerance (**B**), mean height (**C**), and shade tolerance (**D**). Solid grey lines indicate significant linear regressions (*p* < 0.05), while dashed lines indicate nonsignificant trends. Shaded areas represent 95% confidence intervals around the mean fitted linear regression line. Regression equations and *R*^2^ values are shown in the top-right corner of each panel. Species are color-coded by taxonomic family.

We next tested whether species differed in their climate-mortality responses. A principal component analysis grouped species by the percentage of models linking mortality to specific anomalies (Fig. 7A, and see “Methods”). In this climate-response space, temperate oaks (*Quercus robur, Quercus petraea*) showed a higher proportion of models linking increased mortality risk to wetter conditions than Mediterranean oaks (*Quercus pubescens, Quercus ilex*), suggesting that the more drought-tolerant Mediterranean species are less susceptible to anomalously wet conditions. Using a drought-tolerance index^46^ as a proxy for climatic origin (see “Methods”), we found the same pattern: across all models, the percentage of models linking mortality to wetter spring conditions decreased significantly with increasing drought tolerance (Fig. 7B), whereas the percentage of models linking mortality to drier summer conditions increased, although not significantly (*p* = 0.12). Similarly, within the subset of models linking mortality to warmer anomalies, the percentage of models linking mortality to wetter spring conditions decreased with increasing drought tolerance; respectively, the percentage of models linking mortality to drier summer conditions increased (however, both were nonsignificant with a *p* = 0.10).

To explore why wetter springs may increase mortality risk, we tested two trait-based hypotheses from earlier studies. The first one—known as structural overshoot—posits that wet springs stimulate excessive canopy growth and increase vulnerability to subsequent drought-induced hydraulic failure^21,47^, particularly in taller trees^48–50^. The second suggests that canopy growth during wet springs intensifies shading, disadvantaging smaller or shade-intolerant species^32^. Across all models, the percentage of models linking mortality to wetter springs showed a significant linear increase with mean species height (Fig. 7C), whereas no relationship was observed for drier summers (*p* = 0.70). Within models linking mortality to warmer anomalies, the percentage of wetter spring associations also increased with height (*p* = 0.005) but showed no relationship with drier summer associations (*p* = 0.91). Shade tolerance was not significantly related to the percentage linking mortality to wetter springs (Fig. 7D) or to any other condition (warmer-wetter: *p* = 0.07; drier: *p* = 0.93; warmer-drier: *p* = 0.47). These results indicate that taller, temperate species were more frequently associated with mortality under anomalously wet spring conditions, possibly due to structural overshoot. The next section examines whether structural overshoot also emerges from tree-level responses within species.

### Coupled sensitivity to seasonal water mismatch

**Fig. 8:**
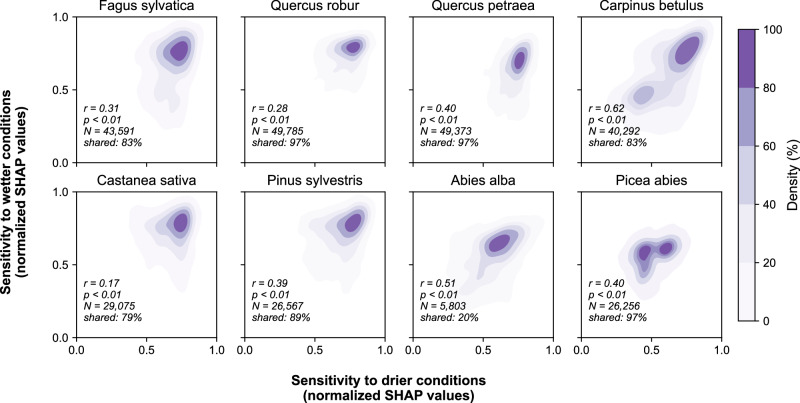
Tree-level sensitivity to wetter spring and drier summer anomalies across the nine most common species. Bivariate density plots showing tree-level sensitivity to wetter spring and drier summer conditions, based on normalized SHAP values of CWB features (see “Methods”). Color intensity indicates the density of trees with similar sensitivity. Correlation coefficients (two-sided Spearman’s *r*) quantify how strongly trees sensitive to wet anomalies were also sensitive to dry anomalies. The “shared” percentage indicates the proportion of trees out of all trees (for a given species) occurring in test sets of both the wetter- and drier-response model subsets. “*N*” indicates the corresponding count of shared trees used to estimate the density and correlation. Panels show the nine most common species; *Quercus pubescens* is excluded because no models linked mortality to wetter conditions (see Fig. S13 for all species).

To test whether structural overshoot also occurs within species, we examined tree-level responses to wetter spring and drier summer anomalies. Each model included only one CWB feature and therefore linked mortality to either anomaly. Across model replicates, however, individual trees appeared in the test sets of both subsets of models, allowing us to compare how the same trees responded under models associating mortality with wetter spring versus drier summer conditions (see “Methods”). The number of trees appearing in the test sets of both wetter spring models and drier summer models was generally high. For example, in *Fagus sylvatica*, most models linked mortality to drier summer conditions (84%) and fewer to wetter spring conditions (16%; see Supplementary Data 7), but 83% of trees appeared in the test set of both subsets of models (Fig. 8). This overlap was lower in some species, for example in *Castanea sativa* (79%) and *Abies alba* (20%), indicating that wetter spring and drier summer mortality signals often involved different subsets of trees (Fig. 8). To compare tree-level responses to wetter spring and drier summer anomalies, we derived sensitivity scores from each model’s SHAP values (see “Methods”). We found that the sensitivity to wetter spring and drier summer anomalies was overall positively correlated. Among the nine most common species, mean Spearman’s *r* was 0.38 ± 0.13 (mean ± sd; Fig. 8), and similar though weaker correlations were observed across most of the 52 species analyzed (*r* = 0.27 ± 0.22; Fig. S13). These results indicate that trees more sensitive to wetter spring conditions also tended to be more sensitive to drier summer conditions, consistent with the structural overshoot hypothesis.

We further tested whether tree height modulated the sensitivity to wetter spring anomalies, because the structural overshoot hypothesis posits that taller trees should be more sensitive to subsequent drought stress due to greater hydraulic demand from an enlarged canopy^21,47–50^. In contrast, the intensified shading hypothesis suggests greater sensitivity in smaller individuals. Among the nine most common species, taller trees of *Carpinus betulus* and *Castanea sativa* were significantly more sensitive to wetter spring conditions; however, *Quercus robur,* for example, showed the opposite response, with smaller trees being more sensitive to wetter spring conditions (Supplementary Data 9). For most of the 52 species, no significant relationship between tree height and sensitivity to wetter spring anomalies was detected, and results were similarly inconclusive for sensitivity to drier summer anomalies (Supplementary Data 9). These results suggest that height-related patterns align with the structural overshoot hypothesis in some species, but do not consistently explain tree-level sensitivity within various species.

## Discussion

### Widespread signals of climate-driven forest decline

Tree mortality has surged across French forests over the past decade, with ecologically and economically important species, such as *Fagus sylvatica*, *Quercus robur*, and *Picea abies,* showing steep increases (RQ1, Fig. 2E). Many species showed a pronounced surge after 2018, highlighting the vulnerability of European forests to extreme drought events^11^, while spatial patterns pointed to the compounding role of long-term regional warming and drying. These trends span size classes and coincide with reduced growth (Figs. S4 and S5), indicating they do not reflect demographic turnover but widespread climate-driven declines in forest health. Similar reports across Europe reinforce that these processes are continent-wide, consistent with evidence that ongoing warming and drying are eroding forest health^10,12,15,31,33,35,51,52^.

Our random forest models identified tree size and forest stand conditions as the strongest predictors of mortality, reflecting background losses from aging and competition^27,53^ (RQ2, Fig. 3A). Climate anomalies ranked second, confirming climate change as a major driver of rising risk. As expected, most models linked mortality to warmer and drier conditions, but unexpectedly, many also pointed to elevated risk under wetter conditions, indicating that even seemingly favorable conditions can be problematic (Fig. 3B). While species-specific responses varied (Fig. S12 and Supplementary Data 5–9), general patterns emerged across species showing that seasonal temperature and CWB anomalies had distinct characteristics and a comparable influence on mortality risk (Figs. 4–6). Below, we discuss the possible pathways through which each of these distinct anomalies may influence tree mortality (RQ3).

### Disentangling the roles of seasonal warming and drought stress

On-average warmer winter temperatures and warmer spring minimum temperatures stood out as particularly frequent and influential drivers of increased mortality risk (Figs. 4 and 6). Although these anomalies occur in different seasons, they point to related physiological and biotic stress pathways. Warmer winters shorten or disrupt dormancy, leading to insufficient chilling, desynchronized spring leaf-out^54^, and reduced investment in frost protection, which increases vulnerability to sudden cold spells^35,55^. Lower dormancy may also require trees to sustain higher metabolic activity through winter, gradually depleting carbon reserves and increasing starvation risk^21^. Warmer spring minimum temperatures can further advance bud break, increasing exposure to late frosts^56^, extending the growing season, and raising the likelihood of encountering a drought during the growing season^20^. Together, winter and spring warming may also amplify biotic pressures, as milder cold-season conditions allow pests to survive, emerge earlier, and reproduce more rapidly^5,23,26^. Consistent with these mechanisms, pest-sensitive species, such as *Picea abies*^5^, *Fraxinus excelsior*^57^, and *Castanea sativa*^58^, showed particularly high increases in the modeled additional mortality risk under warmer winter or warmer spring conditions (Supplementary Data 8). These results underscore that warming during winter dormancy and early spring can substantially elevate mortality risk, long before the onset of more commonly studied summer stress.

On-average warmer summer temperatures were less frequently identified than winter or spring anomalies yet exerted a comparable influence on mortality risk when present (Figs. 4 and 6). Elevated summer temperatures intensify atmospheric water demand, contributing to “hotter droughts,” a well-established driver of tree mortality globally^3,59^ and across Europe^10,11^. The relatively low frequency of summer temperature features across models likely reflects that temperature-driven evaporative demand was already captured by the drought index, such that impacts are expressed through CWB features rather than temperature features. Nevertheless, when warmer summers coincided with drier summer conditions, modeled mortality risk increased even more (Fig. S8), indicating that warmer summers may amplify drought stress, although they may also increase stress through increased respiratory carbon losses (increasing the risk of carbon starvation^21^) or accelerated pest development (e.g., bark beetles produce more generation in warmer summers, compounding stress in already weakened trees and enabling infestation of healthy ones^5^).

Beyond temperature, three distinct groups of drought anomalies emerged (Fig. 5A) that showed similar overall marginal effects on predicted mortality risk (Fig. 6) but differed in their temporal dynamics (Fig. S9), aligning with established drought frameworks that distinguish between the intensity and duration of water stress and their interaction^21,22,26^. On-average drier summers exerted the strongest marginal effects and increased steadily in importance during and after 2018, whereas intense multi-month droughts showed slightly smaller marginal effects and plateaued around 2018. This contrast indicates that, although multi-month droughts were frequently identified around 2018, their per-event impact on predicted mortality was slightly smaller than that of sustained summer drying. These results align with recent climate analyses showing that while the period from 2018 to 2020 was exceptionally dry across much of France^60,61^, the most extreme 2018 summer conditions were spatially concentrated in northern regions^62^. Thus, models linking mortality to intense and long multi-month droughts likely captured the localized impact of the 2018 extreme event, whereas models linking mortality to on-average drier summers captured the broader and more persistent accumulation of carbon depletion and water stress across multiple years. In contrast, reduced summer water surplus had more moderate marginal effects and became important primarily after 2018, which may reflect limited replenishment of depleted carbon and water reserves following the 2018 drought. Together, these patterns indicate that mortality was shaped not by a single extreme drought, but by the interaction of sustained drying, episodic extreme stress, and constrained recovery over subsequent years.

Importantly, species differed in which drought anomaly was most relevant (Fig. S12 and Supplementary Data 6–8). For example, *Fagus sylvatica* is considered relatively drought-tolerant due to its anisohydric behavior and recovery capacity^34,63^, but many individuals failed to recover from the 2018 drought, with mortality continuing to rise in subsequent years^9^. Our models for *Fagus* sylvatica mirrored this observation: only about a third linked higher mortality risk to severe summer drought conditions, whereas most associated it with reduced maximum water availability in summer, likely reflecting a lack of recovery after 2018 (Fig. S9). In contrast, *Pinus sylvestris,* for example, was exclusively linked to intense drought stress peaking in fall (Fig. S12 and Supplementary Data 6), possibly reflecting its dependence on fall recharge^25^. These examples illustrate that drought-related mortality mechanisms differ substantially across species and that no single drought metric sufficiently explains mortality across diverse species. Instead, mortality risk is shaped by species-specific sensitivity to different drought types and the interactions between acute hydraulic failure, chronic carbon starvation, and impaired recovery, ultimately amplifying vulnerability to secondary disturbances, such as pests, fire, or windthrow^21,23,26,64^.

### The paradox of favorable growth conditions

In contrast to drought stress, wetter spring conditions are generally considered favorable because they promote growth at the start of the growing season^65,66^. However, this assumption may not always hold, as more studies observe increased mortality under generally favorable, wet growing conditions^21,34,47,63,67,68^. Consistent with these findings, our models frequently associated wetter spring anomalies with increased tree mortality risk (Fig. 5B), with effects within the range of warmer temperatures and drought-related stress (Fig. 6). Several mechanisms could help explain this result. As discussed above for warmer springs, wetter conditions may also enhance biotic pressures by facilitating pest activity^6^; however, the pest-affected species highlighted above did not show particularly strong sensitivity to wetter spring anomalies in our analysis (Supplementary Data 8). Increased canopy growth due to higher water availability may also intensify light competition in the understory^32^, but this mechanism is also not supported by our results, as neither shade-intolerant species nor smaller species or smaller trees showed systematically higher mortality risk under wetter spring conditions (Fig. 7 and Supplementary Data 9). Finally, larger canopies may increase exposure to windthrow^6^; however, windthrow events were only sparsely recorded in the NFI, and their influence could thus not be evaluated explicitly.

Instead, our results most strongly support the structural overshoot hypothesis as the most plausible mechanism. Structural overshoot occurs when favorable growth conditions drive excessive canopy growth, leaving trees more vulnerable to hydraulic failure during subsequent droughts^21,34,47^. Several findings in our study support this mechanism. At the species level, taller trees were disproportionately more sensitive to wetter springs (Fig. 7C), consistent with empirical observations of higher evaporative demand and hydraulic risk in larger trees^48^. The additional evaporative demand imposed by an enlarged canopy reduces hydraulic safety margins during drought because a higher minimum conductance (i.e., water loss through inefficient stomatal closure or cuticular transpiration) pushes xylem water potentials closer to hydraulic failure thresholds^69^. We further found that trees sensitive to wetter spring anomalies also tended to be sensitive to drier summer anomalies (Fig. 8), indicating a shared mortality pathway shaped by seasonally mismatching water availability. In addition, wetter spring anomalies became most influential for predicting mortality risk in 2018 (Fig. S9), consistent with the notion of an interaction between favorable spring growth and subsequent summer stress: a modeling study showed that the unusually wet spring of 2018 promoted canopy expansion, which accelerated soil moisture depletion during the subsequent summer drought, and thus, exacerbated declines in photosynthetic productivity^70^. Our findings, therefore, suggest that this unique temporal mismatch between water abundance in spring and scarcity in summer in 2018, a combination not seen for recent droughts in France (Fig. S14), could have been an important mortality trigger.

We also found that the risk of structural overshoot varies with a species’ genetic origin, with temperate species having mortality more often associated with wetter springs than drought-tolerant Mediterranean species (Fig. 7A, B). This interpretation aligns with previous NDVI analyses showing that structural overshoot (quantified as anomalously high NDVI during wet springs followed by stronger NDVI declines in subsequent summer droughts) is particularly common in temperate and boreal regions^71^, where short growing seasons force rapid canopy expansion to maximize photosynthesis, possibly at the expense of root growth^72^. *Fagus sylvatica* reflects this pattern well: it dominates European forests by aggressively expanding its canopy and outcompeting neighbors for light^34^. Yet this competitive advantage may carry the trade-off of structural overshoot when favorable growth conditions are followed by hot and dry summers, which are becoming more frequent and severe in Europe^60,61^. Additionally, because of its limited plasticity in root-to-canopy ratio, *Fagus sylvatica* may not easily acclimate to drier conditions by extending its root area to compensate for its overshoot in time^34^. In fact, empirical evidence shows that compensating root growth often lags opportunistic canopy growth. For example, irrigation experiments on mature *Pinus sylvestris* (which was also strongly affected by wetter spring anomalies; Fig. S12) showed rapid increases in stem and canopy growth within the first years, whereas increases in root biomass lagged by up to a decade^73^. Thus, the interplay between evolutionary growth strategies and shifting environmental conditions could explain why we and others^32,34,47^ observed increased mortality risk for temperate tree species growing under otherwise favorable, wet conditions. By contrast, Mediterranean species are adapted to recurrent water scarcity and, especially evergreens, may benefit from longer growing seasons and may rely less on rapid canopy expansion in spring^71^. Thus, excess carbon gained during favorable growth periods may be more often allocated to roots, xylem, and reserves, to maintain high drought resistance^23^. Consistent with this explanation, we found that Mediterranean species were less often affected by wetter spring anomalies (Fig. 7A, B). Nonetheless, studies have reported structural overshoot in Mediterranean species^67,74,75^, suggesting that it can promote mortality risk beyond temperate regions. Interestingly, wetter springs in our models were characterized by reduced spring water deficits relative to the site norm. This suggests that when spring water scarcity is less severe than what is usually to be expected at a given site, it may fail to provide a certain growth constraint, encouraging trees to over-invest in canopy growth. While this interpretation remains tentative, it supports the notion that relative deviations from local climatic expectations, rather than absolute water availability, shape tree mortality risk^21,68^.

### Limitations and future directions

While our findings were robust across multiple sensitivity analyses (see “Methods”), several limitations should be noted. Most importantly, the 5-year revisit interval of the NFI precludes precise dating of individual tree death, limiting our ability to causally attribute mortality to specific seasonal anomalies. As a result, we cannot unambiguously distinguish between anomalies acting concurrently (e.g., warmer and wetter springs jointly elevating mortality risk; Fig. S8) and those acting sequentially (e.g., wet spring conditions preceding the 2018 summer drought; Fig. S8). Future studies based on annual monitoring, dendrochronological records, or higher-frequency physiological measurements are needed to more precisely quantify and temporally align the effects of such compounding seasonal anomalies. This need is particularly pressing given that mismatches between excess water availability in spring and extreme summer drought may occur more frequently in Europe under continued climate change^61,76^. Similarly, while the specific seasonal anomalies identified here for different species appear ecologically plausible, they should be interpreted as testable hypotheses that require independent confirmation. In addition, our models cannot disentangle direct climatic effects from those mediated through biotic agents, such as pests or pathogens. These agents are known to interact strongly with warming and drought stress^5,6,21,23^ but were not systematically recorded in the NFI or available complementary data sources. Seasonal climate anomalies may therefore partly capture these pressures indirectly (e.g., warmer winters favoring pest survival^6^), but this link remains inferential. Finally, although species-level trait analyses supported the structural overshoot hypothesis, corresponding tree-level relationships between height and mortality risk under wetter spring conditions were not consistently detected. This likely reflects increased variability and noise at the individual-tree level, where heterogeneity in local conditions and growth history can obscure species-level patterns. Addressing this gap will require data that directly link canopy development, hydraulic function, and carbon allocation, for example, through tree-ring analyses combined with physiological measurements^67^ or controlled experiments with manipulated spring water availability and subsequent drought exposure.

In conclusion, tree mortality has risen sharply across France over the past decade, underscoring the urgency of understanding the mechanisms behind forest decline. Using interpretable machine learning with NFI data, we show that mortality risk is strongly shaped by seasonal climate variability. Species differ in which anomalies matter most, yet a consistent picture emerges: mortality risk is shaped by combinations of distinct seasonal climate anomalies rather than by single extreme events or changes in annual baseline conditions. Surprisingly, even favorable spring conditions may increase risk, most plausibly through higher pest pressure or structural overshoot that increases drought vulnerability, though these hypotheses require further investigation. Our findings highlight compounding seasonal stresses as central to forest vulnerability. Management must therefore prepare for both drought and the delayed, sometimes paradoxical, impacts of seemingly favorable conditions. For example, interventions such as targeted thinning, structural diversification, and the promotion of drought-resilient species, will be critical to buffer forests against increasing seasonal climate variability in a changing climate^77^.

## Methods

### Estimation of tree mortality across France

The French NFI^78^, coordinated by the Institut National de L’Information Géographique et Forestière (IGN), uses a quasi-systematic sampling method across France’s climatically diverse forested areas. Each year, a representative sample of the entire country is surveyed. The sampling design relies on a 1 km^2^ grid that divides the region into ten annual sections (see Fig. S15). These are split into two 5-year groups: for example, plots first visited in the first 5-year cycle are placed next to plots first visited in the second cycle, facilitating logistical pairing of initial visits with revisits of the first cycle plots after 5 years. After revisiting, plots are retired, and sampling resumes at nearby locations, maintaining spatial coverage without the need for fixed, permanent plots. To confirm forest presence and accessibility, all potential plots are initially checked using orthophotos and land-use data (plots in non-forested or hard-to-access areas are left out of field sampling).

Each year, approx. 7000 new plots are established, and the approx. 7000 plots from the preceding 5-year cycle are revisited. Each valid plot includes three nested circular subplots, with trees recorded in three diameter-at-breast-height (DBH) size classes: 6 m radius (7.5 ≤ DBH < 22.5 cm), 9 m (22.5 ≤ DBH < 37.5 cm), and 15 m (DBH ≥ 37.5 cm). During the second visit, previously measured trees are re-identified and classified as alive (survivor), dead (deceased), or harvested, allowing direct estimation of demographic changes. In addition, newly recruited trees that have grown above the 7.5 cm DBH threshold since the first visit are also recorded. Although each tree is revisited only once, annual mortality rates can be estimated by aggregating observations of multiple sites across regions, and species within a rolling 5-year window (see below). In addition to dendrometric data, field teams record information on stand structure, site ecology (e.g., floristic composition, soil type, and slope), and forest management practices within a 25-m radius.

To estimate annual stem-based tree mortality rates from the NFI data, we used observations from trees measured during two visits, 5 years apart. Because the exact timing of death within the 5-year interval is unknown, we calculated mean annual mortality rates (*m*) using the standard formula^79^:

$$m=\left(1-{\left(\frac{{N}_{t1}}{{N}_{t0}}\right)}^{\frac{1}{T}}\right)\times 100$$

where *N*_*t0*_ is the number of trees alive at the first visit, *N*_*t*1_ is the number of individuals that survived between visits, and *T* is the revisit interval (i.e., 5 years). Trees recorded as harvested were treated as survivors, which likely results in an underestimation of true mortality, as some of these trees may have died naturally and been removed (e.g., through salvage logging during bark beetle outbreaks). To assess this potential bias, we compared three scenarios: treating harvested trees as survivors, as dead, or excluding them entirely (Fig. S3). This sensitivity analysis revealed that, under the assumption that salvage logging was widespread, true mortality rates could be substantially higher than the conservative estimates presented here. Excluding harvested trees from the mortality calculation yielded similar mortality trends to those obtained when treating them as survivors.

To quantify uncertainty in the observed annual mortality rates from the NFI’s rotating sampling scheme and variation in spatial and temporal coverage, we applied bootstrapping, following previous studies^80^. Trees were grouped by species, region, or diameter class and by census year. Within each group, we generated 100 bootstrap samples by resampling trees with replacement. For each sample, we calculated annual mortality rates and then derived the mean and standard deviation across all samples (Figs. 2 and S1–S5). To put these mortality rates into perspective, we applied the same bootstrapping method to estimate absolute changes in basal area caused by mortality, harvest, and growth (including growth of survivors and ingrowth through newly recruited trees during the second visit, Fig. S4).

For the analysis of regional trends, we grouped trees by the IGN’s greater ecoregions^81^, broad biogeographic zones with similar geology, relief, climate, soils, and potential natural vegetation—and year of first measurement. Since we resampled data with replacement during bootstrapping, more common species contributed more observations. Thus, regional mortality trends were inherently weighted by species occurrence, accurately reflecting the composition of forests visited in each region and year. The bootstrapping procedure directly quantifies uncertainty related to data abundance. Thus, species with fewer observations (e.g., *Ilex aquifolium*) or regions with low forest density or low accessibility (e.g., Grand Ouest, Pyrenees) showed wider uncertainty ranges (Figs. 2 and S1–S5).

### Datasets for mortality modeling

To quantify the importance of climate anomalies, forest dynamics, and other potential drivers of tree mortality, we applied random forest models to classify whether individual trees survived or died during the 5-year revisit interval. This approach builds on earlier work using tree- and stand-level properties alongside climate data (for example refs. ^15,33,35,40–42^), but extends it by combining a much broader feature space with a machine-learning approach.

For the French NFI data, we applied the following filters to detect multiple mortality pathways while reducing noise from errors in the dataset. We removed sites and trees that were not part of the sampling protocol (original variables in the NFI data: PEUPNR and CIBLE), sites without coordinate information, and trees without live state assessment (original variables VEGET and VEGET5). The identification of dead trees was revised in 2009, and we thus kept only data obtained with a consistent sampling protocol after 2010 (i.e., first visits from 2010 to 2017, respectively, second visits from 2015 to 2023). To avoid introducing bias, we retained sites with irregular mortality events (e.g., storm-affected plots), as such events may be linked to underlying climate anomalies^6^. Similarly, we retained sites with sparse tree counts (e.g., fewer than four trees >7.5 cm DBH, comprising 6% of sites), because random forest models are generally robust to such sampling outliers^39^. Also, we did not differentiate between background and acute mortality events, as our aim was to capture the integrated mortality risk linked to seasonal climate anomalies across all event types. This decision reflects the ecological reality that acute events may amplify background stress and vice versa, and that their interactions cannot be easily disentangled given the 5-year revisit interval of the French NFI. Subsetting data likely introduces artefacts and modeling bias^37^, so we instead relied on extensive random sampling and model replication to isolate consistent mortality signals. The final dataset for mortality modeling included 566,840 trees (28,975 died between visits) from 48,718 sites and 52 species.

To broadly assess the main drivers of mortality, we compiled 183 features across eleven categories, informed by previous studies. All features not derived from the NFI data itself were spatially matched to forest plots using 1 km gridded products (see Supplementary Data 2 for a detailed description of data sources and how each feature was quantified). At the tree level, we included features to describe a tree’s vigor^12^ using DBH or height as proxies for age^27^ (Fig. S16). We quantified light competition using a tree’s canopy position (suppressed trees tend to have higher mortality rates^27^). We included measures of within- and across-species competition, which influence resource access differently^29^. At the stand level, we included structural and demographic attributes, such as size and age distributions and basal area density, which regulate resource partitioning and thus shape both stand resilience and individual risk^53,82^. At the site and landscape level, we considered NDVI as an indicator of canopy decline^71,83^, topography (e.g., greater water stress on south-facing slopes), soil water-holding capacity (potentially buffering drought impacts^44^), and soil fertility (higher nutrients may reduce root investment and increase drought vulnerability^84^). Further, we included management indicators as thinning may reduce light and water competition. Biotic agents, especially pest outbreaks, are also major drivers of mortality^85^, although spatially explicit pest data were unavailable for inclusion.

In addition to these biotic and abiotic drivers, we focused on climate-related features (temperature and CWB) that quantify how deviations from a site’s long-term climate norm affect mortality^10,15^. Because trees do not necessarily die in the same year that they experience climatic stress, capturing the relevant timescale of exposure is crucial. Numerous studies have shown that tree mortality often lags behind stress events like droughts by several years^9,11,86,87^. Moreover, the NFI’s 5-year revisit interval adds further uncertainty to the precise year of death. To account for both lagged responses and uncertain timing, we considered not only the climate anomalies occurring between two successive visits but also those from the 2 years preceding the first census. This approach captures climate conditions roughly 2–7 years before observed mortality. While the length of lag effects likely varies across species, stress types, and site conditions, this 7-year window represents a biologically meaningful range^64^. Previous studies support this timescale, both in terms of ecological response^86^ and modeling practice^30,87–89^. Observations from the 2018 European drought further support this choice, as widespread tree death occurred not only during the drought but also in the years that followed^9,11^. We commonly refer to this 7-year window as “short-term climate conditions” and quantified these conditions for each forest plot as anomalies relative to the plot’s long-term reference climate from 1960 to 2020. We selected this reference period because it spans the full range of available data and has proven effective in characterizing tree mortality risk in prior studies^13,15–17^.

To calculate seasonal temperature anomalies, we used monthly mean, maximum, and minimum temperature data and calculated standardized seasonal anomalies relative to the reference climate period. Standardization expresses anomalies in units of site-specific variability, such that a unit change corresponds to one standard deviation from the local long-term seasonal mean, allowing comparison across regions, seasons, and temperature metrics. From the 7-year window preceding each plot’s revisit year, we extracted the lowest anomaly, the highest anomaly, and the mean of anomalies, representing the seasonal low, peak, and the average temperature anomaly from the reference climate (see Fig. S17).

To calculate seasonal CWB anomalies, we used monthly data of the standardized precipitation-evapotranspiration index (SPEI)^90^, which is a meteorological drought index that estimates the aggregated water surplus or deficit based on the difference between precipitation and potential evapotranspiration, relative to the reference climate period. SPEI is a standardized index, such that a unit change represents one standard deviation wetter or drier than the local climatic norm over the selected timescale. We calculated SPEI using nine different aggregation timescales to capture both short-term extremes and long-term shifts (1, 3, 6, 9, 12, 15, 18, 21, and 24 months). As with the temperature anomalies, we extracted the lowest anomaly, the highest anomaly, and the mean of anomalies from the 7-year window, representing the seasonal low, peak, and the average water availability anomaly from the reference climate (see Fig. S18). Although vapor pressure deficit is also a relevant indicator of atmospheric drought stress^10^, we used SPEI as our drought index because it is commonly used in tree mortality modeling^10,13,30^ and provides valuable temporal information.

With all features compiled, we next aimed to model tree mortality using a refined set of biologically meaningful features. The initial pool of 183 variables was not intended for inclusion in full in any single model but rather served as a broad hypothesis space to identify the most relevant and consistent predictors of mortality across species. To balance model complexity with ecological interpretability and to avoid inflated importance scores due to unequal feature counts across categories, we applied recursive feature selection. This process, visualized in Fig. 1 and explained in detail below, retained the single most important feature per feature category, resulting in a final, parsimonious model composed of eleven non-correlated features for each species and randomization seed.

### Tree mortality modeling

To predict individual tree mortality across a wide range of species and conditions, we developed a machine learning modeling framework tailored to address the unique challenges of tree mortality prediction. To identify consistent responses across species while accounting for variability in the underlying data, we followed an ensemble-based approach. Specifically, we fitted 50 random forest models per species, each using a non-correlated feature set consisting of one feature per mortality feature category. Across these 50 models, we varied the random seed to alter train-test splits, class balancing, and random forest bootstrapping, allowing us to assess the consistency of model outcomes across different data realizations.

We employed random forest models^39^ to classify trees of each species in the NFI data as either having survived or died between visits (i.e., predicting the probability of dying between visits). Random forests build many decision trees, each trained on a random bootstrapped subset of the data and features, and then aggregate their predictions to estimate probabilities. This random subsetting disrupts correlations in the data and among predictors, while the ensemble of decision trees flexibly captures nonlinear relationships and interactions. Together, these properties also make random forests robust to noisy data and outliers, offering a reliable framework for modeling tree mortality across diverse species and conditions^37^.

The modeling procedure began by splitting each species’ dataset (all trees measured across all censuses) into training (80%) and testing (20%) sets, with balanced proportions of surviving and dead trees (see Figs. 1 and S19 for visual summaries). Since tree mortality is a rare event, the data is inherently imbalanced, with many more surviving trees than dying, leading to a biased overfitting towards surviving trees^41^. To address this, we applied the synthetic minority oversampling technique (SMOTE), which balances the training set by generating synthetic samples of the minority class (dead trees) using a k-nearest neighbors algorithm^91^. Oversampling was applied only to the training set to prevent data leakage and artificially inflated performance. To confirm that the synthetic samples remained ecologically meaningful, we assessed overlap in multivariate space by comparing PCA projections and Mahalanobis distances between the original and SMOTE-augmented training sets, as well as against the test data (Figs. S20 and S21). Missing values in any feature were imputed using the respective feature’s mean value from the training set.

The balanced training set was then used in a recursive feature selection process, where random forest models iteratively identified the most relevant feature in each of the eleven feature categories. At each iteration, a random forest classifier was fitted to the training data and optimized by minimizing the out-of-bag (OOB) error. Then, features were ranked by importance, measured by the mean decrease in Gini impurity across all decision trees of the random forest. We also tested permutation-based feature importance and found nearly identical results. In each iteration, we removed the least important features—five at a time if more than 100 features remained, and one at a time thereafter—while ensuring that at least two features per category were preserved until only 22 features (two per category) remained. This safeguard prevented the premature removal of mechanistically plausible features and ensured that larger feature groups did not disproportionately dominate model outcomes. The selection then continued until a single best feature per category was retained, resulting in eleven final features that represented the full ecological feature space.

Following this recursive feature selection, we explicitly removed linearly correlated features (Pearson’s *r* > 0.8) across the eleven retained variables, keeping only the one with the higher importance score. This step was most relevant for categories with potential redundancy, such as competition metrics or climate-related variables, where correlations could bias interpretation. Although random forests already reduce the impact of correlated predictors, we added this safeguard to further enhance interpretability. In practice, it affected only a small fraction of models: cross-category correlations were primarily related to categories describing competition and led to feature removal in about 3% of the 450 models for the nine most common species and about 20% of the 2600 models across all 52 species (Supplementary Data 1).

With the final set of non-correlated and ecologically diverse predictors, we tuned the random forest hyperparameters through a grid search to minimize the OOB error. The search varied three key parameters: the number of trees (*n*_estimators, set to 100 or 300), the maximum tree depth (max_depth, between 1 and 18), and the fraction of features considered at each split (max_features, set to 1%, 10%, or the square root of the total feature count). The final model was then fitted with the optimized parameters on the SMOTE-balanced training data and evaluated on the held-out test set.

To account for the inherent randomness in several parts of the modeling process (including random train-test splits, SMOTE sampling, and stochasticity in bootstrapping and feature selection within the random forest algorithm), we repeated the full modeling routine 50 times per species, changing only the random seed in each run. This ensemble approach mitigates the issue of equifinality, where different feature combinations may yield equally good predictions, reflecting the complexity and challenge of modeling tree mortality^27^. It allows us to characterize the variability and robustness of model performance and feature selection, while reducing the risk that results are biased by residual spatio-temporal autocorrelation in the NFI data. Finally, this approach directly addresses concerns in the literature that single-model studies may underestimate uncertainty in variable importance^37^.

For each model, we evaluated performance using the area under the receiver operating characteristic curve (ROC-AUC), a standard metric in tree mortality modeling (Supplementary Data 4), which quantifies how well a classifier distinguishes true positives from false positives (with 1 indicating perfect classification and 0.5 indicating random classification). The average ROC-AUC on the held-out test set across all species and model runs was 0.73 ± 0.05, consistent with values from previous mortality studies using random forest^37,40,41^ or logistic regression models^15,30^. To ensure comparability across studies^30^, we retained only models with ROC-AUC > 0.6, indicating that the model could distinguish mortality from survival with at least 60% probability. For the nine most common species, about 2% of the 450 models were excluded based on this threshold (i.e., 8 models); across all species, about 10% of the 2600 models were excluded (i.e., 253 models). Most of the excluded models belonged to species with limited data availability and high variability in predictive performance across model replicates (e.g., *Ilex aquifolium*), whereas species with larger sample sizes yielded higher and more stable performance (Fig. S22).

### Feature importance and climate influence assessment

To interpret which features most strongly influenced tree mortality predictions across species and model replicates, we used SHAP, a game-theoretic approach that quantifies each feature’s marginal contribution to individual predictions^43^. For each prediction, the SHAP value reflects how much a specific feature value, compared to its average contribution across the dataset, shifts the predicted probability of mortality relative to the model’s baseline expectation (i.e., the mean predicted probability across the dataset). Positive values indicate higher predicted mortality risk, while negative values indicate lower risk. SHAP values were computed using the interventional “TreeExplainer” algorithm implemented in the “shap” Python package (Version 0.45), which is optimized for tree-based models such as random forests and accounts for feature dependencies^43,92^. Calculations were based on the held-out test dataset for each model, ensuring that results reflect generalizable model behavior rather than training artefacts.

For each final random forest model, we computed SHAP values for all predictions and calculated the mean absolute SHAP value for each feature, representing that feature’s overall contribution to mortality predictions. To enable comparisons between features both within and across models, we normalized the mean absolute SHAP values to sum to 100%, giving each feature a relative importance share. For Fig. 3A, we then averaged the relative importance across the 50 replicates per species and displayed the species-level distributions as boxplots.

Because each model retained only one temperature feature and one CWB (SPEI) feature, we could link mortality responses directly to specific climatic conditions. To robustly detect climate-driven mortality signals, we classified each model’s climate response based on its SHAP values and corresponding climate anomalies. For each model, we tested for a monotonic trend between SHAP values and the associated climate feature using Mann–Kendall trend tests. If the trend was significant (*p* < 0.01), we assigned a directional response indicating increased mortality risk: for temperature, “warmer” or “cooler”; for CWB (SPEI), “drier” or “wetter.” If the trend test was not significant, the response was classified as “other.”

We then assessed whether these climate-mortality responses were consistent across models that shared the same temperature or SPEI feature pair. If more than 60% of models within a given pair showed the same directional response (e.g., warmer-drier leading to increased mortality), we classified the pair as consistent. Otherwise, we labeled it as “other” to avoid overinterpreting idiosyncratic model behavior. This 60% threshold ensured that only robust mortality signals were propagated to summary figures (e.g., Figs. 3–5). Raising the threshold to 90% increased the fraction of models classified as inconsistent from 3 to 10% overall (and from 0 to 5% for the nine most common species) but had a negligible influence on the main results.

To interpret which types of climate anomalies underpinned these consistent climate responses, we examined the characteristics of each temperature and SPEI feature, defined by three attributes. Temperature features were characterized by (i) the season during which the anomaly occurred (winter, spring, summer, or fall), (ii) the temperature metric used (monthly mean, minimum, or maximum), and (iii) the anomaly type (coldest anomaly, hottest anomaly, or average temperature anomaly). CWB features were defined by (i) the season (corresponding to the final month of the SPEI integration period), (ii) the integration timescale or event duration (ranging from 1 to 24 months), and (iii) the anomaly type (driest anomaly, wettest anomaly, or average water availability anomaly). This structured feature design allowed us to analyze which combinations of climate anomalies (e.g., on-average warmer winters or more intense droughts) were most consistently associated with increased mortality risk. To visualize emerging patterns, we decomposed each temperature and CWB feature into its three attributes and calculated the frequency of each attribute combination across models. We visualized these frequencies in Figs. 4, 5, S12, and S13 as pie charts, where colors represent anomaly types, the y-axis shows the season, and the x-axis indicates either the temperature metric or the SPEI integration timescale. For each species, we also summarized the frequency with which models identified specific anomalies and their components (Supplementary Data 5 for temperature, Supplementary Data 6 for CWB, and Supplementary Data 7 for their combination).

To quantify the magnitude of climate-mortality relationships identified by the models, we estimated local marginal effects of seasonal climate anomalies on predicted mortality risk. For each model, the retained temperature or CWB anomaly associated with increased mortality risk was perturbed by one standard deviation in the direction opposite to the modeled response, while all other predictors were held constant (e.g., comparing an observed summer to one that is one standard deviation less warm). Mortality risk was then recomputed for each tree under the perturbed condition and compared to the prediction under observed conditions, yielding a local estimate of the change attributable to the anomaly. To avoid extrapolation beyond the observed predictor space, perturbed observations falling outside the range present in the corresponding training data were excluded. Because models were trained on standardized anomalies and random forest responses are nonlinear, these estimates represent local marginal effects and should be interpreted as local sensitivity measures rather than effects that scale linearly with anomaly magnitude. We also evaluated perturbations in the direction of more extreme conditions (i.e., one standard deviation further in the mortality-promoting direction). Although this produced qualitatively similar patterns, it resulted in substantially more extrapolation beyond the training range. We therefore report results based on perturbations toward less extreme conditions, where predictions were supported by a larger proportion of observed data.

### Species- and tree-level analyses

To better understand how different species responded to climate anomalies, we calculated the percentage of models (i.e., model frequency) per species that indicated increased mortality under different short-term climate anomalies (warmer-drier, warmer-wetter, cooler-drier, cooler-wetter, and other). We used these percentages as input for a principal component analysis (PCA, Fig. 7A), which shows how species differ in their overall climate response profiles, with arrows indicating the contribution of each anomaly type to the principal axes.

To test whether species-level responses to climate anomalies could be explained by functional traits, we regressed the percentage of models linking mortality to drier or wetter conditions—and, within warmer subsets, to warmer-drier or warmer-wetter conditions—against three species traits: a species’ mean height calculated from all trees of that species in the NFI dataset (Fig. 7C), drought tolerance index (Fig. 7B), and shade tolerance index (Fig. 7D), with the latter two indices stemming from Niinemets et al.^46^. These tests help clarify whether mortality associated with unusually wet conditions reflects physiological trade-offs linked to growth potential, drought avoidance, or competitive strategies.

To assess tree-level responses within species, we analyzed SHAP values for the selected CWB features across models. This allowed us to test whether trees with higher predicted mortality risk under drier conditions also tended to show higher risk under wetter conditions, which could suggest a shared mortality mechanism. To enable comparison across models, SHAP values for CWB features were normalized within each model and then averaged across all models with the same CWB-mortality response (drier or wetter) for a given species, yielding one mean normalized SHAP value per tree.

We interpreted these mean normalized SHAP values as each tree’s sensitivity to drier and wetter conditions, reflecting the marginal effect of CWB anomalies on predicted mortality risk relative to other features. For each species, we compared these sensitivities with bivariate density plots and quantified their association using Spearman rank correlations (Fig. 8). To quantify how many trees contributed to these correlations, we calculated the proportion of individuals that appeared in the test sets of both drier and warmer-wetter models. We then tested whether species-level height patterns extended to the individual level by regressing tree-level sensitivities against height, thereby assessing whether taller individuals consistently faced higher risk under wetter conditions (Fig. 8 and Supplementary Data 9). Furthermore, to assess how the importance of CWB and temperature features for predicting mortality risk changed over time, we grouped sensitivity scores (i.e., mean normalized SHAP values) for each feature by anomaly type, species, and census year, and visualized the mean ±95% confidence intervals (Fig. S9).

### Sensitivity analysis

Because ROC-AUC measures whether trees that died were generally assigned higher predicted mortality risks than trees that survived, rather than how well models classified individual mortality events, we also evaluated performance using precision, recall, PR-AUC, and F1 score. These metrics are particularly informative under class imbalance and capture complementary aspects of minority-class prediction. Recall quantifies the fraction of observed mortality events detected by the model, precision quantifies the fraction of predicted mortality events that were correct, and the F1 score summarizes the balance between both. Across all species and models, precision was 0.22 ± 0.14, recall was 0.45 ± 0.11, PR-AUC was 0.27 ± 0.16, and F1 score was 0.27 ± 0.12 (mean ± sd). Together, these metrics show that the models captured mortality-related signals but remained limited in their ability to classify individual mortality events. This highlights the broader difficulty of predicting rare and complex ecological outcomes such as tree mortality, and may partly explain why precision-recall metrics remain less commonly reported in tree mortality studies^37,38^.

To assess the robustness of our modeling framework, we re-ran the full analysis for the nine most common species under three sensitivity settings. First, we tested whether retaining more than two features per category prior to final selection altered model outcomes. Repeating the analysis with four or six retained features per category yielded nearly identical feature sets and model performance (Figs. S23–25). Second, we varied the number of nearest neighbors in the SMOTE algorithm (*k* = 1 and *k* = 15, compared to the default *k* = 5). Model performance remained stable across SMOTE settings (Fig. S25), although higher *k* values produced slightly more models linking mortality to wetter conditions, without changing the overall frequency and distribution of identified seasonal climate anomalies (Figs. S26 and S27). Third, we tested models without synthetic oversampling, instead balancing the classes by randomly replicating observed mortality cases. Feature importance patterns and rank-based discrimination remained broadly similar (Fig. S28), but models without SMOTE showed a marked reduction in test-set recall and F1 score (Fig. S29). This indicates that, without synthetic oversampling, models detected fewer mortality events and achieved a weaker balance between detecting dead trees and avoiding false positives. Models without SMOTE also identified wetter springs as mortality-promoting less often (about 10% fewer models), suggesting that this response may be linked to a subset of mortality observations that is better represented when SMOTE improves minority-class learning. Together, these sensitivity analyses indicate that our main feature patterns and climate-response structures were robust, while SMOTE improved the ability of the models to capture mortality-related signals in the rare event class. Although it was not feasible to repeat all sensitivity tests for all 52 species, the consistency across the most common species supports the generality and robustness of our results.

As an additional comparison, we fitted 2600 logistic regression models to the full set of 52 species in the dataset. The logistic regression workflow mirrored the random forest analysis as closely as possible: models used the same training and testing splits, the same SMOTE-balanced training data, and the same set of features selected through the random forest recursive feature selection. For each species and run, logistic regression models were fitted to predict mortality risk (i.e., probability to die between two NFI visits), and model performance was assessed on the unchanged test set. Before fitting, features were standardized to obtain directly comparable effect sizes. As for the random forest models, only models with ROC-AUC > 0.6 were kept for further analysis. Feature importance was calculated from the absolute coefficient estimates, divided by the sum of all absolute coefficient estimates, and expressed as relative importance scores between 0 and 100%. Feature importance was then aggregated by mortality driver category and averaged across models for each species. Coefficient signs and p-values were used to classify whether short-term climate anomalies were consistently associated with higher mortality risk under warmer, cooler, drier, wetter, or combined climatic conditions.

Logistic regression models showed broadly similar rank-based performance to random forests, particularly for test-set ROC-AUC (Fig. S30). However, the two algorithms differed in their precision-recall trade-off. Logistic regression achieved higher recall, indicating that it detected more observed mortality events, but this came with lower precision, PR-AUC, and F1 score (Fig. S30). Thus, the higher recall did not reflect uniformly stronger classification performance, but rather a less selective classification pattern with more false-positive mortality predictions. Feature rankings were also broadly consistent between approaches: tree size and competition-related features received slightly more weight in logistic regression, while temperature and CWB anomalies remained among the most important stressor-related features (Fig. S31). Logistic regression also identified significant effects for the majority of climate features selected by the random forest models, and the main climate response patterns were similar, with warmer and drier anomalies most often associated with higher mortality risk (Fig. S31). However, logistic regression produced slightly more cases where the same climate features did not agree on the response direction. This likely reflects the fact that logistic regression assigns a single linear coefficient to each selected feature, whereas random forests can accommodate nonlinear responses, thresholds, and interactions. Climate effects that are context-dependent may therefore appear less stable when summarized by a single coefficient direction. Nonetheless, the logistic regression comparison supports the robustness of the main mortality signals, while showing that algorithm choice primarily affected the precision-recall trade-off and the stability of inferred climate-response directions.

### Reporting summary

Further information on research design is available in the Nature Portfolio Reporting Summary linked to this article.

## Supplementary information

Supplementary Information

Description of Additional Supplementary Files

Supplementary Data 1–9

Reporting Summary

Transparent Peer Review File

## Data Availability

All data used in this study are freely available. Data from the French National Forest Inventory are available at https://inventaire-forestier.ign.fr/dataifn/. The digital elevation model for France is available at https://geoservices.ign.fr/bdalti. Data on temperature, precipitation, evapotranspiration, soil water conditions, and soil pH are available from SILVAE at https://silvae.agroparistech.fr. NDVI data are available from the European Forest Disturbance Atlas^83^ via 10.5194/essd-17-2373-2025. The ESA WorldCover 10 m v200 map and the Global Forest Change v1.10 forest cover map are available through Google Earth Engine. The processed data and code used to produce the results presented in this study are available on GitHub at https://github.com/padasch/tree_mortality_french_nfi and archived on Zenodo^93^ at 10.5281/zenodo.14941688. Supplementary Data are provided with this paper.

## Code availability

Code and data to reproduce study results are available on GitHub at https://github.com/padasch/tree_mortality_french_nfi and archived on Zenodo^93^ at 10.5281/zenodo.14941688.
