# Supplementary material for: Rising tree mortality in France is associated with distinct seasonal climate anomalies
Source: Nat Commun. 2026 Jun 26;17:8016. doi: 10.1038/s41467-026-74613-9 (PMC13454586; doi:10.1038/s41467-026-74613-9)
Supplement: Supplementary file 1 [file 41467_2026_74613_MOESM1_ESM.pdf]

Supplementary Information

This file contains all supplementary figures for the publication:

Rising tree mortality in France is associated with distinct seasonal climate anomalies

Pascal Schneider[1,2,3], Agnès Pellissier-Tanon[3], Chuanlong Zhou[3], Philippe Ciais[3], Christian Piedallu[4], Alba Viana-Soto[5], J. Jelle Lever[1], Arthur Gessler[1,2]*

[1]Swiss Federal Institute for Forest, Snow and Landscape Research (WSL), Forest Dynamics, Birmensdorf, Switzerland

[2]ETH Zurich (Swiss Federal Institute of Technology), Institute of Terrestrial Ecosystems, Zurich, Switzerland

[3]Laboratoire des Sciences du Climat et de l'Environnement, CEA CNRS UVSQ, Gif-sur-Yvette, France

[4]Université de Lorraine, AgroParisTech, INRAE, Silva, Nancy, France

[5]Technical University of Munich, School of Life Sciences, Earth Observation for Ecosystem Management, Freising, Germany

* Email: pascal.schneider@wsl.ch

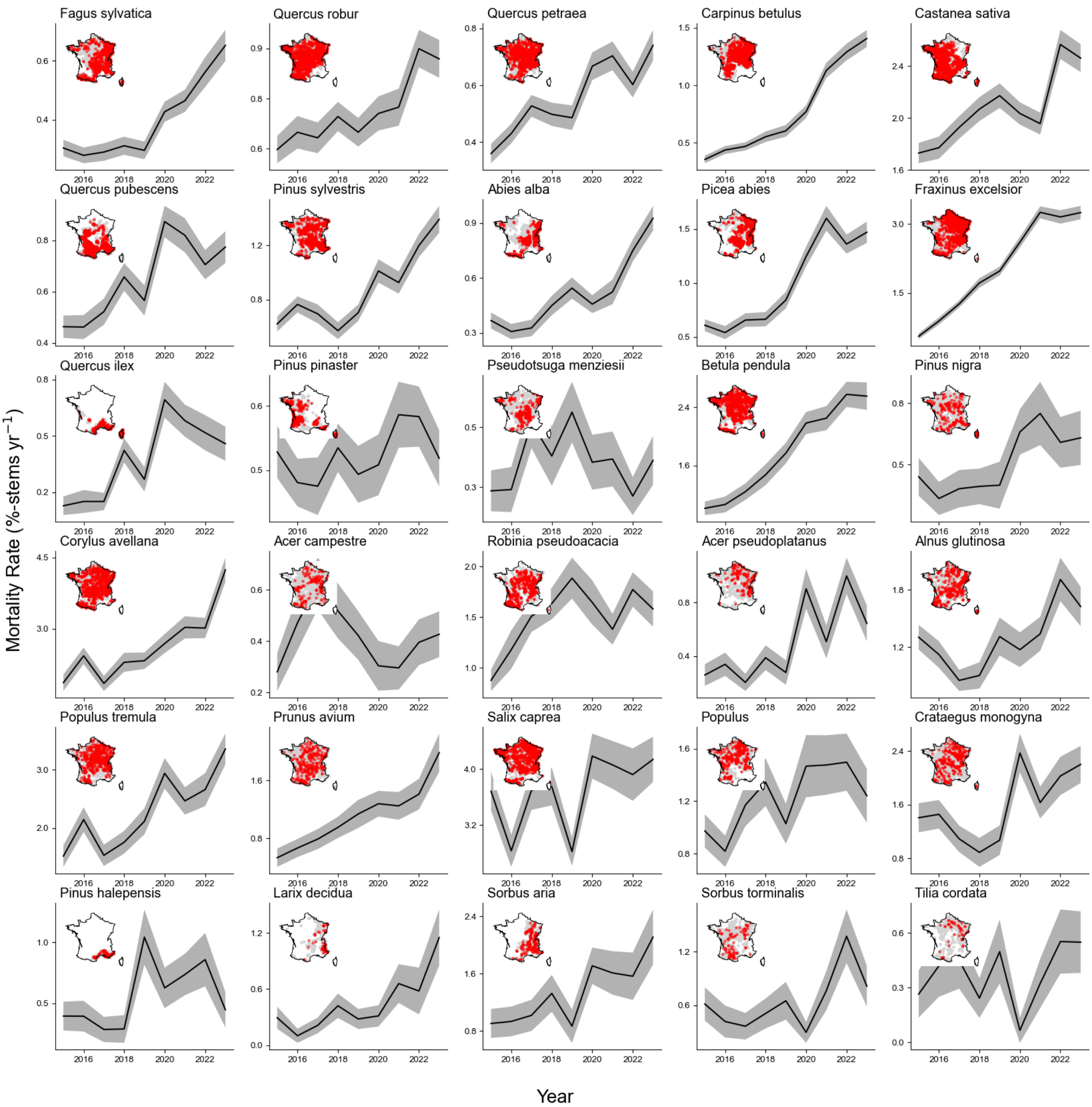

Figure S1. Annual tree mortality rates for all species analyzed in this study, ordered by occurrence from top-left (most abundant) to bottom-right (least abundant). The black line represents the bootstrapped mean, with the shaded band indicating ±1 standard deviation. The inset maps show each species' distribution, with grey areas representing sampled trees and red areas indicating where tree deaths occurred between censuses.

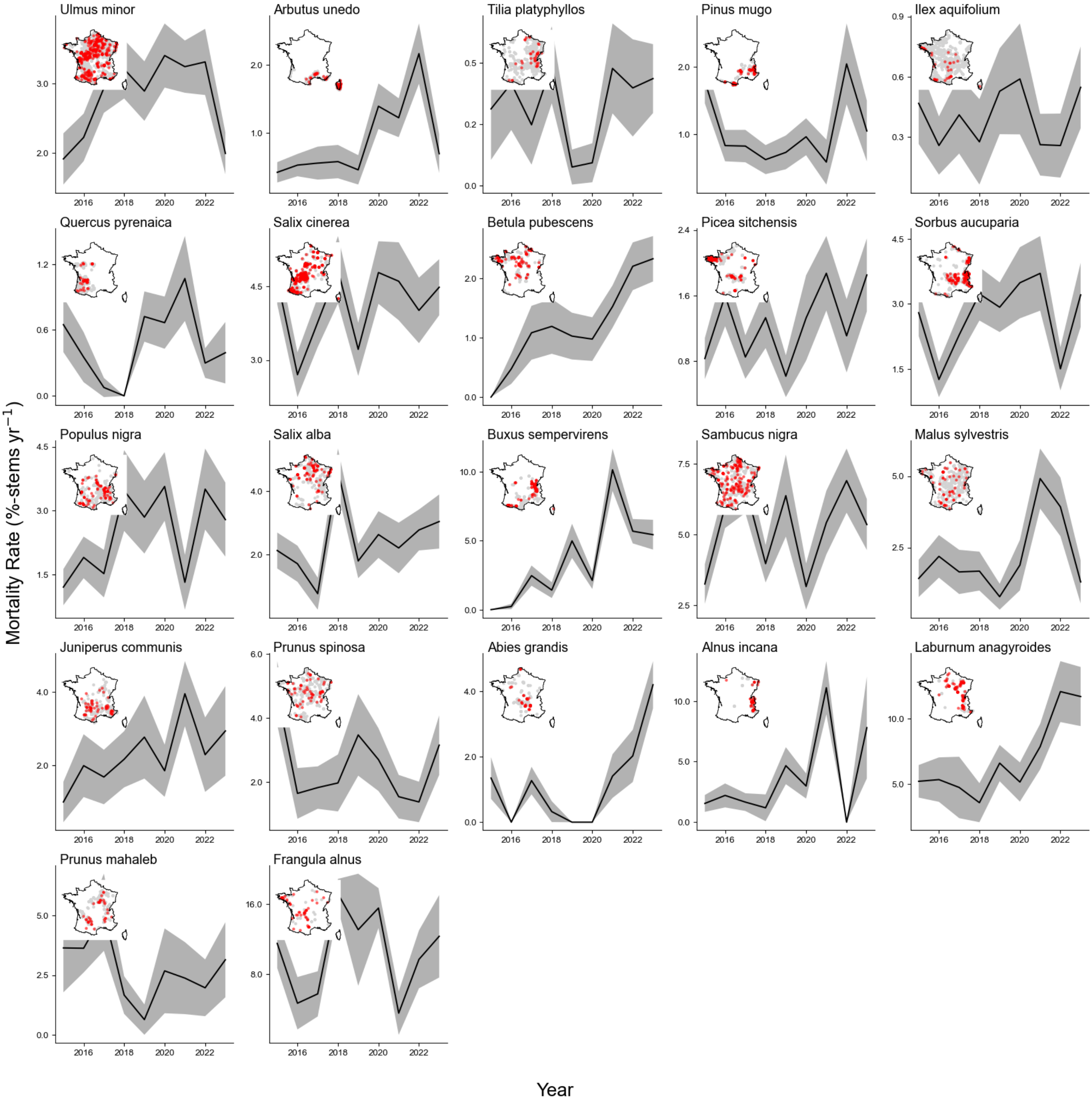

Figure S1. Continued.

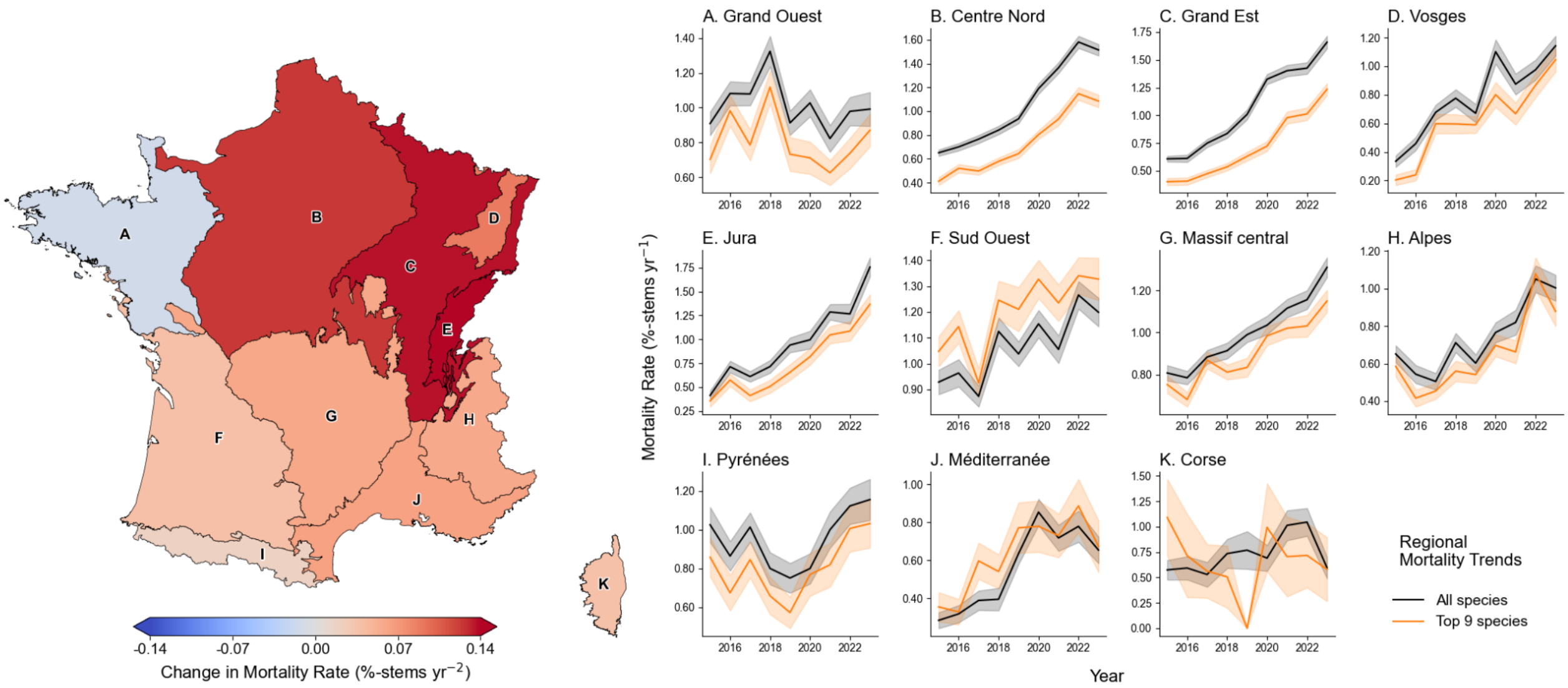

Figure S2. Regional trends in tree mortality across France from 2015 to 2023. Left panel: Map illustrating the change in tree mortality rates across 11 greater ecoregions, expressed as the slope from a linear regression of annual mortality rates over time (equivalent to map for most common nine species shown in main text Figure 2 but using data from all 52 species analyzed in this study). Annual mortality rates were estimated via bootstrapping, resampling individual trees within the given region with replacement. This approach inherently accounts for species abundance, as more prevalent species contribute more to the resampled datasets, effectively weighting the trends by species occurrence. Letters within regions correspond to the enumeration letters in the right panels' titles. Right panels: Time series plots of annual tree mortality rates for each greater ecoregion, showing the bootstrapped mean (solid line) and one standard deviation (shaded area). Black lines represent estimates based on all 52 tree species included in the study, while orange lines show estimates based only on the nine most common species. This comparison highlights the uncertainty and divergence in regional mortality trends when focusing on dominant species versus all species.

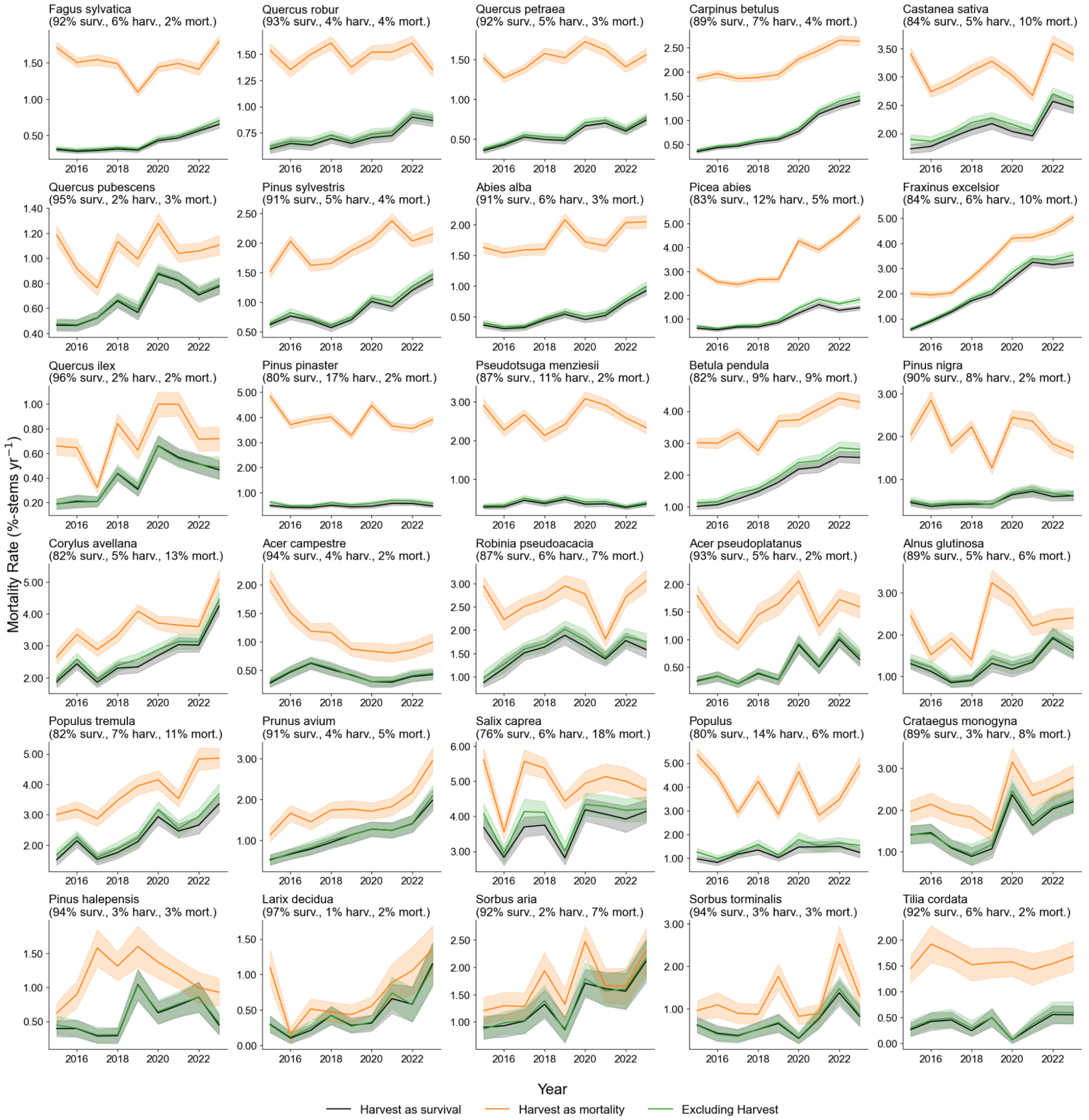

Figure S3. Sensitivity of annual stem-based mortality rate estimates (% stems yr^{-1}) to different treatments of harvested trees during calculation for all species analyzed in this study, ordered by occurrence from top-left (most abundant) to bottom-right (least abundant). Lines show annual mortality rate estimates under three calculation scenarios: harvested trees treated as survivors (black), as diseased (orange), or excluded altogether (green). Shaded areas represent one standard deviation across 100 bootstrap replicates (see Methods). Panel labels indicate the overall proportions of trees recorded as surviving, harvested, or dead across all observations for each of the nine most common species.

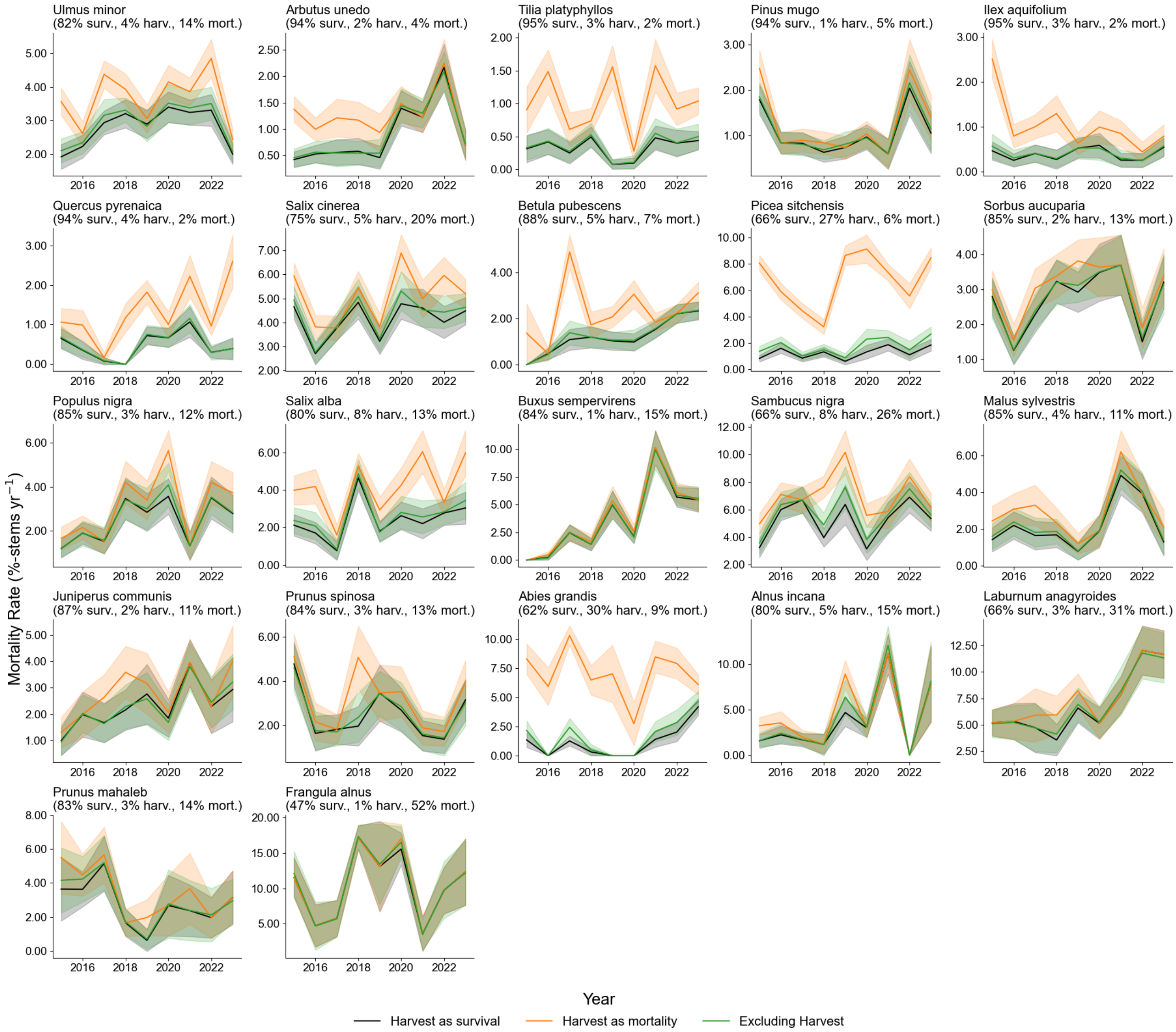

Figure S3. Continued.

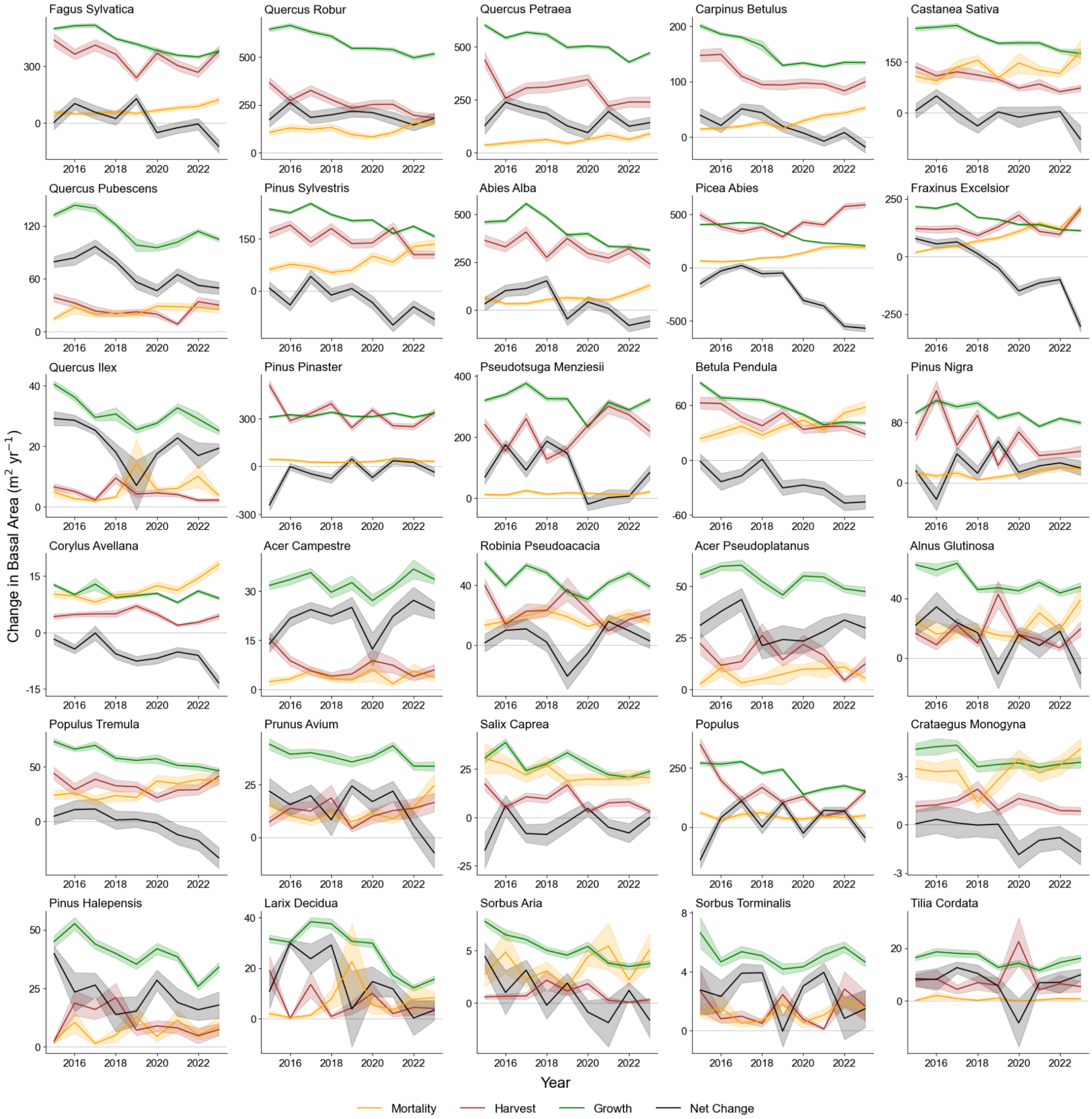

Figure S4. Annual changes in basal area from growth, harvest, and mortality for all species analyzed, ordered by occurrence from top-left (most abundant) to bottom-right (least abundant, with correspondingly smaller basal area values). Lines show mean estimates from 100 bootstrap replicates (see Methods) for basal area gains from growth (green), losses from harvest (red) and mortality (yellow), and the net change (black). Shaded areas indicate one standard deviation, and the dotted black line marks zero change. Growth includes both the growth of surviving trees and the recruitment of new trees between surveys.

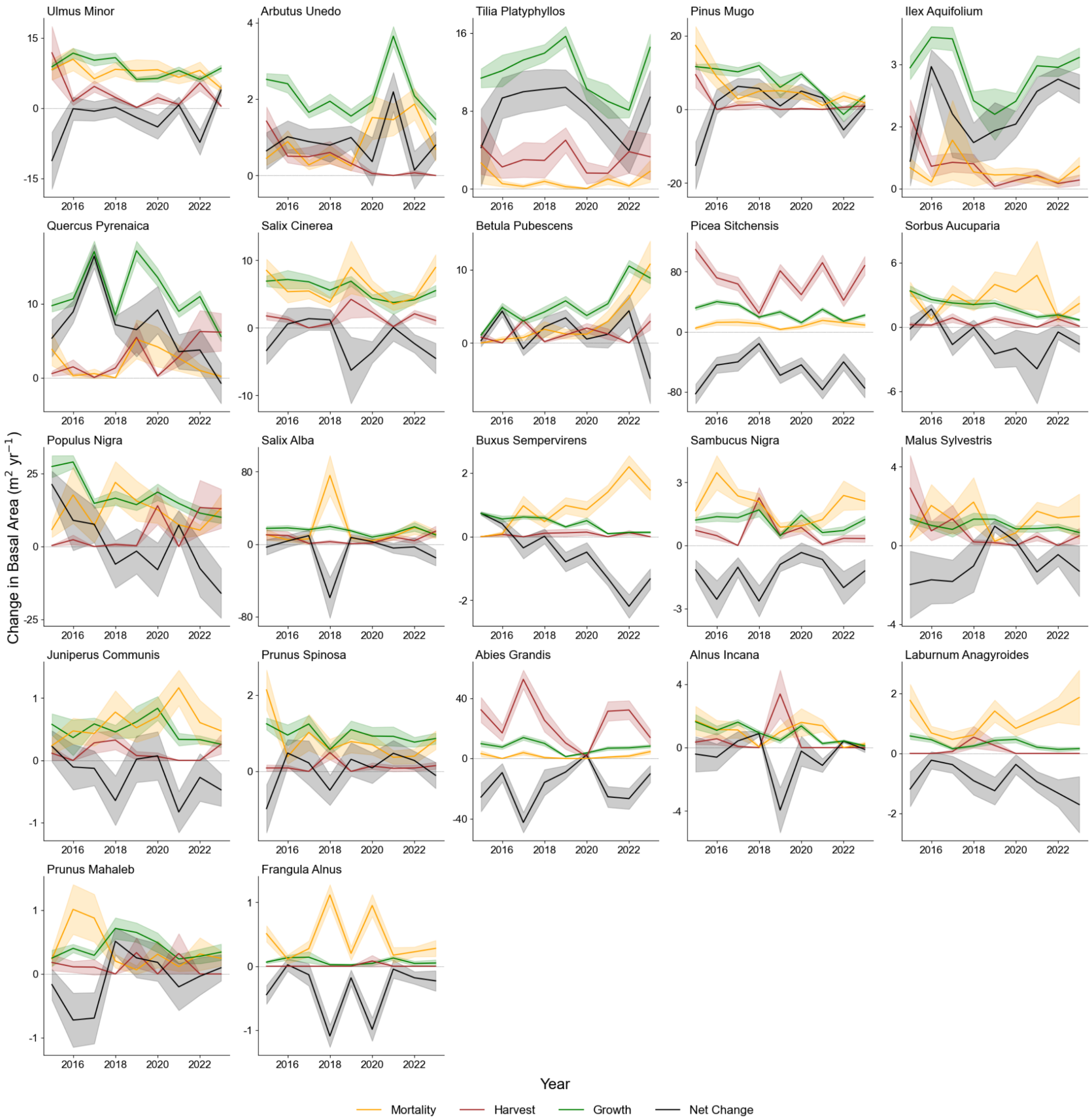

Figure S4. Continued.

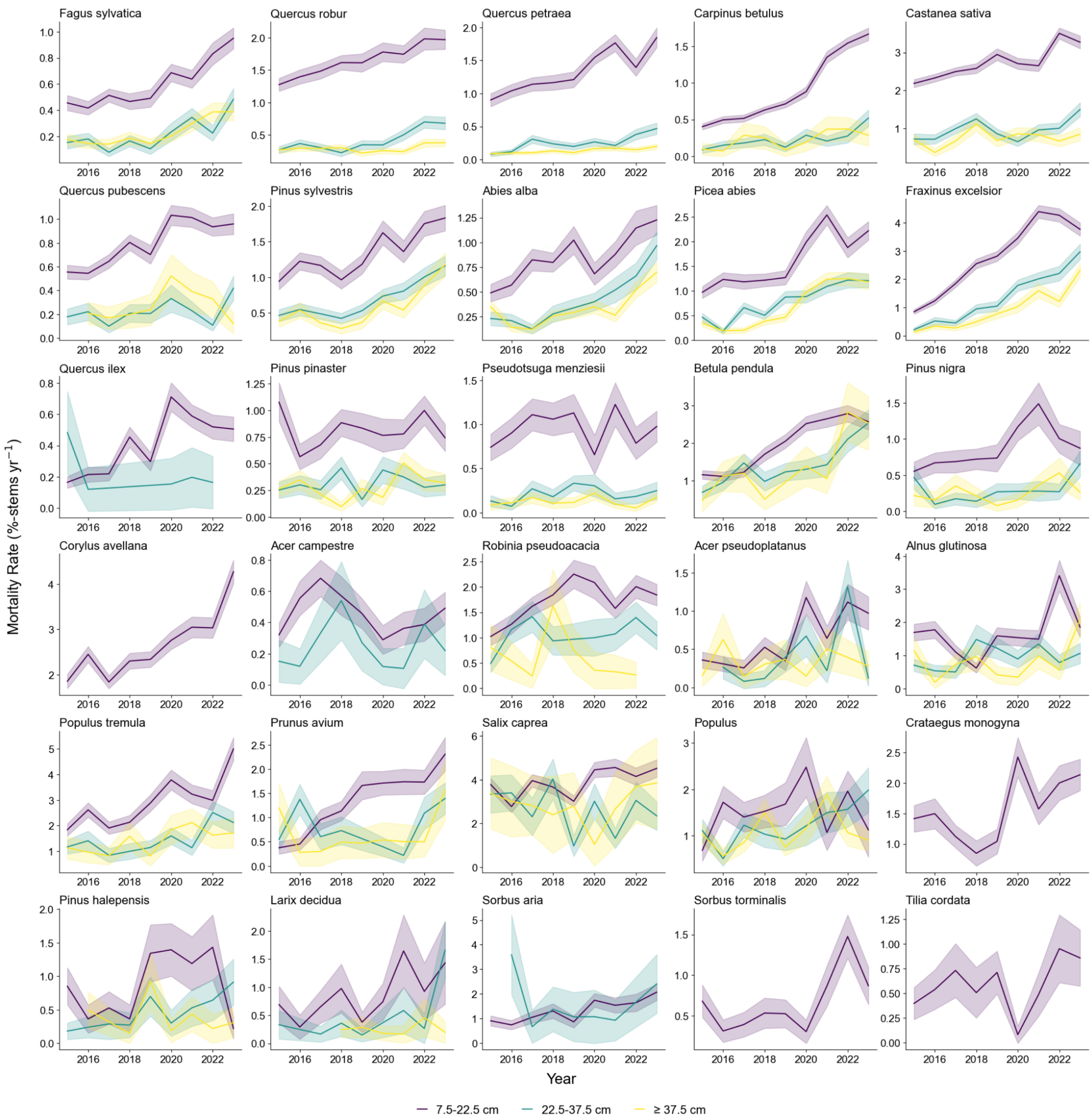

Figure S5. Mortality trends by tree size class for all species analyzed, ordered by occurrence from top-left (most abundant) to bottom-right (least abundant). Lines show annual mortality rates (% stems yr^{-1}) for three diameter-at-breast-height (DBH) classes. Solid lines and shaded ribbons indicate the bootstrapped mean and standard deviation from 100 bootstrap replicates, respectively (see Methods). Uncertainty is greater for individual species-size class combinations due to smaller sample sizes compared to overall species-level estimates, and it generally increases toward less abundant species (see Figure S1). Only time series with at least four years of data are shown to avoid visually noisy trends associated with shorter records with higher uncertainty estimates. Mortality rates are generally higher in smaller trees, whereas larger trees show lower absolute rates but often greater relative increases over time.

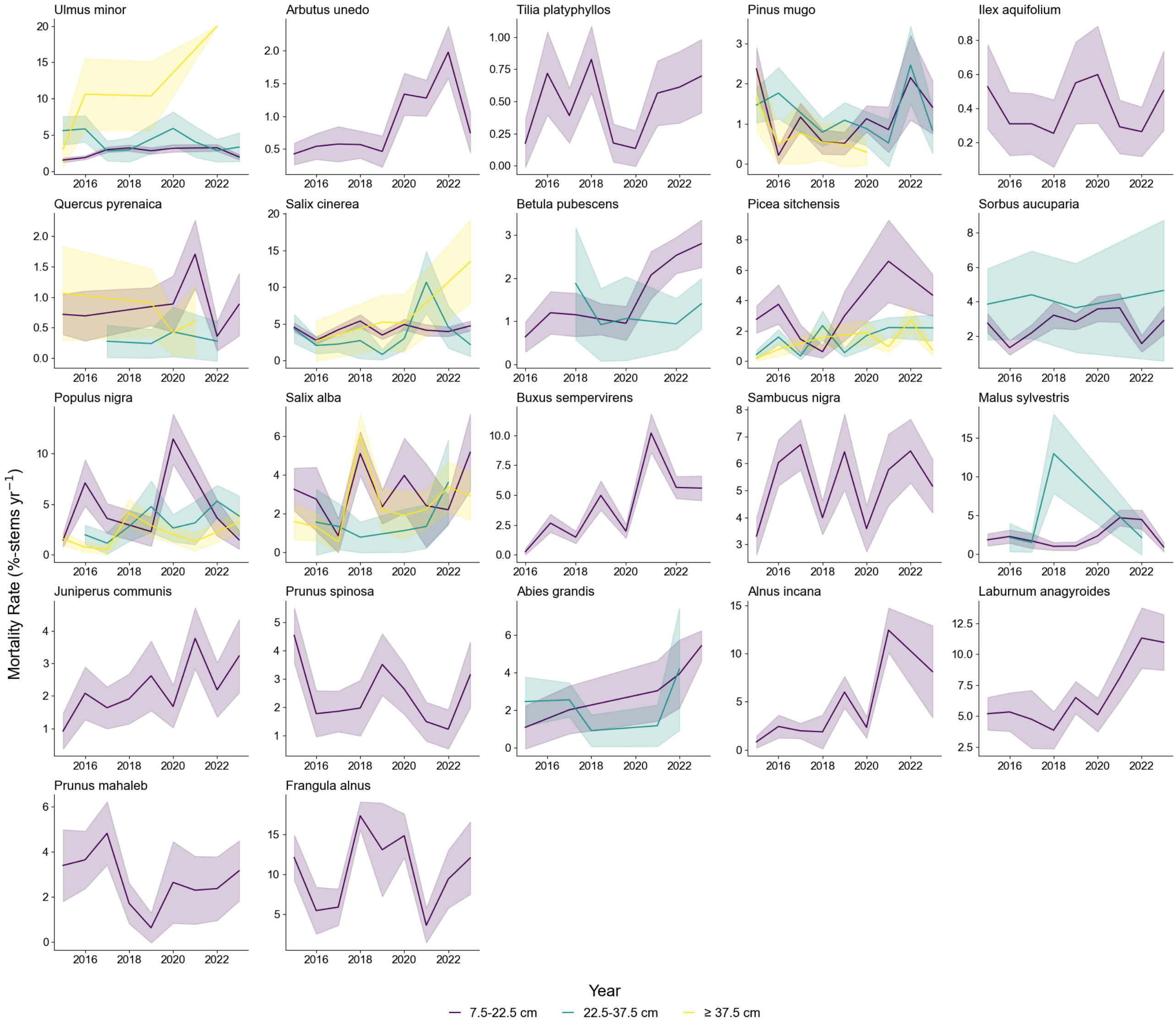

Figure S5. Continued

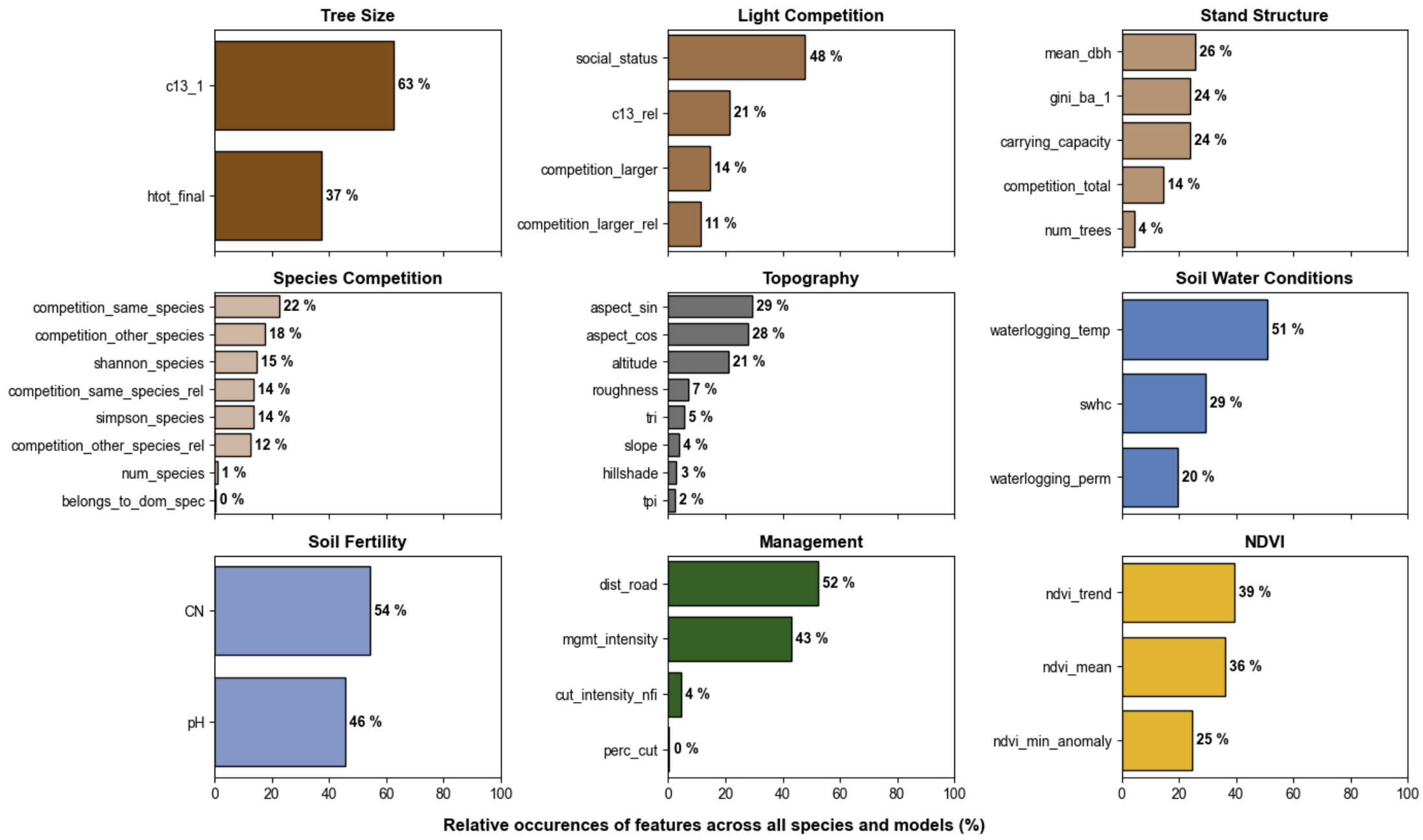

Figure S6. Relative occurrences of features across all species and models. See Table S2 for detailed description of each feature. The importance of each feature across all models and species is given in Table S3. Features for temperature and climatic water balance anomalies are not shown because they are too manifold and thus analyzed in Figures 4, 5, S10, and S11.

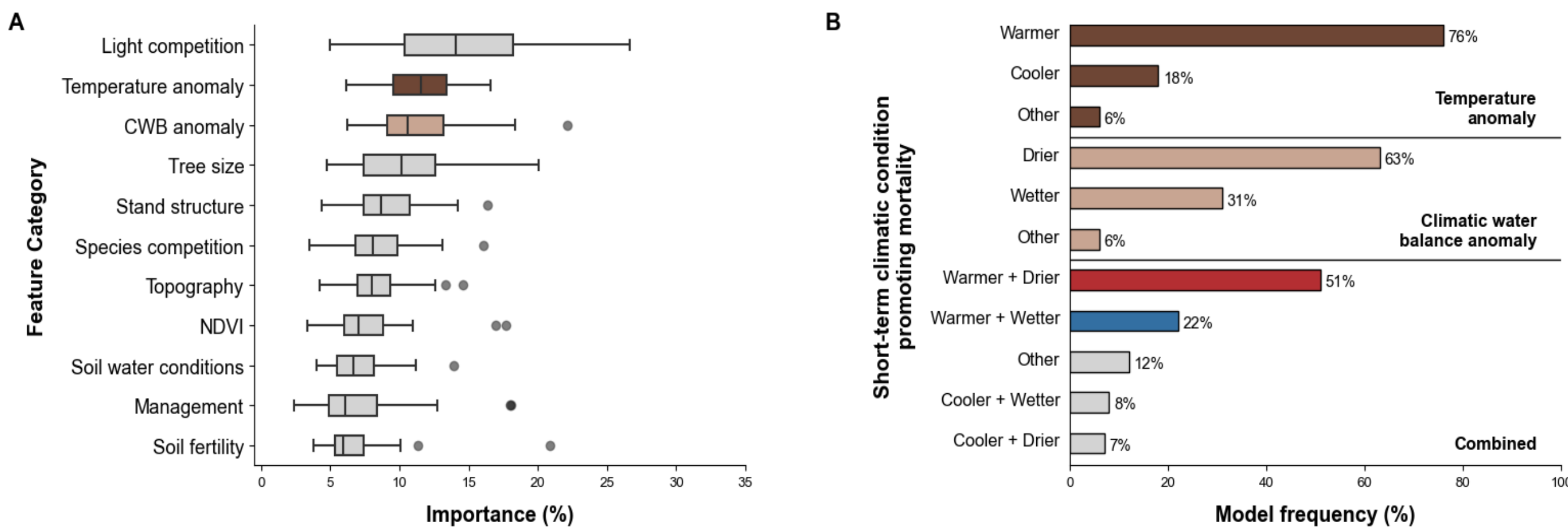

Figure S7. Feature category importance and the short-term climate conditions promoting tree mortality (equivalent to Figure 3 in main text but for all 52 species analyzed in the study). A: Boxplots illustrate the importance of each feature category in predicting a tree's probability of dying between censuses using random forest models. "Importance" refers to the average importance of all features within the same feature category, calculated across all models for a given species (see Methods). Boxplots show the median, 25th and 75th percentiles with whiskers extending to the minimum and maximum values within 1.5 times the interquartile range from the quartiles, and dots represent species outliers. The categories "Temperature anomaly" and "CWB anomaly" are dark and light brown, respectively, matching the colors in panel B (CWB = climatic water balance, NDVI = Normalized Difference Vegetation Index). B: Bar charts show the percentage of models associating mortality with different temperature anomalies, CWB anomalies, or both combined. "Other" denotes models where no significant association was found between a feature and mortality risk or where the same feature did not show a consistent response direction across models (see Methods). The term "short-term conditions" refers to standardized climate anomalies during the years preceding the second tree census. Results for all 52 studies species are shown (N = 2347 models).

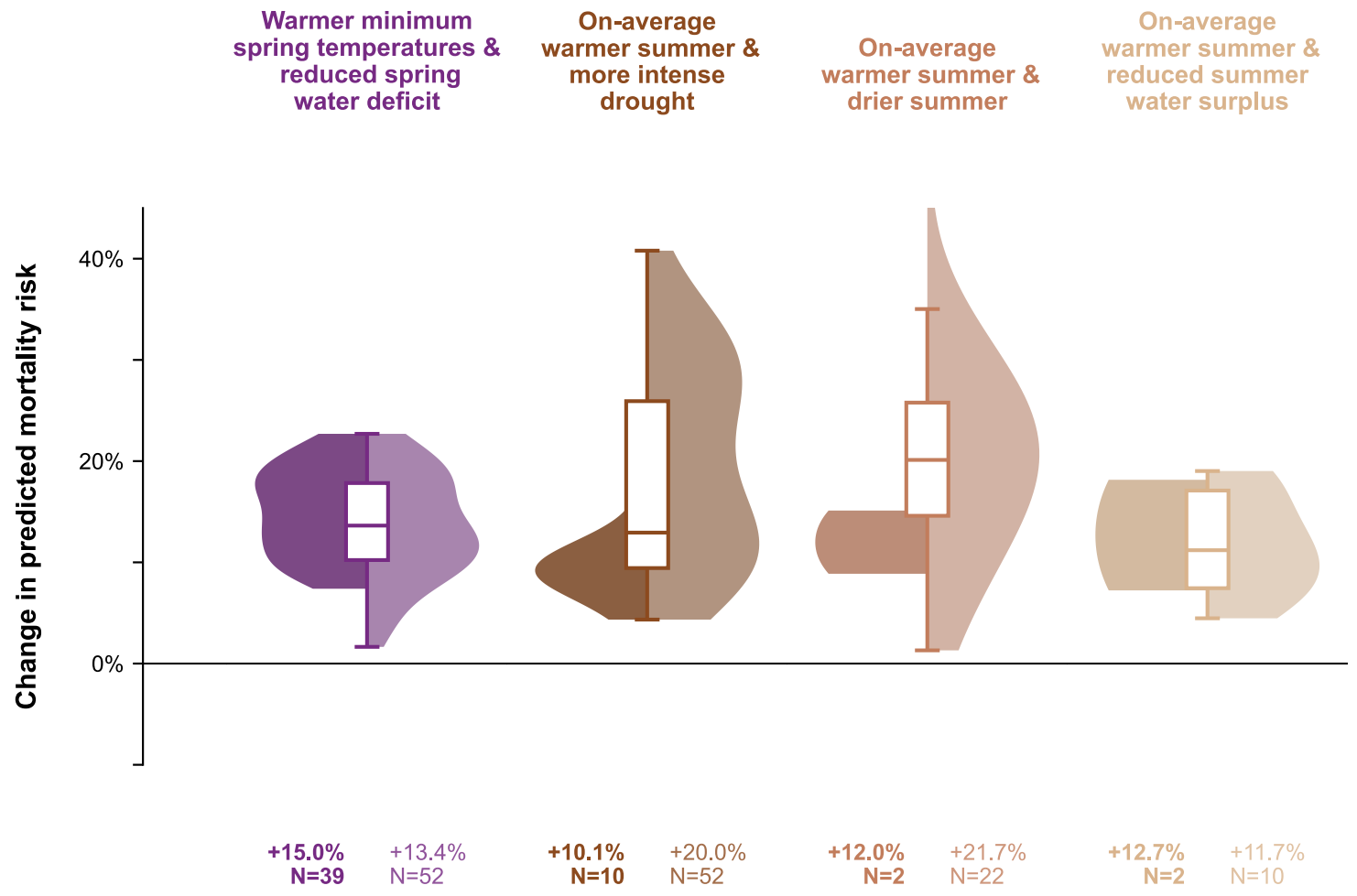

Figure S8. Predicted additional tree mortality risk for combined seasonal climate anomalies. Violin plots show the change in predicted tree mortality risk associated with combined seasonal temperature and climatic water balance (CWB) anomalies, by comparing observed seasonal climate conditions to alternatives where both anomalies were shifted by one standard deviation away from the modelled mortality response (e.g., comparing a warmer and drier summer to a summer that is one standard deviation cooler and wetter; see Methods). The shown combinations represent the dominant co-occurring temperature-CWB anomaly pairs identified in main text Figures 4-6, including warmer and drier summers across drought types and warmer and wetter springs. On-average warmer summers coincided with drier summers across drought types in 11% of warmer-drier models, resulting in average mortality risk increases of 10-22% depending on drought type. Warmer spring minimum temperatures coincided with reduced spring water deficits in 15% of warmer-wetter models, leading to increases of 13-15%. In both cases, combined effects tended to fall toward the upper range of the individual effect distributions, indicating reinforcing interactions when seasonal anomalies align (see Figure 6). For each violin plot, the left (darker) distribution summarizes responses across the nine most common species, whereas the right (lighter) distribution summarizes responses across all species. Annotations below each distribution report the mean change in predicted mortality risk and the number of contributing models (N), with bold text indicating results for the nine most common species. Boxplots show the median, 25th and 75th percentiles for model outputs across all 52 species; whiskers extend to the minimum and maximum values within 1.5 times the interquartile range from the quartiles.

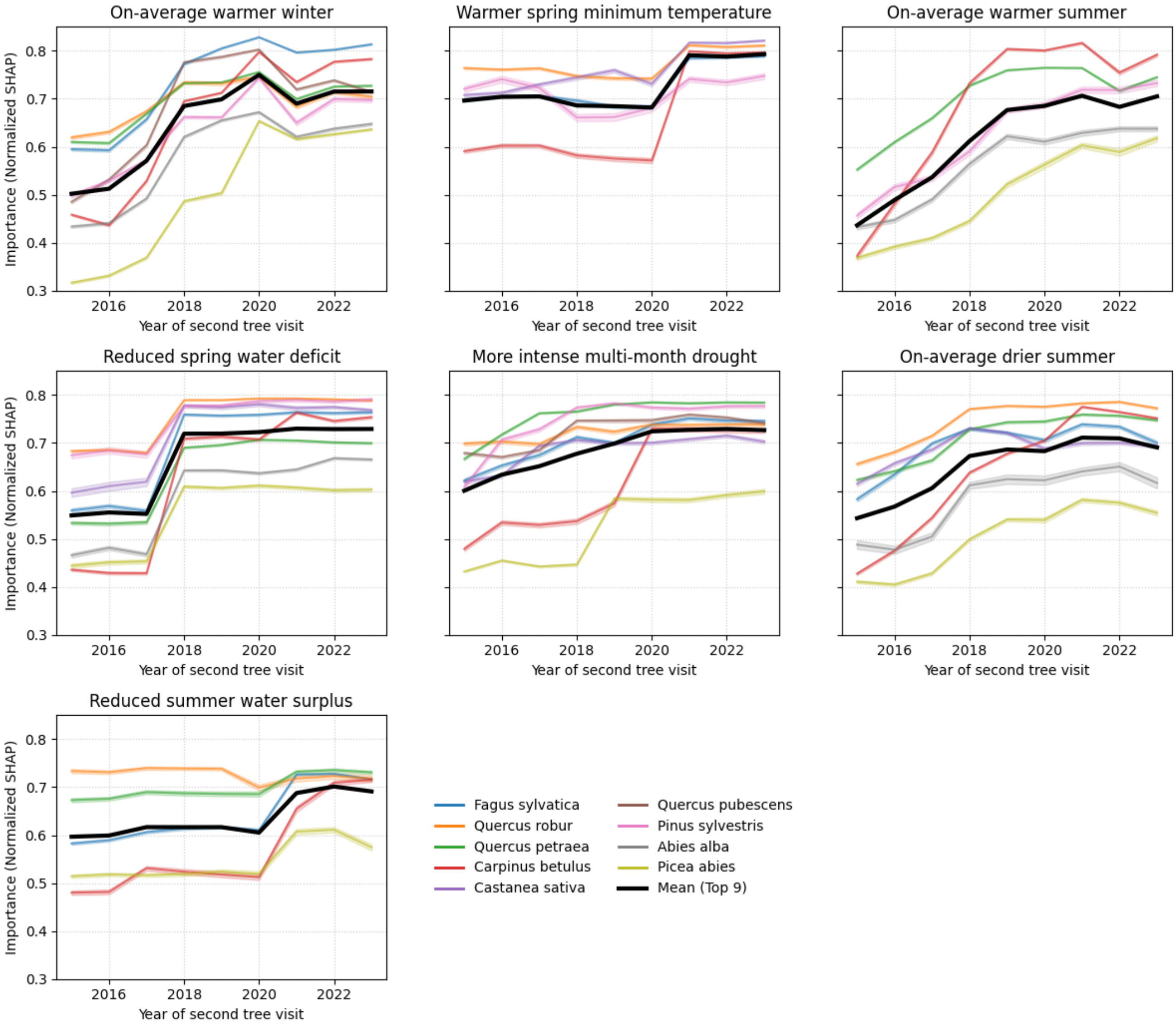

Figure S9. Temporal dynamics of different seasonal climate anomalies and their importance for predicting tree mortality risk across the nine most common species. Panels display the mean normalized SHAP values for seven distinct climate anomalies consistently linked to increased mortality. This includes temperature-based drivers (top row: warmer winters, spring minimums, and summers) and water-availability drivers (middle and bottom rows: e.g., intense droughts, drier summers, and reduced water surpluses). Colored lines represent species-specific means for trees recorded in each year of the second census, with shaded areas indicating 95% confidence intervals. The black line represents the mean importance across all nine species (weighted equally per species to control for sample size differences). Normalization of SHAP values within each model preserved the relative contribution of each feature while standardizing the scale, allowing direct comparison of feature importance over time. Species are color-coded as in the legend.

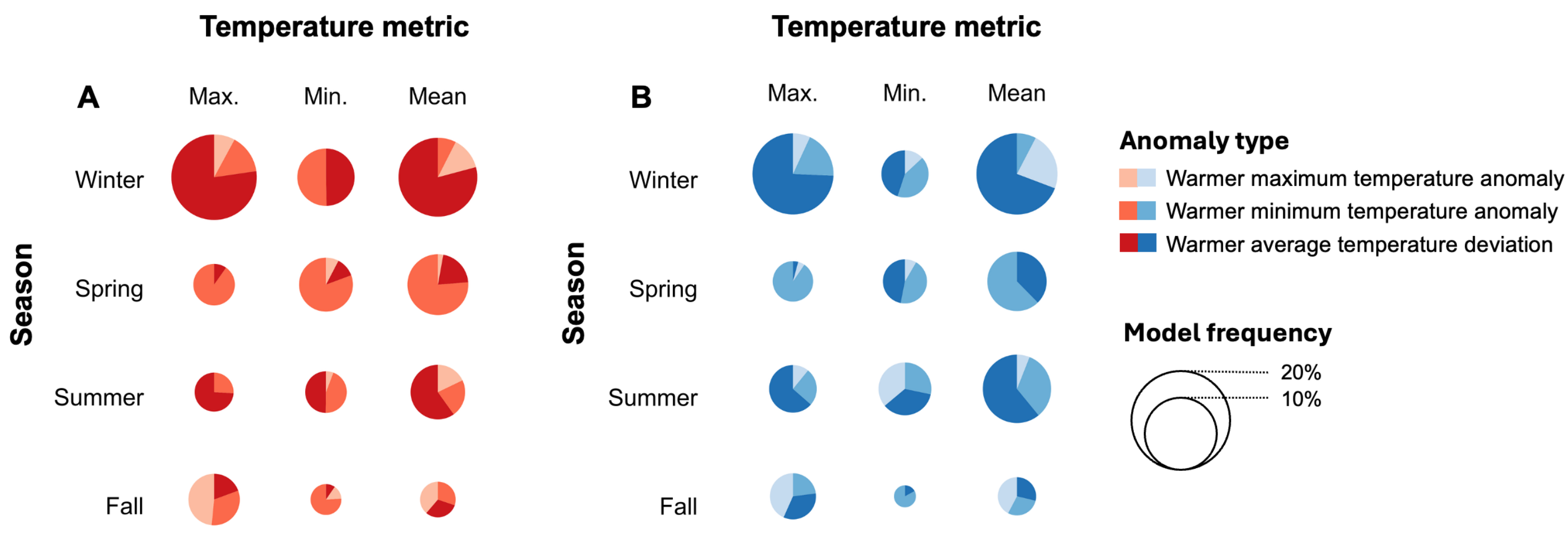

Figure S10. Equivalent to Figure 4 in the main text but with results for all 52 species analyzed in this study. The figure shows the distribution of temperature anomaly attributes from models associating increased mortality risk with warmer temperature anomalies. Models are split by whether the concurrent CWB feature associated mortality with drier (A, red shades, N = 1197 models) or wetter (B, blue shades, N = 516 models) anomalies of the climatic water balance. To derive these distributions, the selected temperature feature in each model was decomposed into its three attributes: season, temperature metric, and anomaly type (see Methods). The "Season" (rows) corresponds to the months over which anomalies were calculated (e.g., Winter refers to December-February), while the "Temperature metric" (columns) denotes whether anomalies were based on monthly maximum, minimum, or mean temperature. The "Anomaly type" (color shades) denotes whether a temperature feature quantified the maximum anomaly, minimum anomaly, or the average deviation during the seven years preceding the second tree census. The "Model frequency" (pie chart size) reflects the percentage of models within each panel that belong to a given season-temperature metric combination. Pie slices indicate the distribution of different anomaly types within each combination.

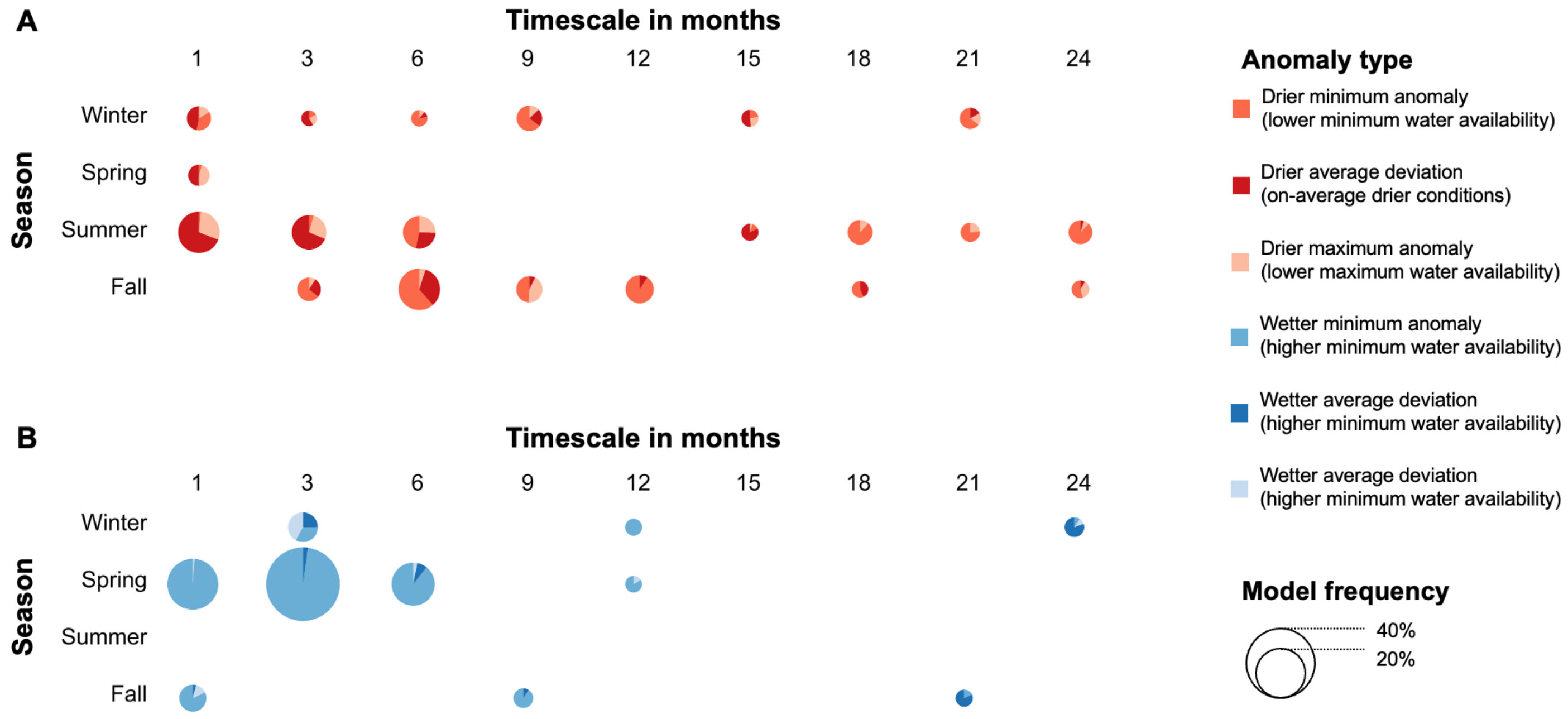

Figure S11. Equivalent to Figure 5 in the main text but with results for all 52 species analyzed in this study. The figure shows the distribution of the attributes of climatic water balance (CWB) features from models associating increased mortality risk with either drier (A, red shades, N = 1197 models) or wetter (B, blue shades, N = 516 models) anomalies. Only models in which warmer temperature anomalies concurrently increased mortality risk are shown; reported model frequencies therefore refer to the subsets of warmer-drier and warmer-wetter models, respectively. To derive these distributions, the CWB feature in each model was decomposed into its three attributes: season, integration timescale, and anomaly type (see Methods). The “Season” (rows) refers to the final month of the season (e.g., February for winter), while the “Timescale” (columns) denotes the number of months over which the CWB was integrated prior to that month (e.g., “Fall, 6” corresponds to June-November). The “Anomaly type” (color shades) denotes whether a CWB feature quantified the maximum anomaly, minimum anomaly, or average deviation during the seven years preceding the second tree census. The “Model frequency” (pie chart size) reflects the percentage of models within each panel that belong to a given season-timescale combination. Pie slices indicate the distribution of different anomaly types within each combination.

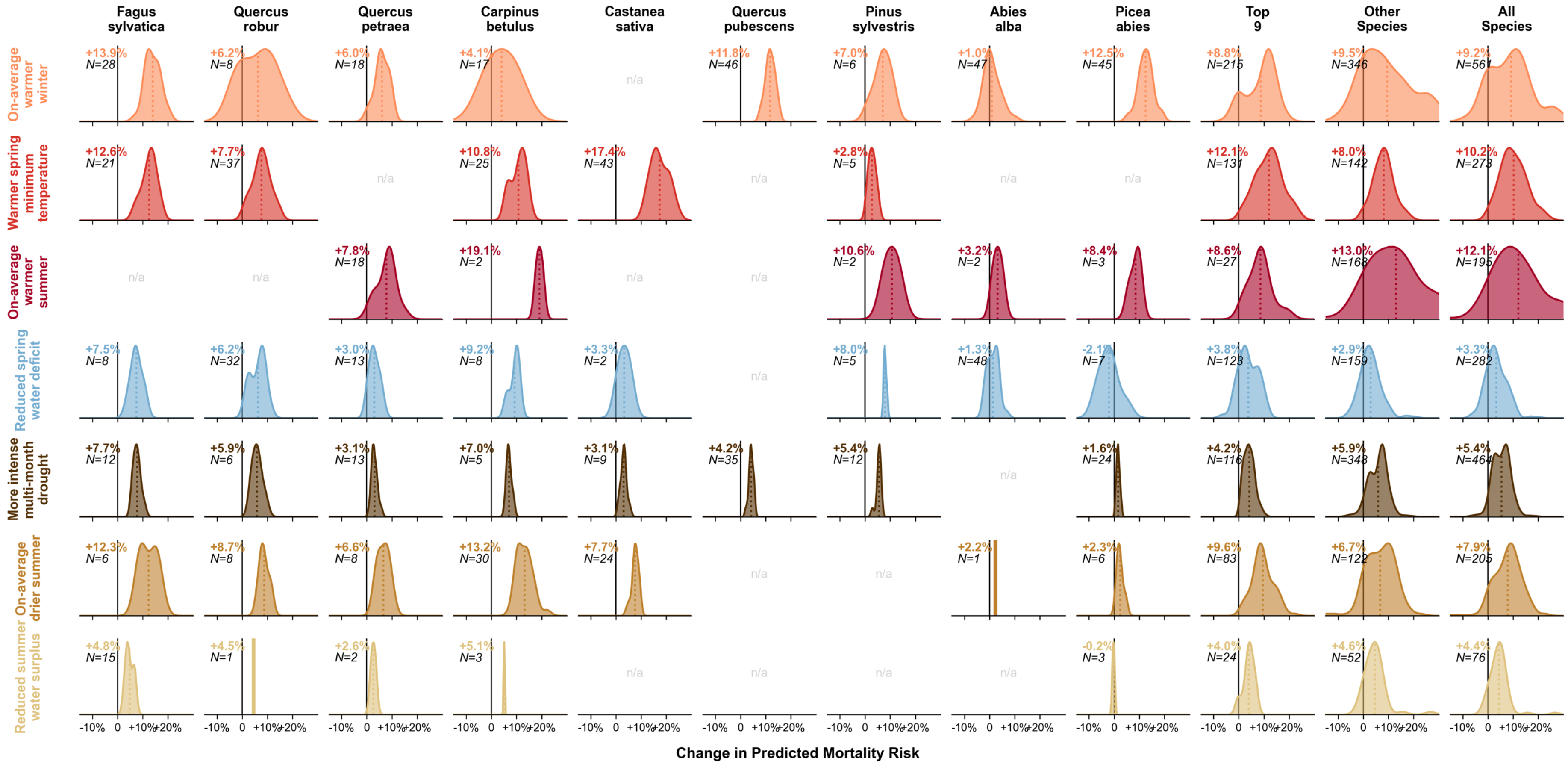

Figure S12. Species-specific distributions of predicted additional tree mortality risk across dominant seasonal climate anomalies. Density plots show the distribution of changes in predicted tree mortality risk for individual species, quantified by comparing observed seasonal climate conditions to alternatives in which the respective seasonal anomaly was shifted by one standard deviation away from the modelled mortality response (see Methods). Rows correspond to the main temperature and climatic water balance anomaly groups identified in main text Figures 4 and 5, including on-average warmer winters, warmer spring minimum temperatures, on-average warmer summers, reduced spring water deficits, more intense multi-month droughts, on-average drier summers, and reduced summer water surplus. Columns show results for each of the nine most common species, as well as aggregated responses across the nine most common species ("Top 9"), all remaining species ("Other species"), and all species combined. Annotated values report the mean change in predicted mortality risk and the number of contributing models for each species-anomaly combination, with dashed vertical lines indicating the mean of each distribution. For mean predicted additional mortality risk across all species, see Table S8.

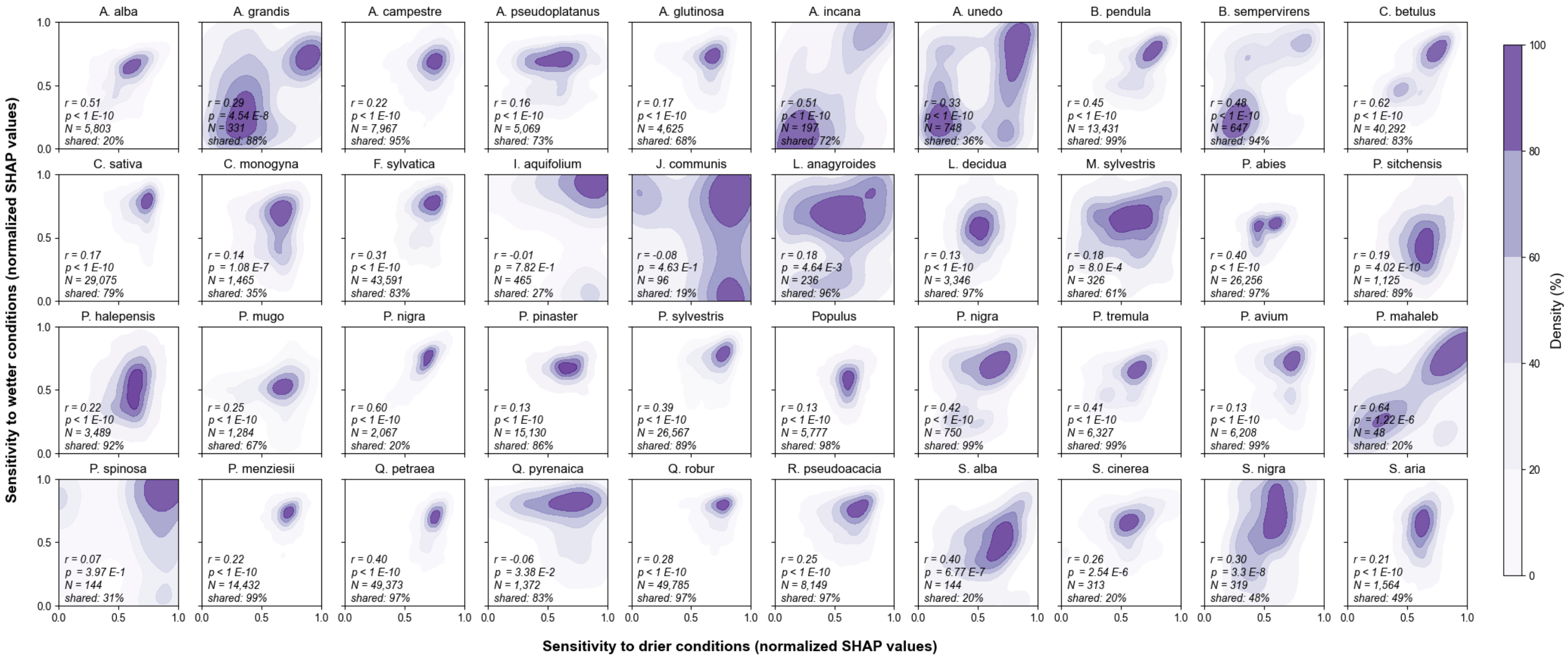

Figure S13. Equivalent to Figure 8 in the main text but with results from all 52 species analyzed in this study. Bivariate density plots showing tree-level sensitivity wetter spring and drier summer conditions, based on normalized SHAP values of CWB features (see Methods). Color intensity indicates the density of trees with similar sensitivity. Correlation coefficients (two-sided Spearman's r) quantify how strongly trees sensitive to wet anomalies were also sensitive to dry anomalies. The shared percentage indicates the proportion of trees out of all trees (for a given species) occurring in test sets of both the wetter- and drier-response model subsets. N indicates the corresponding count of shared trees used to estimate the density and correlation. Only results for species are shown that had at least one model linking increased mortality to drier and one model linking increased mortality to wetter conditions.

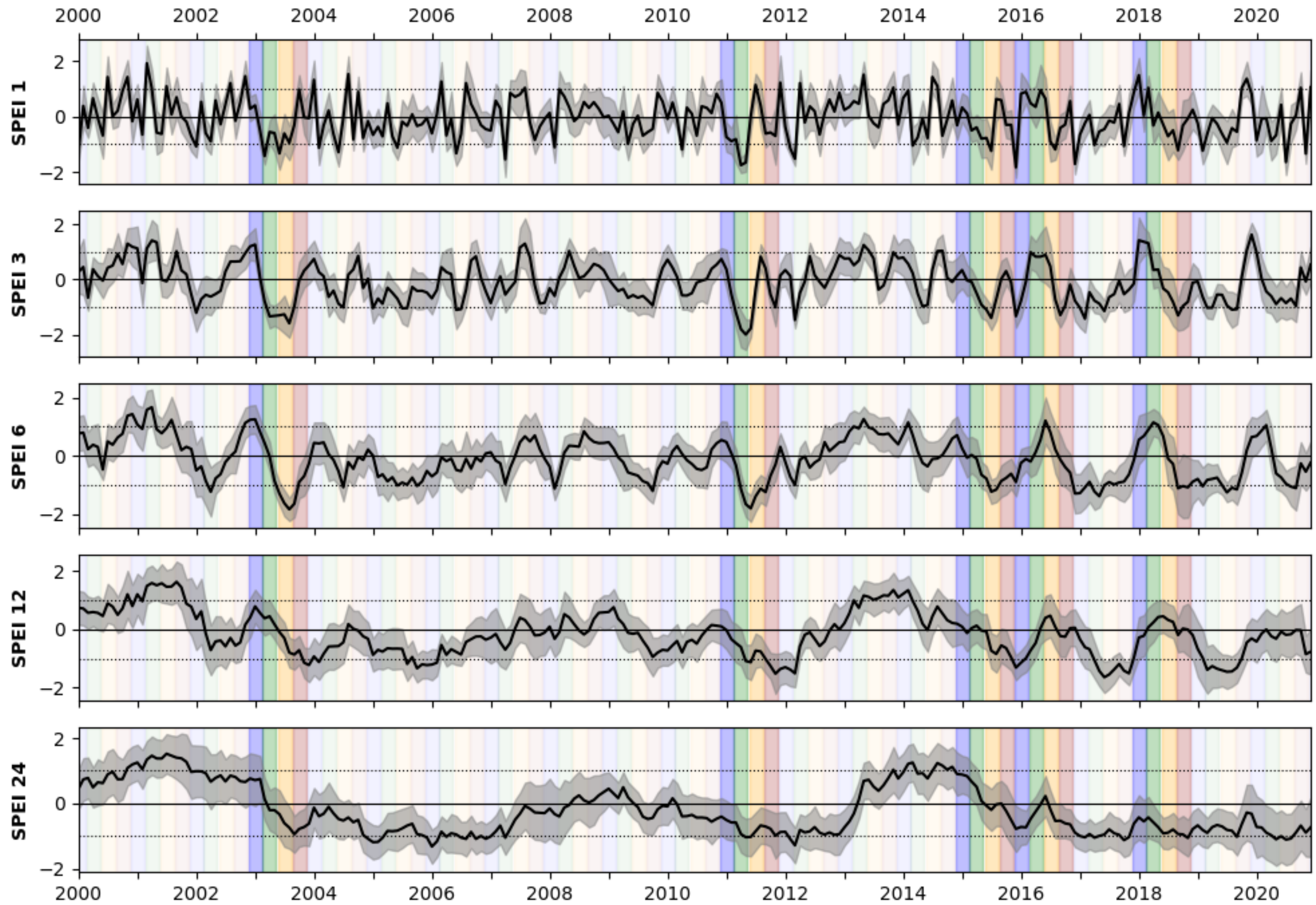

Figure S14. Seasonal progression of drought intensity across different SPEI accumulation windows from 2000 to 2020. Black lines show the mean standardized precipitation-evapotranspiration index (SPEI) at timescales of 1, 3, 6, 12, and 24 months, with shaded ribbons indicating ±1 standard deviation across all NFI sites. Black dotted lines mark SPEI thresholds of ±1, beyond which conditions are considered unusually dry or wet. Seasonal background colors indicate transitions in the hydrological year (Winter: blue, Spring: green, Summer: orange, Autumn: brown; years with major droughts are highlighted with stronger background color saturation). Notable drought events in 2003, 2011, 2015, 2016, and 2018 are clearly visible across timescales, particularly in SPEI3 and SPEI6, which integrate water balance anomalies over three- and six-month windows and emphasize transitions between seasons. The 2003 and 2016 droughts were preceded by already dry winter and spring conditions that continued into a dry summer but relatively wet fall. The 2011 and 2015 droughts occurred primarily during a dry spring followed by a wet summer. The 2018 drought was distinct in being preceded by an exceptionally wet spring, followed by an abrupt transition to severe dryness in summer - a wet-to-dry seasonal swing not observed in other recent events. A similar swing pattern emerged in 2016, but winter and spring were already relatively dry due to legacy effects of the 2015 drought, and the onset of dryness occurred later in summer. All anomalies are relative to the 1960-2020 reference period.

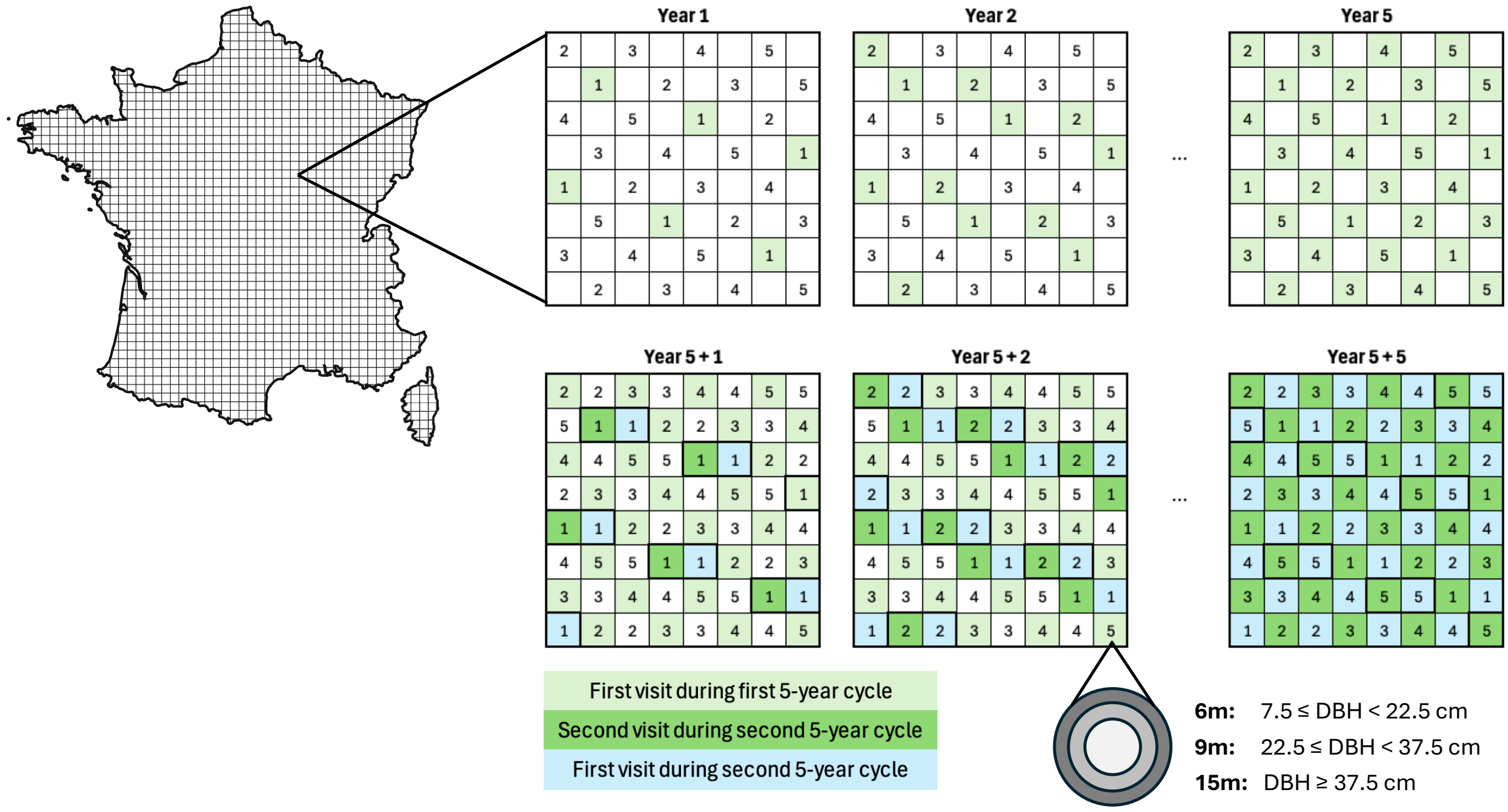

Figure S15: French National Forest Inventory sampling and revisit design. The French NFI uses a spatially distributed annual grid in which one fifth of sampling units is visited each year, providing national coverage over each five-year cycle. The map illustrates the national grid, and the panels show how annual samples accumulate during the first cycle (Years 1-5) and how previously sampled units are revisited during the second cycle (Years 5+1 to 5+5). Light green cells indicate first visits during the first five-year cycle, dark green cells indicate second visits during the second five-year cycle, and blue cells indicate first visits introduced during the second cycle. This rolling design allows tree status to be assessed over five-year revisit intervals while maintaining consistent national coverage across inventory windows. Trees are recorded in concentric plots with radii of 6, 9, and 15 m according to DBH classes of 7.5-22.5 cm, 22.5-37.5 cm, and >37.5 cm, respectively. Adapted with permission from the Institut national de l'information géographique et forestière (IGN).

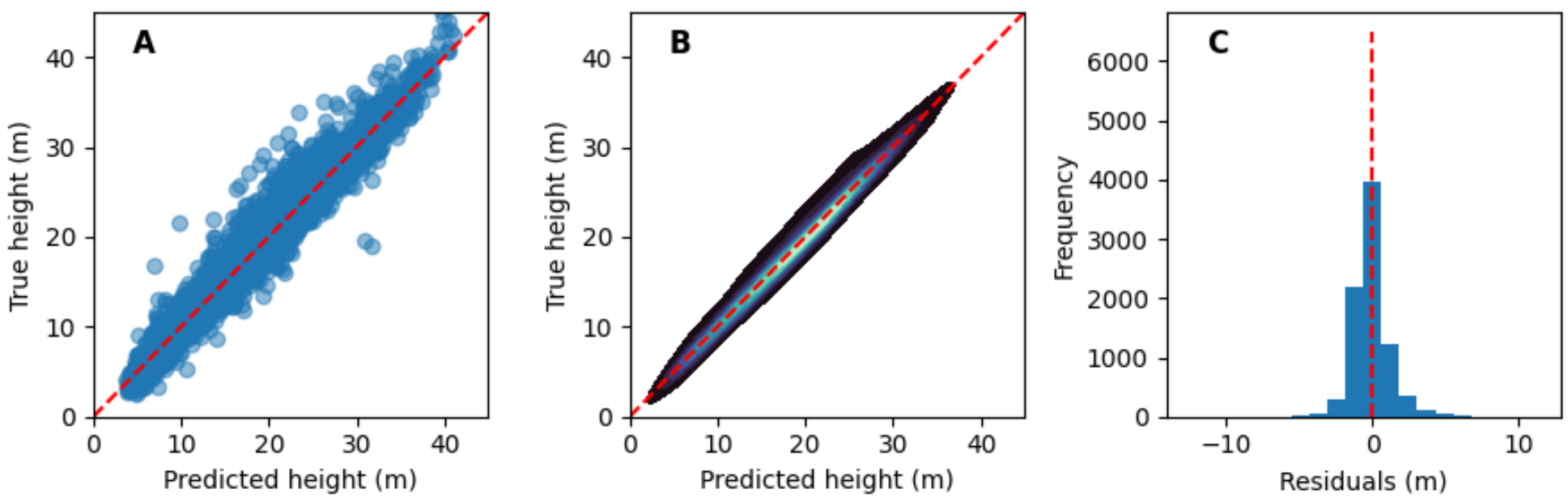

Figure S16. Results of random forest regression to estimate tree height. True versus predicted height as scatter (A) and density plots (B) with red diagonal dashed lines showing the one-to-one line. Distribution of residuals with the red vertical dashed line showing at residual equal zero (C).

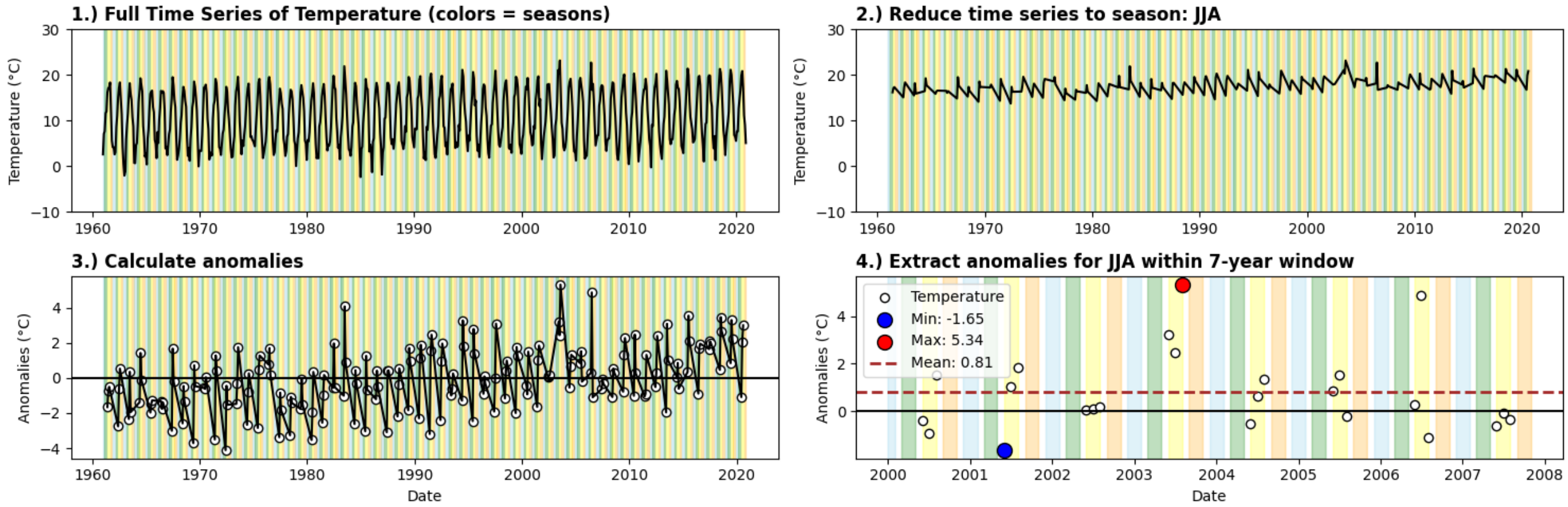

Figure S17. Example for calculating temperature anomalies for a specific season. (1) The full temperature time series (1960-2020) is displayed with seasons indicated by background colors. (2) The time series is reduced to the summer season (JJA). (3) Temperature anomalies are calculated by subtracting the long-term seasonal mean from observed values. (4) Anomalies for JJA between two visits are extracted, showing minimum, maximum, and mean anomaly values, with points representing individual years and the red dashed line indicating the mean of all anomalies.

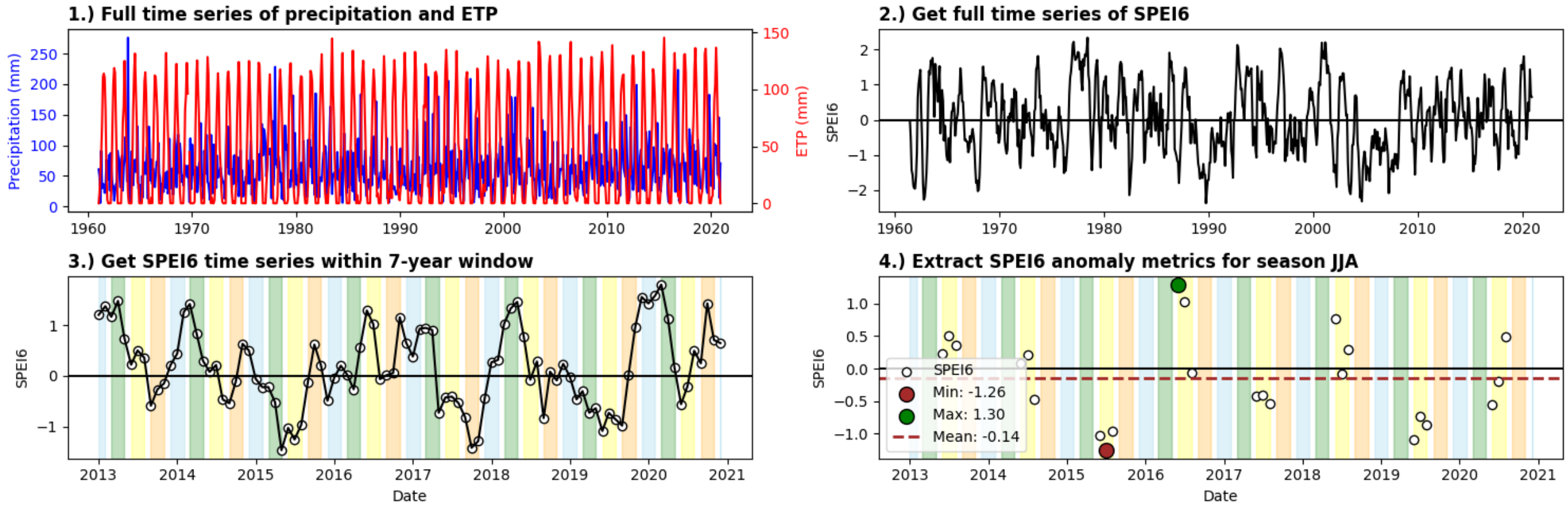

Figure S18. Example for calculating SPEI6 anomalies (standardized Precipitation-Evapotranspiration Index on a 6-month integration timescale). (1) Full time series of precipitation (blue) and evapotranspiration (ETP, red) from 1960 to 2020. (2) Derived SPEI6 time series calculated using precipitation and ETP data. (3) SPEI6 values between two visits, with seasons highlighted by background colors. (4) Extracted SPEI6 anomalies for the summer season (JJA) between two visits, showing minimum, maximum, and mean values, with the red dashed line indicating the mean of all anomalies.

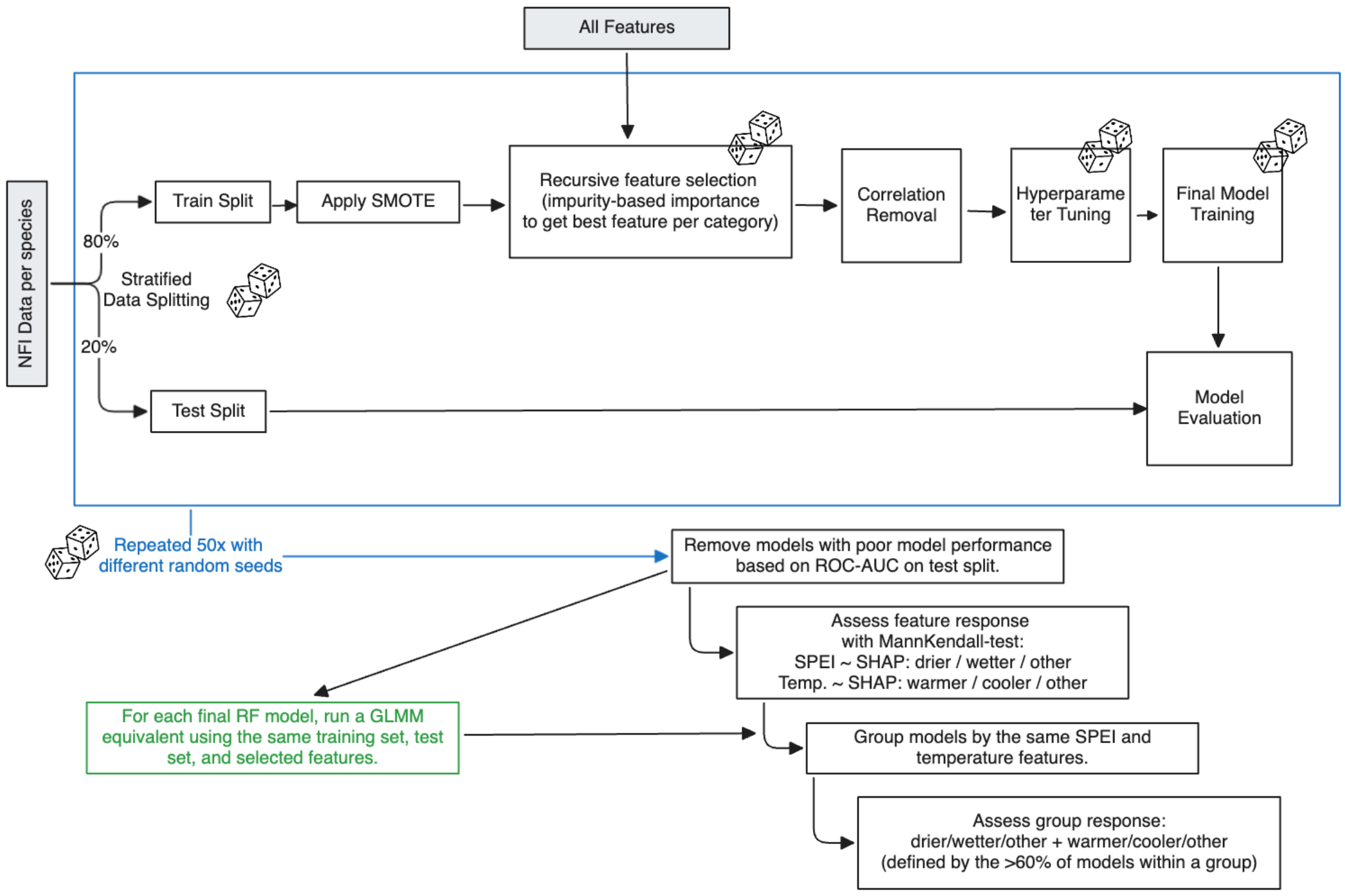

Figure S19. Explorative random forest algorithm to detect the mortality response to multiple feature combinations. Blue colors and dice pictograms show where randomized procedures are involved that are altered across the 50 model replicates per species. “SMOTE” stands for Synthetic Minority Oversampling Technique. The individual steps are explained in detail in the Methods section of the main text.

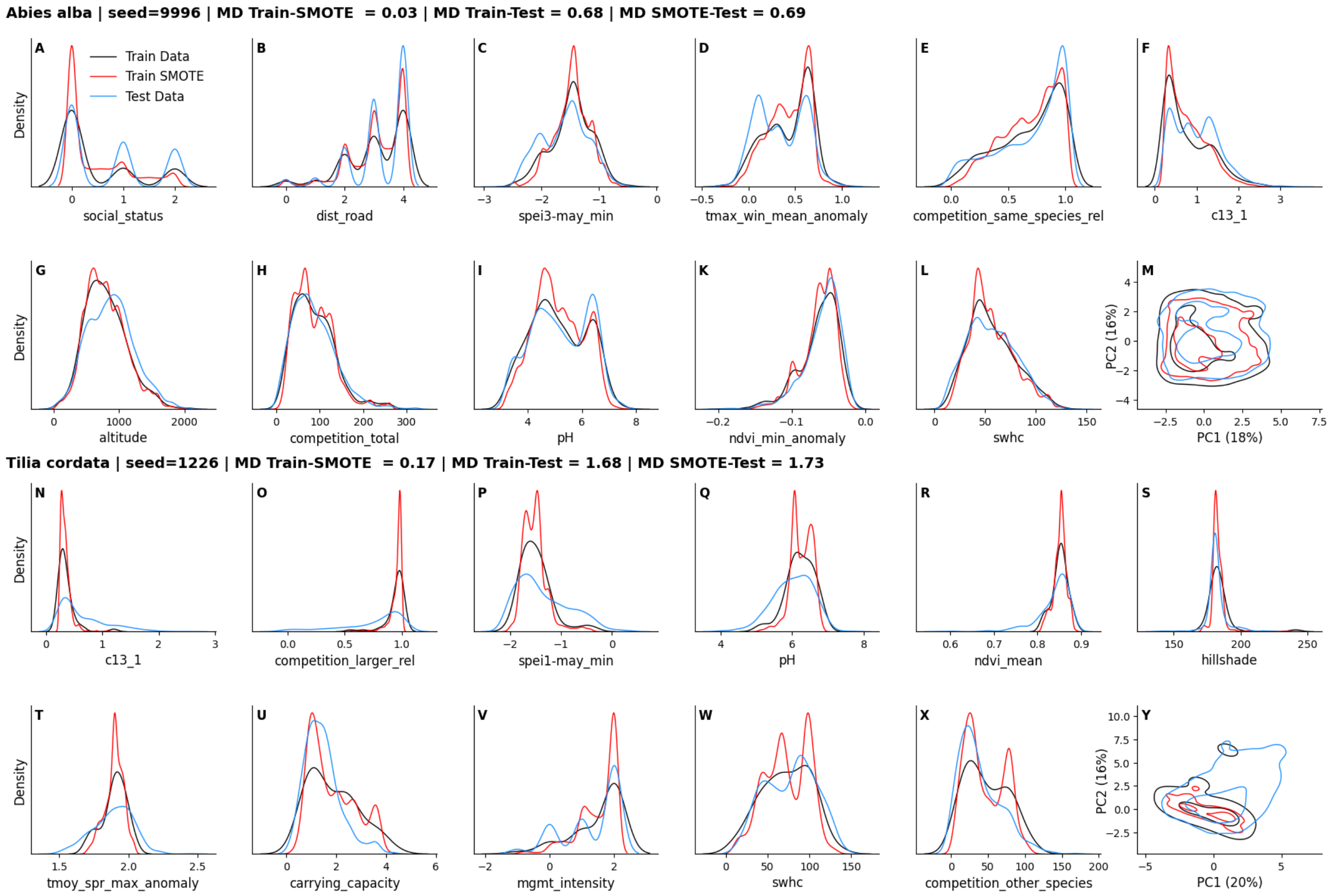

Figure S20. Distributional similarity of original train, SMOTE-augmented train, and test set feature spaces in two contrasting cases. Panels A-L and N-X show kernel density estimates (KDEs) for each model predictor, comparing the distributions of the mortality class in the original training data (black), SMOTE-augmented training data (red), and the test set (blue). Panels M and Y display PCA density contours (PC1 vs. PC2) based on all standardized predictors, illustrating multivariate overlap. In the top example (Abies alba), all three distributions are closely aligned, with low Mahalanobis distances (MD) between train, SMOTE, and test sets, indicating high structural similarity. In contrast, the bottom example (Alnus incana) shows low MD between the SMOTE and original mortality samples, but both diverge more strongly from the test set. Despite differing levels of agreement with the test data, both examples illustrate that SMOTE generates synthetic samples that remain tightly anchored to the structure of the original training distribution. This confirms that SMOTE preserves the ecological plausibility of observed mortality patterns without introducing unrealistic variability. The data shown correspond to the final random forest models obtained after recursive feature selection, using a SMOTE k = 5 and resampling only the minority class (mortality cases) for the respective species and seed.

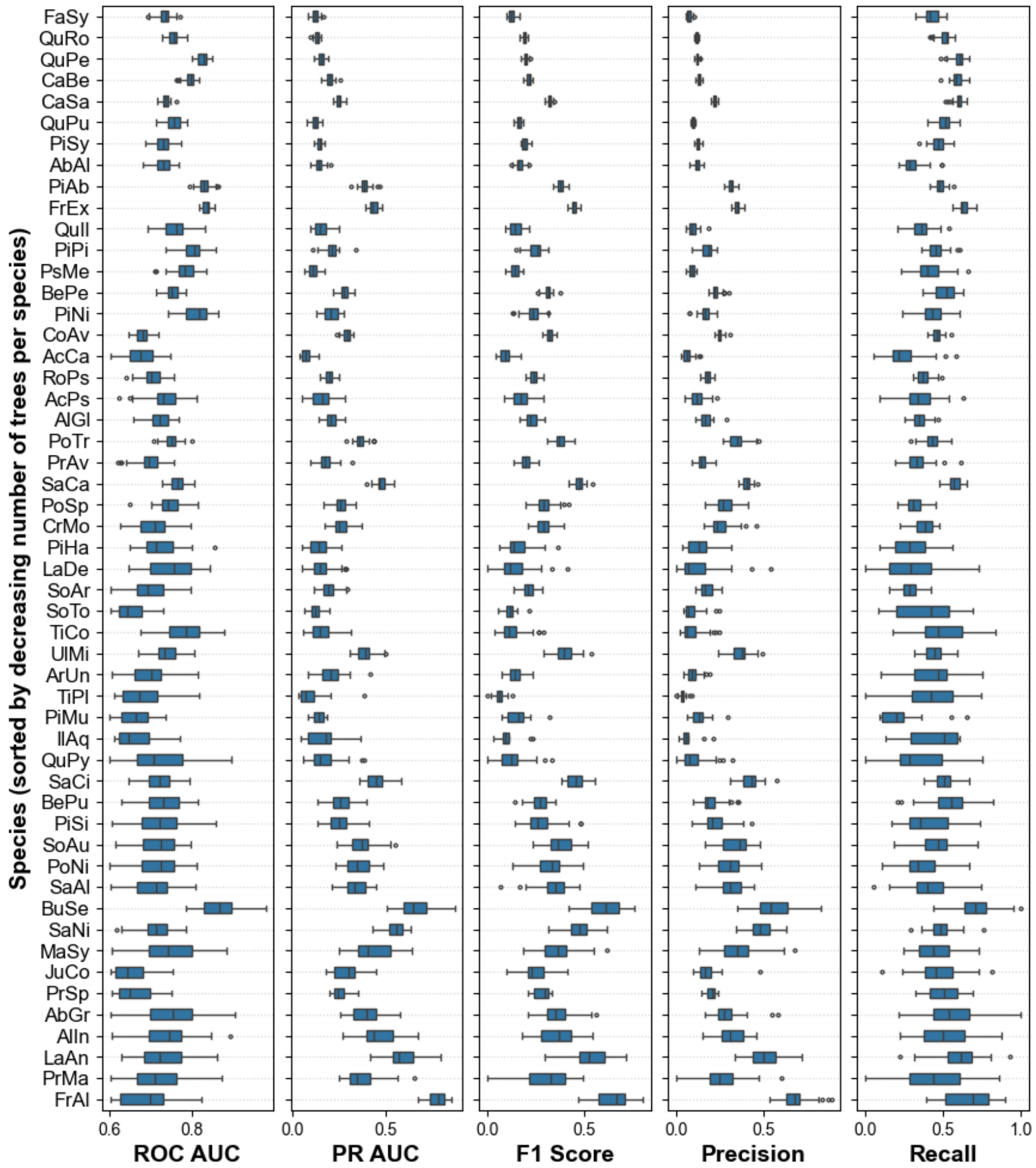

Figure S21. Mahalanobis distances between training, SMOTE-augmented, and test feature spaces across all species. Each boxplot shows the distribution of Mahalanobis distances for a given species across 50 random seeds, comparing the predictor space between: original training vs. SMOTE-augmented data (left column), original training vs. test data (middle), and SMOTE-augmented vs. test data (right). Distances were computed in the standardized predictor space used by the final random forest models after recursive feature selection. Across species, SMOTE samples consistently remain close to the original training data, demonstrating structural similarity and suggesting that the synthetic data respect the geometry of the observed mortality space. In contrast, distances between training or SMOTE data and the test set are generally larger, reflecting greater spatio-termporal variability. All results are based on models using a SMOTE k = 5, which defines the number of nearest neighbors used in the algorithm to generate synthetic samples, applied exclusively to the minority class for each species and seed.

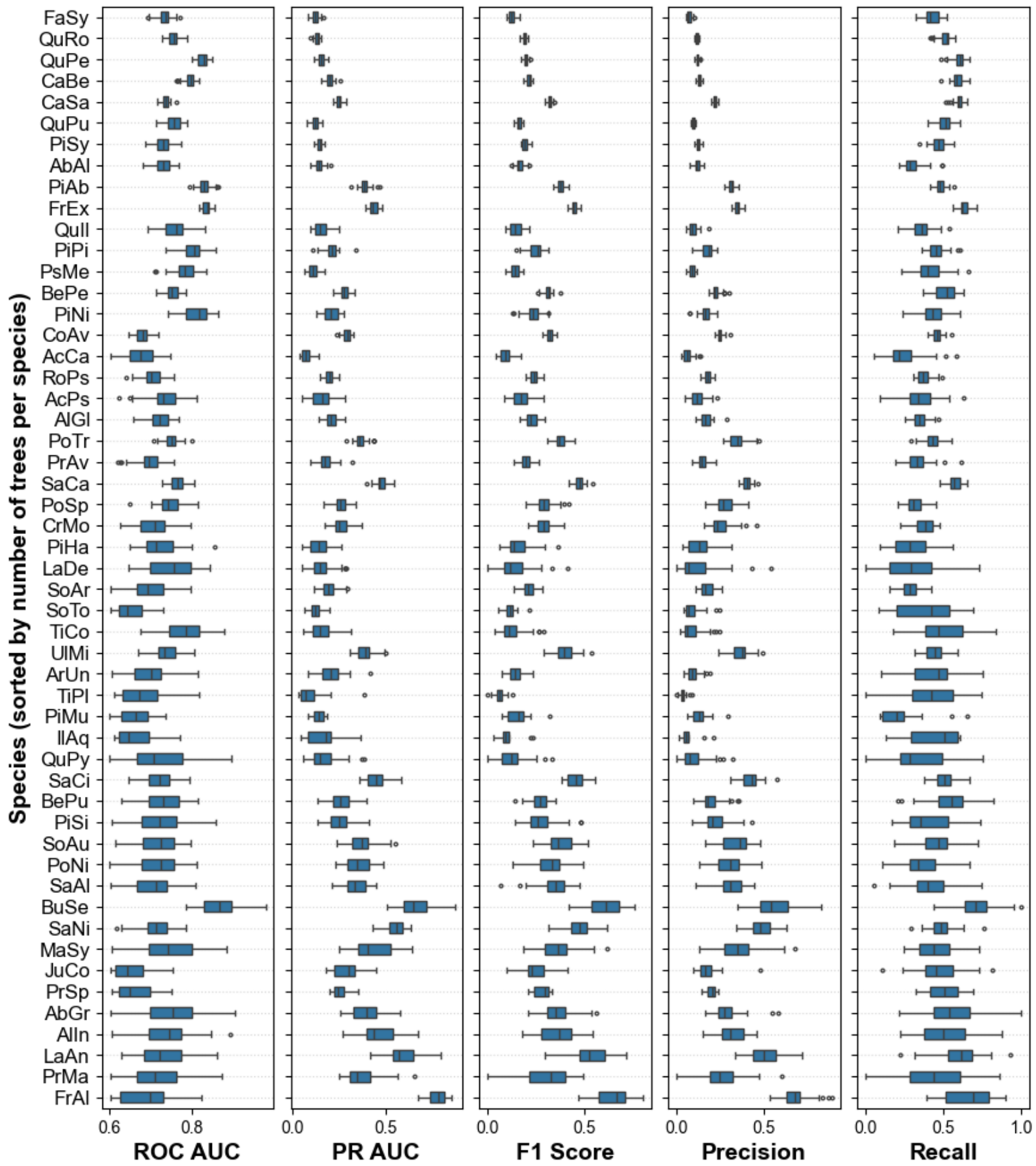

Figure S22. Predictive performance on the test dataset across species and evaluation metrics. Each of the five subplots corresponds to an evaluation metric: ROC-AUC, PR-AUC, F1-Score, Precision, and Recall. For each species, the boxplot summarizes the performance across 50 model replicates evaluated on the test dataset. Only models with ROC-AUC > 0.6 are included, thus ROC-AUC values do not fall below 0.6. Species are ordered by decreasing occurrence (number of trees in the dataset) and names are abbreviated to initials (e.g., Fagus sylvatica as FaSy; see Table S1). In general, species with larger sample sizes exhibit more consistent performance across replicate runs.

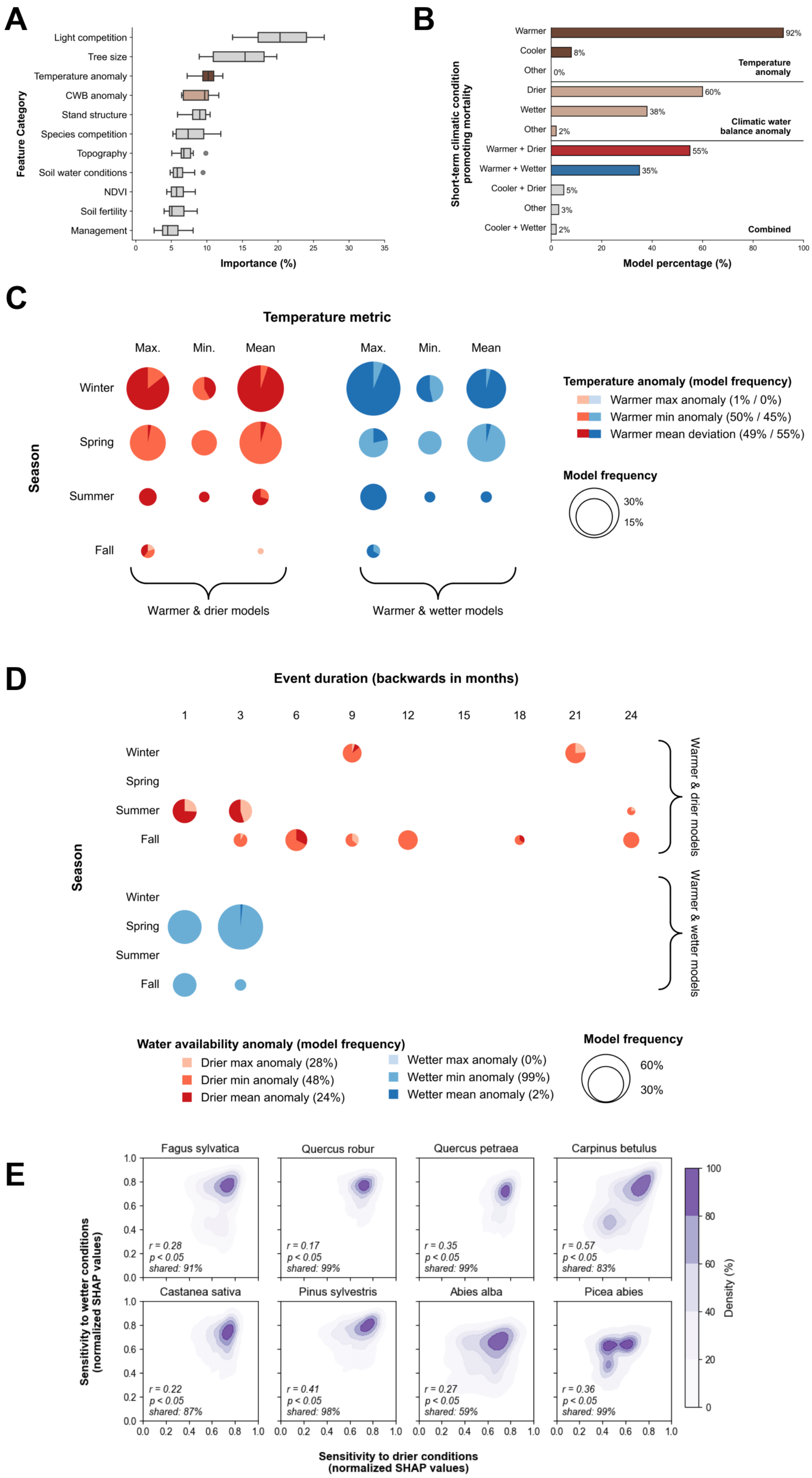

Figure S23. Model results when retaining four features per category before final feature selection. Panels A-E replicate Figures 3, 4, 5, and 8 in the main text but when retaining four features per category, instead of the default two, before selecting the single best feature per category.

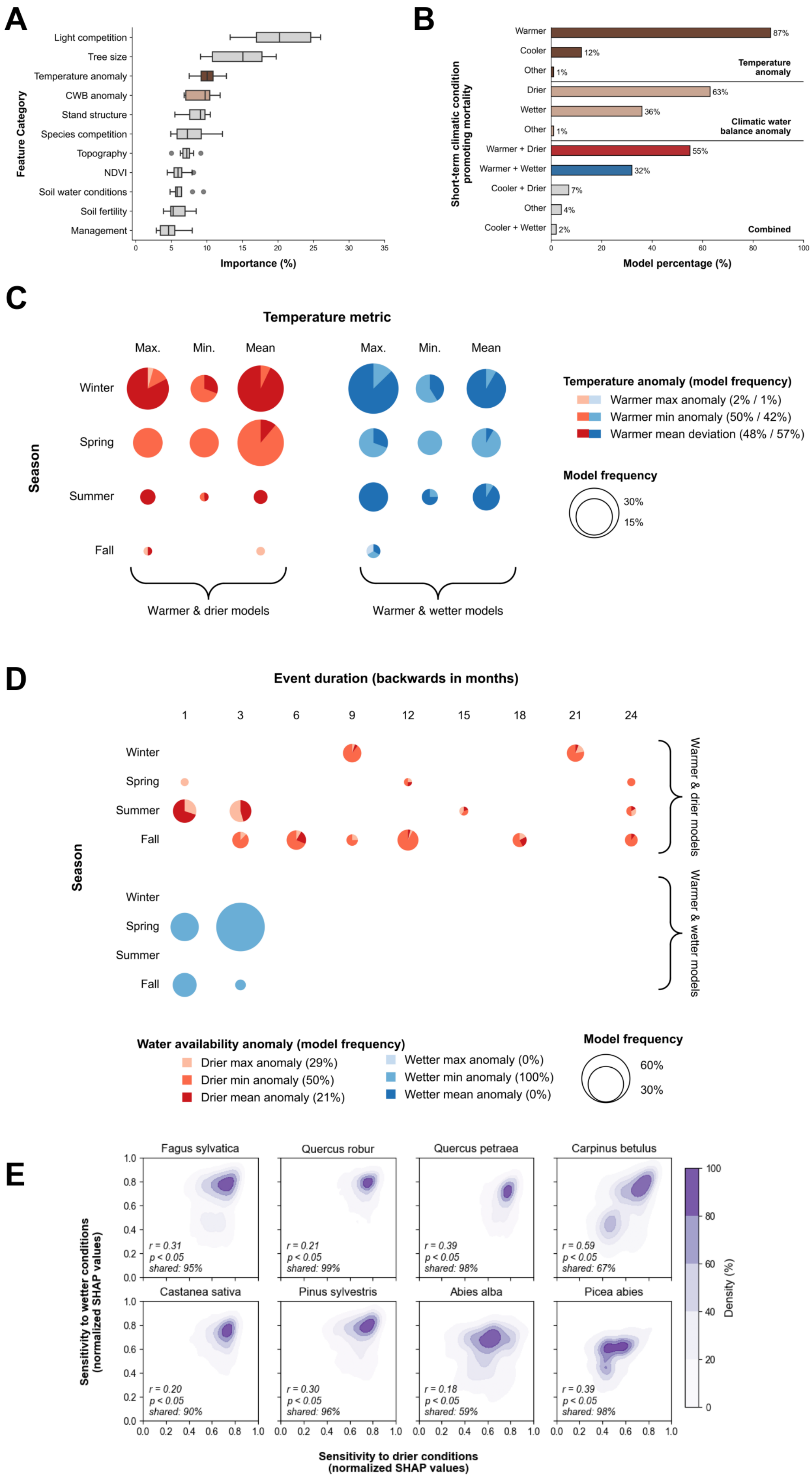

Figure S24. Model results when retaining six features per category before final feature selection. Panels A-E replicate Figures 3, 4, 5, and 8 in the main text but when retaining six features per category, instead of the default two, before selecting the single best feature per category.

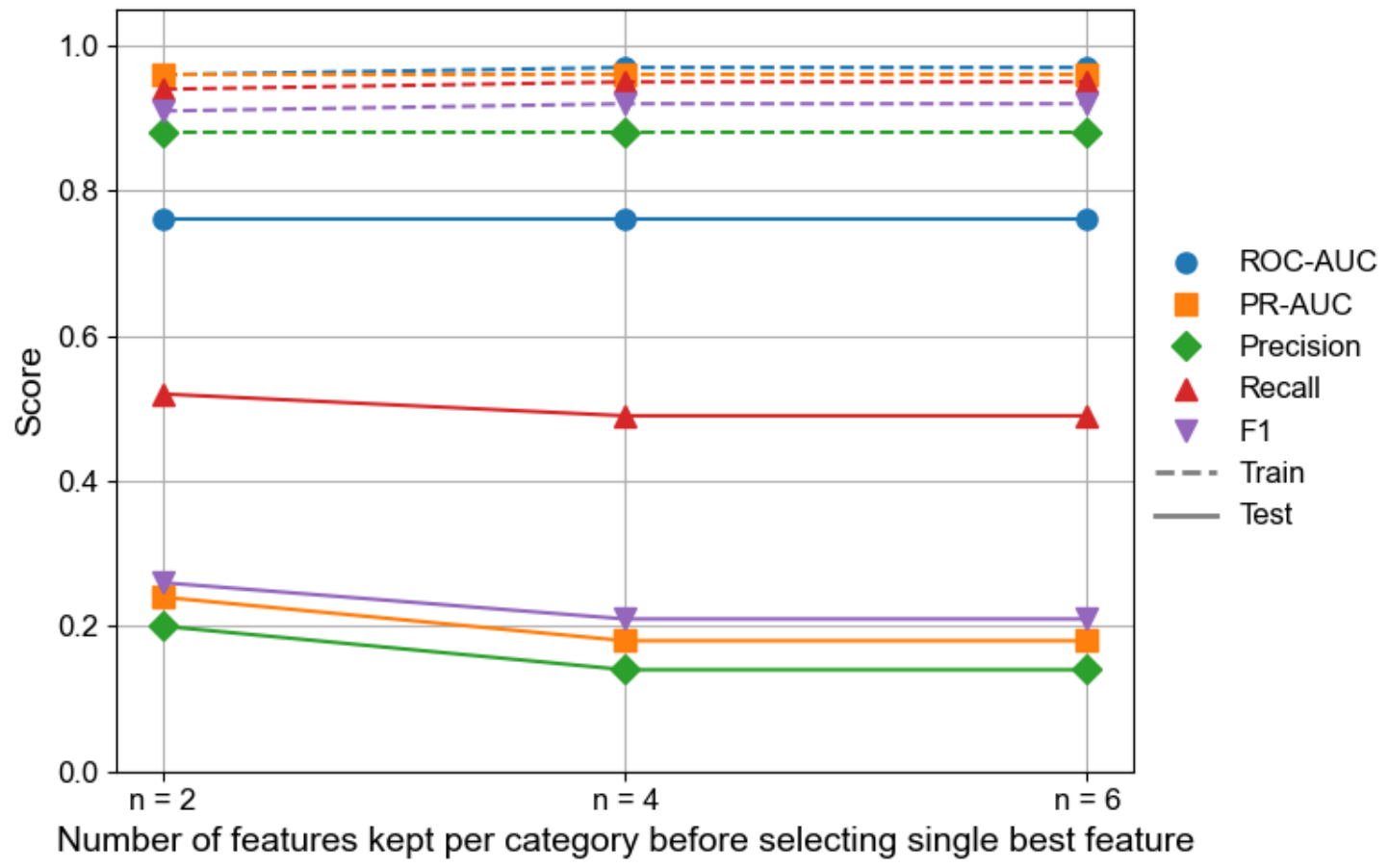

Figure S25. Retaining fewer features per category during recursive feature selection slightly improves model performance. Train and test performance across settings that varied the number of features retained per feature category prior to selecting the single best feature. We compared three thresholds (n = 2, 4, 6), evaluating five classification metrics: ROC-AUC, PR-AUC, precision, recall, and F1 score. Colored markers indicate individual metrics, with dashed and solid lines representing training and test performance, respectively. Metrics are averaged across 50 replicate random forest runs for the nine most common species. While performance is broadly consistent across settings, n = 2 yields slightly higher scores across all metrics, suggesting that retaining fewer features before final selection may help avoid overfitting and improve generalization.

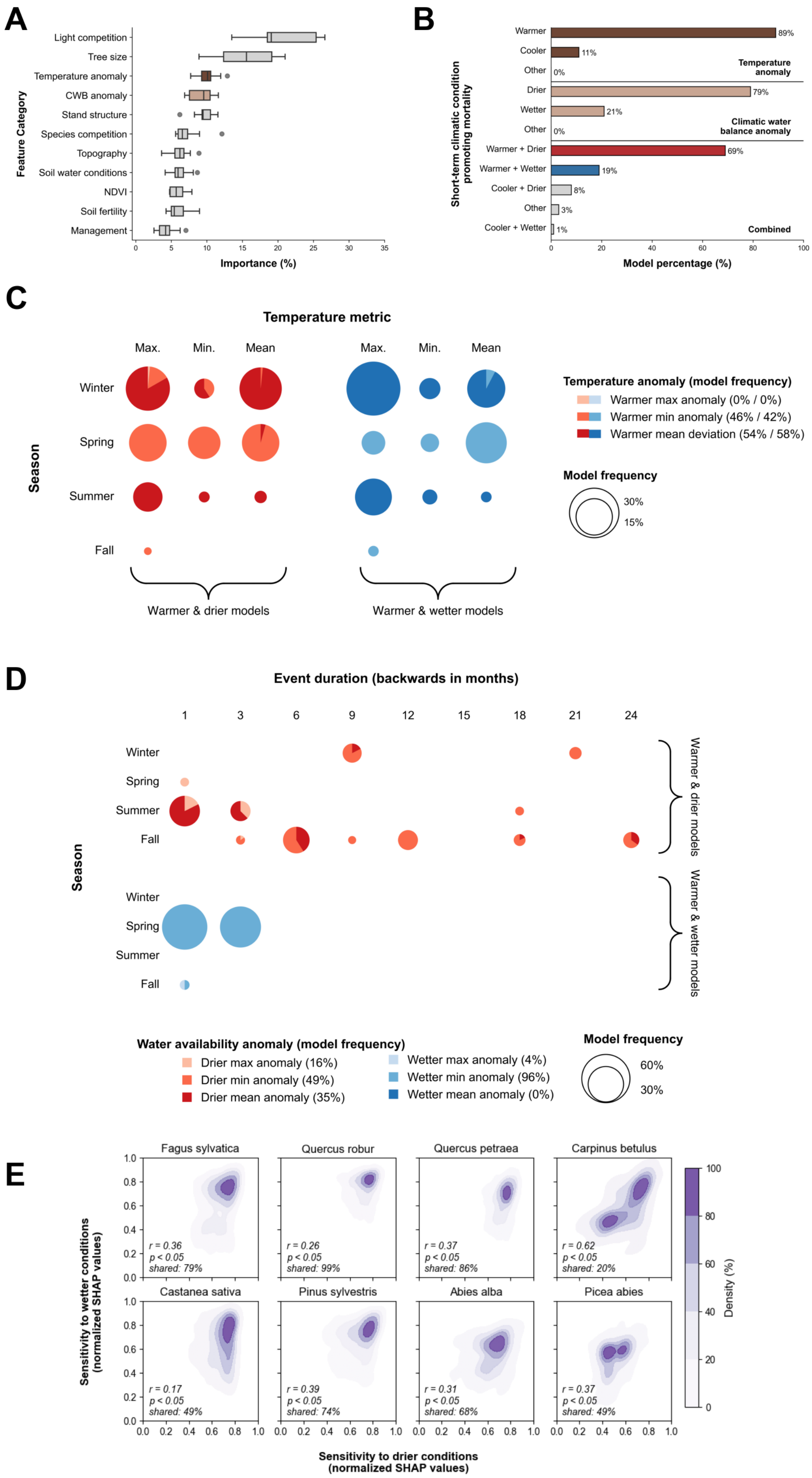

Figure S26. Model results using SMOTE with k = 1 nearest neighbor. Panels A-E replicate Figures 3, 4, 5, and 8 in the main text but using SMOTE with k = 1, generating synthetic samples of dead trees based on only the closest neighbor in feature space.

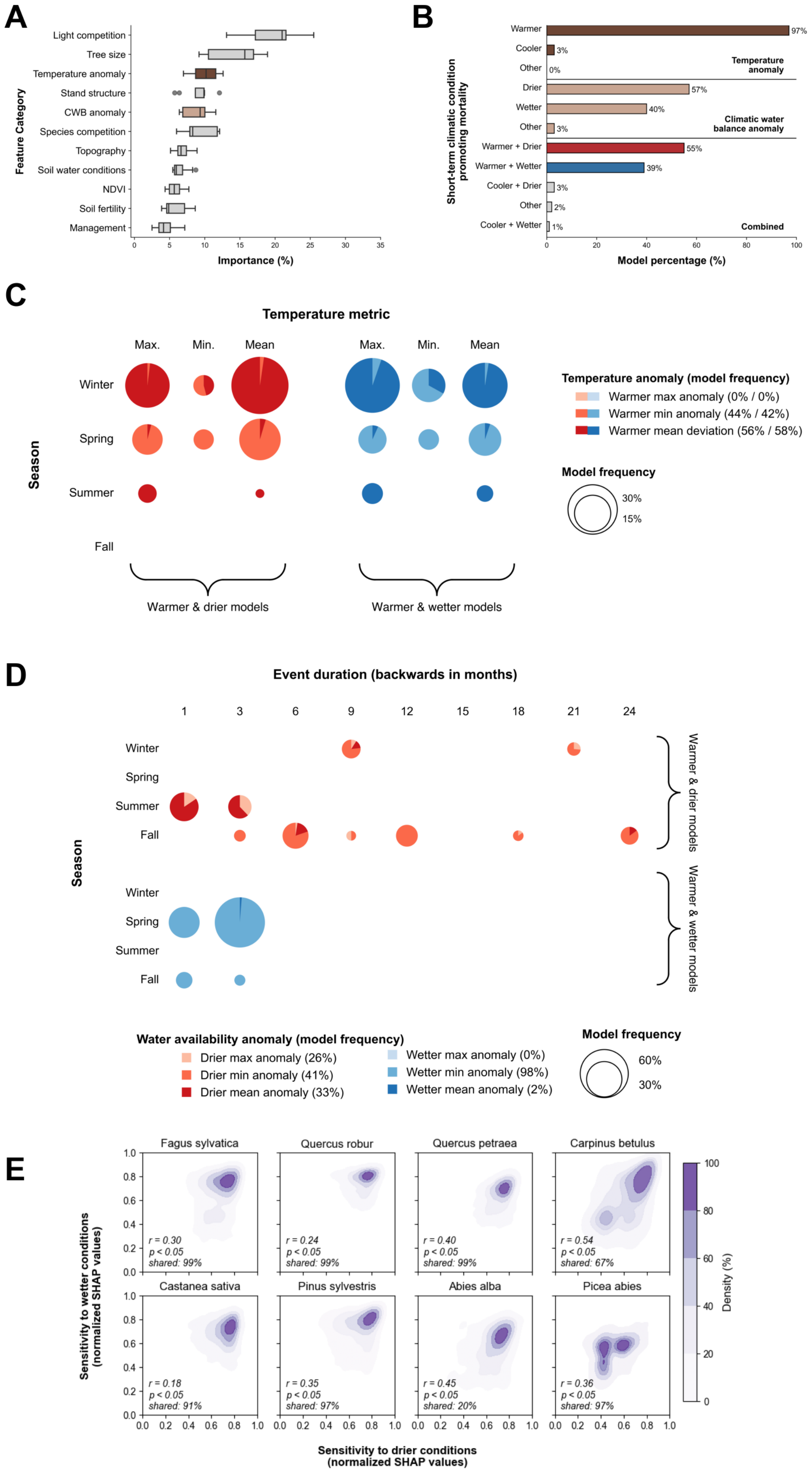

Figure S27. Model results using SMOTE with k = 15 nearest neighbors. Panels A-E replicate Figures 3, 4, 5, and 8 in the main text but using SMOTE with k = 15, generating synthetic samples of dead trees by interpolating across a broader neighborhood in feature space.

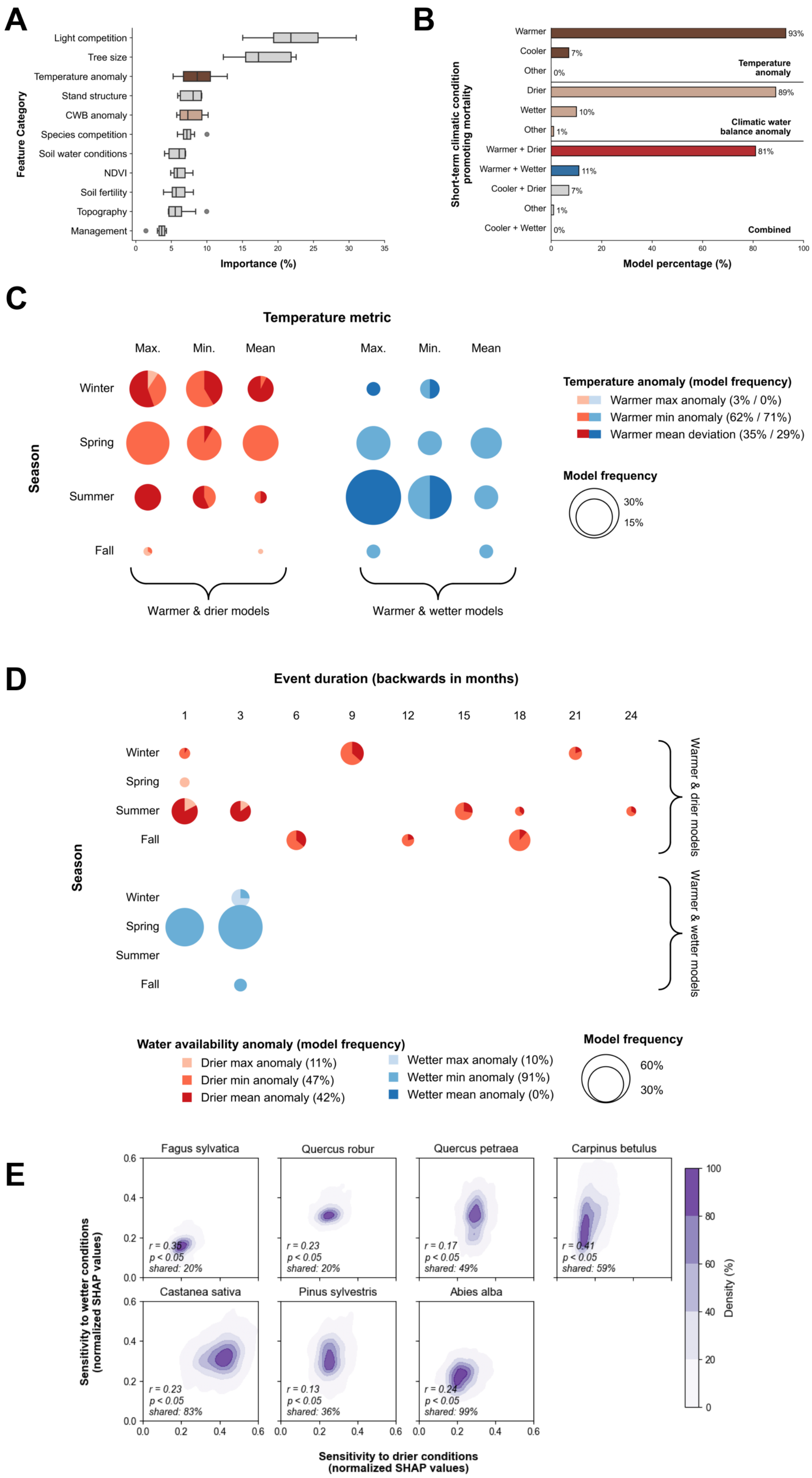

Figure S28. Main results for not using SMOTE entirely. Panels A-E replicate Figures 3, 4, 5, and 8 in the main text but without applying any oversampling via SMOTE.

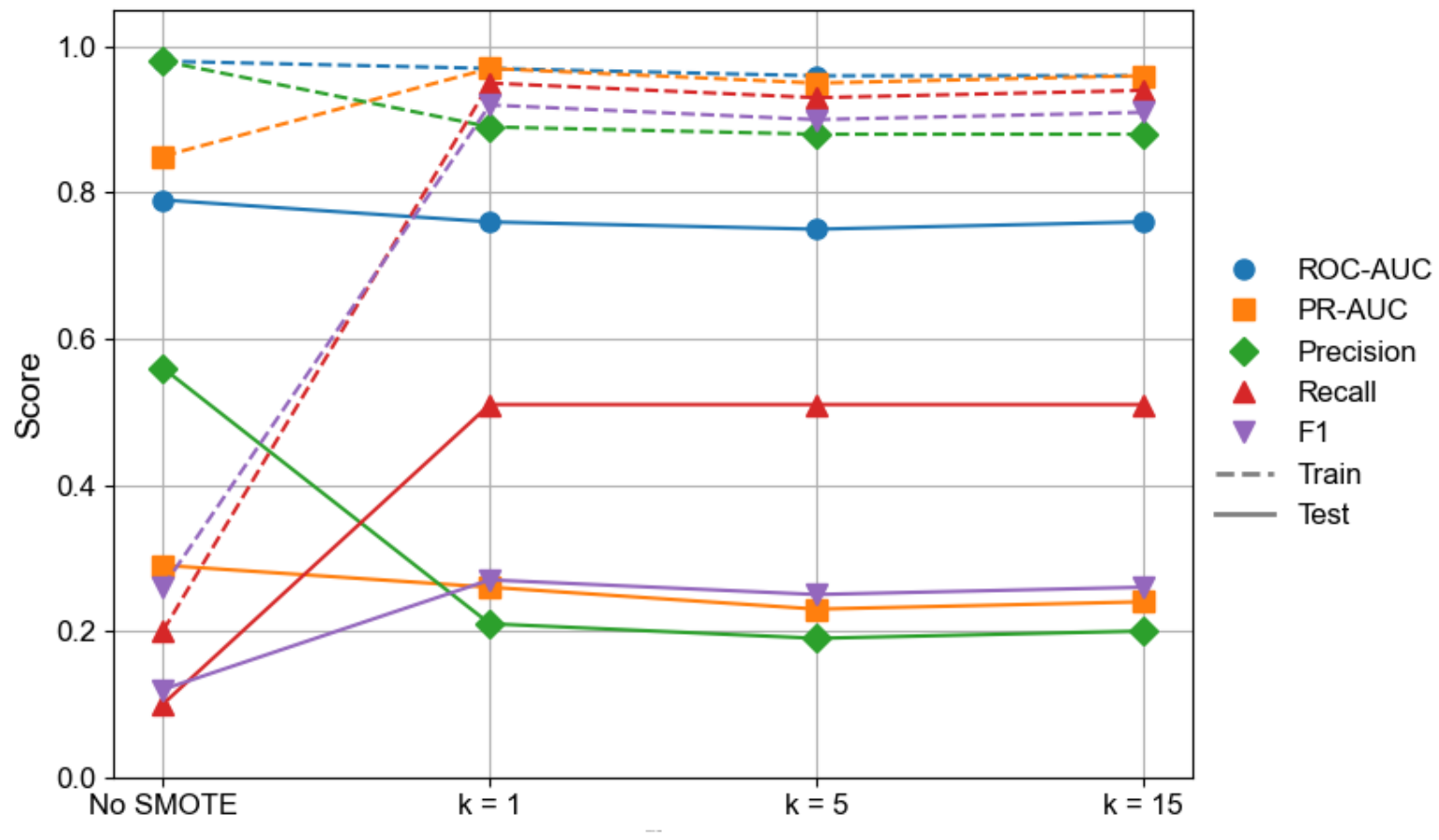

Figure S29. SMOTE improves mortality-event detection while preserving rank-based model performance. Train and test performance across SMOTE settings used for random forest classification of tree mortality. Model performance was evaluated using ROC-AUC, PR-AUC, precision, recall, and F1 score. Colors and symbols indicate metrics, while dashed and solid lines distinguish training and test performance, respectively. The recursive feature selection and final model training routine was repeated with varying numbers of nearest neighbors in the SMOTE algorithm (*k* = 1, 5, 15) and compared to models trained without SMOTE. All metrics are averaged across 50 replicate runs for the nine most common species. SMOTE increased test-set recall and F1 score, indicating improved detection of mortality events, but reduced precision. ROC-AUC remained stable across settings, indicating similar rank-based discrimination.

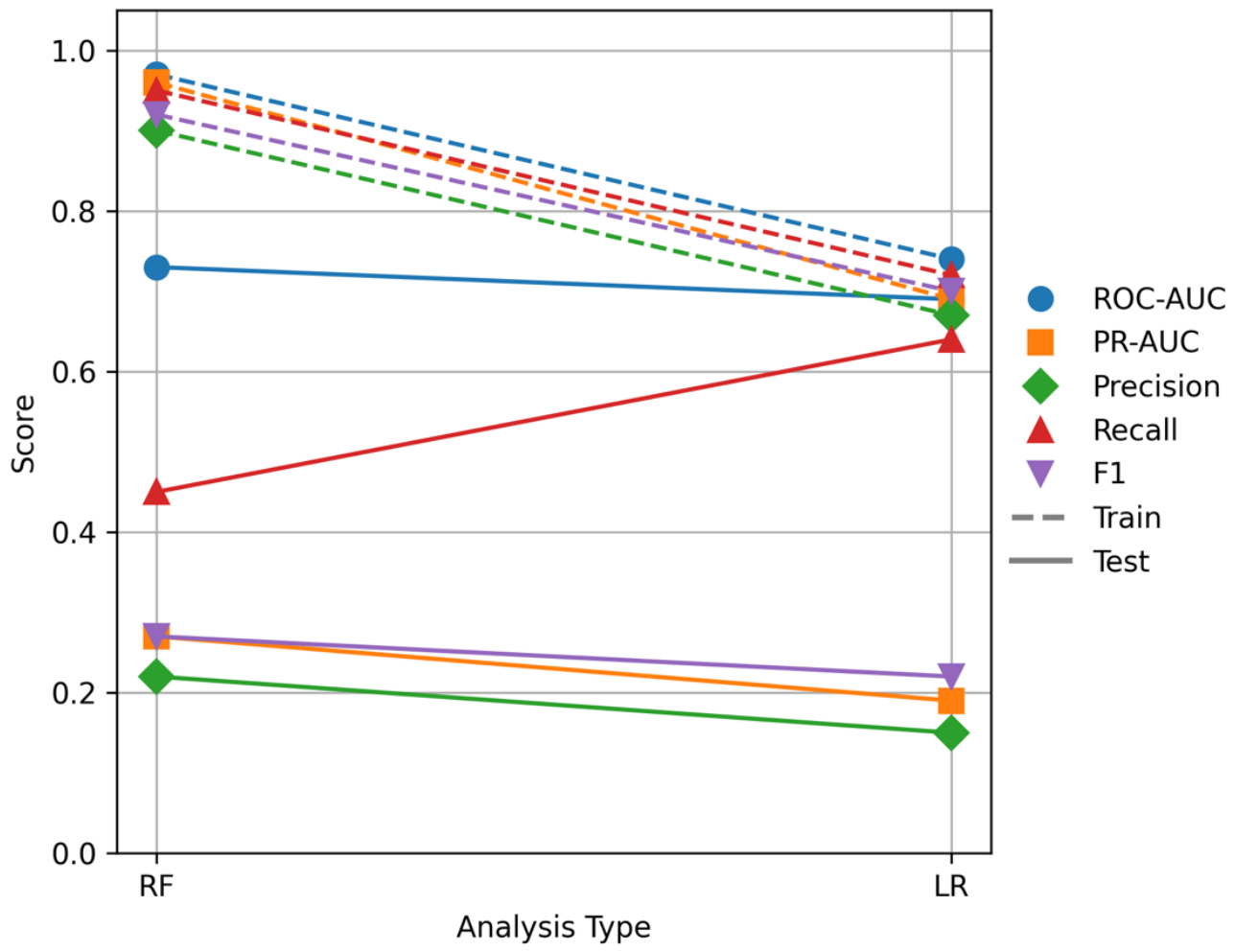

Figure S30. Random forests and logistic regression show similar risk ranking but different precision-recall trade-offs. Comparison of train and test performance between random forest (RF) and logistic regression (LR) classifiers for tree mortality prediction. Model performance was evaluated using ROC-AUC, PR-AUC, precision, recall, and F1 score across 50 replicate runs for all 52 species. Marker color and shape indicate the metric, while line style distinguishes training (dashed) and test (solid) performance. Logistic regression achieved higher test-set recall than RF, indicating that it detected more observed mortality events, but this came at the cost of lower precision, PR-AUC, and F1 score. This pattern is consistent with a less selective classifier that predicts more positive cases. Similar test-set ROC-AUC indicates that both approaches ranked mortality risk comparably.

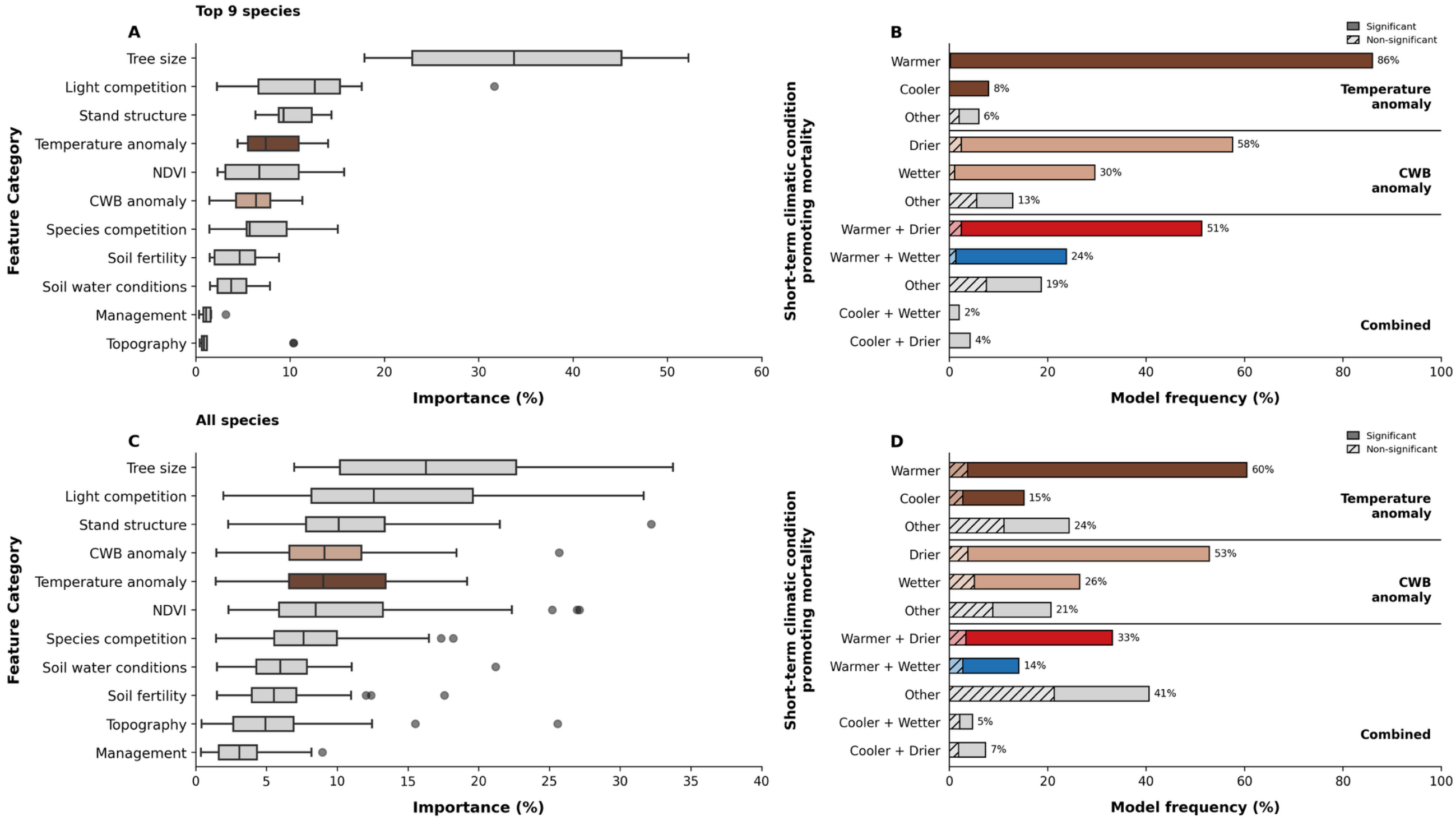

Figure S31. Panels A and B show results for the nine most common species, corresponding to the random forest (RF) analysis shown in Figure 3; panels C and D show results for all 52 species, corresponding to the RF analysis shown in Figure S7. Boxplots in A and C show the relative importance of predictor categories, with each point representing the mean importance across replicate LR runs for one species. Bar charts in B and D show the percentage of LR models associating mortality with warmer, cooler, drier, wetter, or combined short-term climatic anomalies. Darker bar segments indicate statistically significant coefficients, while lighter hatched segments indicate non-significant coefficients. “Other” denotes cases where no consistent response direction was found across models using the same climate feature for a given species, including cases where effects were significant but directionally inconsistent. Overall, LR results broadly agree with the RF patterns: tree size and competition-related variables receive slightly greater relative weight in LR models, but temperature and climatic water balance anomalies remain among the most important stressor-related predictor categories. LR also confirms significant effects for the majority of RF-identified climate features. The split between warmer versus cooler and drier versus wetter responses is broadly comparable to the RF results, although LR shows a higher fraction of models without agreement in response direction.
