# Supplementary material for: Rising tree mortality in France is associated with distinct seasonal climate anomalies
Source: Nat Commun. 2026 Jun 26;17:8016. doi: 10.1038/s41467-026-74613-9 (PMC13454586; doi:10.1038/s41467-026-74613-9)
Supplement: Supplementary file 2 [file 41467_2026_74613_MOESM2_ESM.pdf]

Description of Additional Supplementary Files

Supplementary Data 1: Information on tree species analyzed in this study. Brackets in “Species” denote the abbreviations. “Family” and “Genus” describe the species' scientific classification. “N Trees” denotes the number of trees used in this study. “Absolute increase” refers to the slope from a linear regression of absolute annual mortality rates against year. “Relative increase” refers to the slope from a linear regression of annual mortality rates normalized to 2010 rates against year. “x-fold increase” denotes the ratio by which the mortality rate increased from the first to the last year of data. “pval” refers to the two-sided p-value from a t-test of whether the slope of the linear regression differs from zero (rounded to 0 if below 0.01). Models were fitted for each species separately.

Supplementary Data 2: Candidate feature categories used to model tree mortality. The 183 candidate features were grouped into eleven ecological categories representing complementary mortality pathways, from individual tree vigor and local competition to stand structure, management, site conditions, vegetation dynamics, and short-term climate anomalies. Shown are summaries of the ecological processes represented by each category, the number of features, example features, temporal and spatial resolution, data sources, and the main steps used to derive each feature set. Features derived from the French NFI describe tree- and plot-level conditions at the first visit or changes between visits, whereas external topographic, soil, climate, and NDVI products were spatially matched to NFI plots. Temperature, SPEI, and NDVI features were summarized over the seven-year window preceding the second NFI visit, except where shorter windows were required by data availability.

Supplementary Data 3: Mean importance and standard deviation of each feature category across all models and per species. The feature in brackets denotes the most frequently picked feature across all models. See Supplementary Data 2 for a definition of categories and their features. “ROC-AUC” refers to the mean and standard deviation of the area under the curve of the receiver operator characteristic across all models, after removing models with a ROC-AUC < 0.6.

Supplementary Data 4: Mean model performance across species. Summary of classification performance metrics for each species across all replicate runs. Shown are the number of models (N Models) that met the inclusion criterion (ROC-AUC > 0.6 on the test set) and the mean values of ROC-AUC, PR-AUC, Precision, Recall, and F1 Score for both the training and test datasets. Performance metrics on the training set are consistently high, while test set metrics, particularly PR-AUC and Precision, show substantial variability across species, reflecting differences in model generalization and class imbalance.

Supplementary Data 5: Summary of species-specific responses to temperature features derived from short-term climatic anomalies. The column "change_perc" indicates the percentage of models associating mortality with warmer, cooler, or other short-term conditions for each species. Each temperature feature consists of three attributes (see Methods): the data used to calculate anomalies ("temp_metric", monthly min, max, or mean temperatures), the season for which anomalies were calculated for ("temp_season", win = winter, spr = spring, sum = summer, aut = autumn), and the anomaly type indicating the minimum, mean, or maximum anomaly during the seven years before the second tree visit relative to the 1960-2020 norm ("temp_anom"). For warmer conditions, these anomalies refer to warmer seasonal peak temperatures (max), milder seasonal low temperatures (min), and warmer seasonal average temperatures (mean). For cooler conditions, these anomalies refer to lower seasonal peak temperatures (max), lower seasonal low temperatures (min), and lower seasonal average temperatures (mean). The column "feature_temp" lists the occurrence of all features selected across all models for the given species and short-term climatic conditions. The percentages in brackets indicate the fraction of models associating mortality with the given feature characteristic.

Supplementary Data 6: Summary of species-specific responses to climatic water balance features derived from short-term climatic anomalies. The column "change_perc" indicates the percentage of models associating mortality with drier, wetter, or other short-term conditions for each species. Each SPEI feature consists of three attributes (Supplementary Data 2): integration timescale for calculating SPEI ("spei_duration", short = 1 to 3 months, medium = 6 to 9 months, long = 12 to 24 months), the season from which SPEI is calculated backwards using the last month of the season ("spei_season", win = winter, spr = spring, sum = summer, aut = autumn), and the anomaly type indicating the minimum, mean, or maximum anomaly during the seven years before the second tree visit relative to the 1960-2020 norm ("spei_anom"). For drier conditions, these anomalies refer to drier seasonal lows in water availability (min), drier seasonal peaks (max), and drier seasonal average conditions (mean). For wetter conditions, these anomalies refer to refer to wetter seasonal lows in water availability (min), wetter seasonal peaks (max), and wetter seasonal average conditions (mean). The column "feature_spei" lists the occurrence of all features selected across all models for the given species and short-term climatic conditions. The percentages in brackets indicate the fraction of models associating mortality with the given feature characteristic.

Supplementary Data 7: Summary of species-specific responses to combined temperature and climatic water balance features derived from short-term climatic anomalies. The column "change_perc" indicates the percentage of models associating mortality with warmer-wetter, warmer-drier, cooler-wetter, cooler-drier, or other short-term conditions for each species. Column "feature_temp" lists the frequency of all temperature features that were associated with a given climate condition, encoded as described in Supplementary Data 2. Similarly, "feature_spei" lists the frequency of the climatic water balance features.

Supplementary Data 8: Species-specific mean predicted additional tree mortality risk across dominant seasonal climate anomalies. Values report the mean change in predicted mortality risk (%) for each species and seasonal climate anomaly, quantified by comparing observed seasonal climate conditions to alternatives in which the respective anomaly was shifted by one standard deviation away from the modelled mortality response (see Methods). Columns correspond to the main temperature and CWB anomaly groups identified in Figures 4-6. Empty entries indicate that no models for the respective species identified the respective anomaly type. Summary rows at the bottom report averages across the nine most common species ("Top 9"), all remaining species ("Other species"), and all species combined ("All species"). If there was no model available for a given combination of species and seasonal climate anomalie, that table cell has been left empty.

Supplementary Data 9: Linear regression results linking tree height to mortality sensitivity under wetter conditions. Results show the slope, intercept, coefficient of determination (R^2), and p-value for each species, based on linear models relating individual tree height to the mean normalized SHAP value of the climatic water balance feature in wetter-associated models. The p-value refers to the two-sided p-value from a t-test of whether the slope of the linear regression differs from zero. Significant positive relationships ($p < 0.05$) indicate that taller trees were more sensitive to wetter conditions in model predictions.
