# Supplementary material for: Rising tree mortality in France is associated with distinct seasonal climate anomalies
Source: Nat Commun. 2026 Jun 26;17:8016. doi: 10.1038/s41467-026-74613-9 (PMC13454586; doi:10.1038/s41467-026-74613-9)
Supplement: Supplementary file 3 [file 41467_2026_74613_MOESM4_ESM.pdf]

nature portfolio

Corresponding author(s): Pascal Schneider

Last updated by author(s): May 15, 2026

Reporting Summary

Nature Portfolio wishes to improve the reproducibility of the work that we publish. This form provides structure for consistency and transparency in reporting. For further information on Nature Portfolio policies, see our Editorial Policies and the Editorial Policy Checklist.

Statistics

For all statistical analyses, confirm that the following items are present in the figure legend, table legend, main text, or Methods section.

n/a	Confirmed	
☐	☒	The exact sample size (*n*) for each experimental group/condition, given as a discrete number and unit of measurement
☒	☐	A statement on whether measurements were taken from distinct samples or whether the same sample was measured repeatedly
☐	☒	The statistical test(s) used AND whether they are one- or two-sided<br>*Only common tests should be described solely by name; describe more complex techniques in the Methods section.*
☐	☒	A description of all covariates tested
☐	☒	A description of any assumptions or corrections, such as tests of normality and adjustment for multiple comparisons
☐	☒	A full description of the statistical parameters including central tendency (e.g. means) or other basic estimates (e.g. regression coefficient) AND variation (e.g. standard deviation) or associated estimates of uncertainty (e.g. confidence intervals)
☐	☒	For null hypothesis testing, the test statistic (e.g. *F*, *t*, *r*) with confidence intervals, effect sizes, degrees of freedom and *P* value noted<br>*Give P values as exact values whenever suitable.*
☒	☐	For Bayesian analysis, information on the choice of priors and Markov chain Monte Carlo settings
☒	☐	For hierarchical and complex designs, identification of the appropriate level for tests and full reporting of outcomes
☐	☒	Estimates of effect sizes (e.g. Cohen's *d*, Pearson's *r*), indicating how they were calculated

Our web collection on statistics for biologists contains articles on many of the points above.

Software and code

Policy information about availability of computer code

Data collection	All data was obtained from the sources specified in the data availability statement below. We used only open-source software (Python, R, QGIS) and custom code for data collection (provided on the public Github-linked Zenodo repository https://doi.org/10.5281/zenodo.14941688
Data analysis	We used only open-source software (Python) and custom code for data analysis (provided on the public GitHub repository https://github.com/padasch/tree_mortality_french_nfi and Zenodo https://doi.org/10.5281/zenodo.14941688). The software version and requirements for reproducibility are in the repository's "README.md" file. All code for data analysis is given in the notebooks of folder "03_model_fitting_and_analysis". More information on reproducibility and a data subset is given in the repositories "README.md" file.

For manuscripts utilizing custom algorithms or software that are central to the research but not yet described in published literature, software must be made available to editors and reviewers. We strongly encourage code deposition in a community repository (e.g. GitHub). See the Nature Portfolio guidelines for submitting code & software for further information.

Data

Policy information about availability of data

All manuscripts must include a data availability statement. This statement should provide the following information, where applicable:

- Accession codes, unique identifiers, or web links for publicly available datasets
- A description of any restrictions on data availability
- For clinical datasets or third party data, please ensure that the statement adheres to our policy

All source data used in this study are freely available. Data from the French National Forest Inventory are available at https://inventaire-forestier.ign.fr/dataifn/. The digital elevation model for France is available at https://geoservices.ign.fr/bdalti. Data on temperature, precipitation, evapotranspiration, soil water conditions, and soil pH are available from SILVAE at https://silvae.agroparistech.fr. NDVI data are available from the European Forest Disturbance Atlas via https://doi.org/10.5194/essd-17-2373-2025. The ESA WorldCover 10 m v200 map and the Global Forest Change v1.10 forest cover map are available through Google Earth Engine. The processed data and code used to produce the results presented in this study are available on GitHub at https://github.com/padasch/tree_mortality_french_nfi and archived on Zenodo at https://doi.org/10.5281/zenodo.14941688. Supplementary Data are provided with this paper.

Research involving human participants, their data, or biological material

Policy information about studies with human participants or human data. See also policy information about sex, gender (identity/presentation), and sexual orientation and race, ethnicity and racism.

Reporting on sex and gender	N/A
Reporting on race, ethnicity, or other socially relevant groupings	N/A
Population characteristics	N/A
Recruitment	N/A
Ethics oversight	N/A

Note that full information on the approval of the study protocol must also be provided in the manuscript.

Field-specific reporting

Please select the one below that is the best fit for your research. If you are not sure, read the appropriate sections before making your selection.

☐ Life sciences ☐ Behavioural & social sciences ☒ Ecological, evolutionary & environmental sciences

For a reference copy of the document with all sections, see nature.com/documents/nr-reporting-summary-flat.pdf

Ecological, evolutionary & environmental sciences study design

All studies must disclose on these points even when the disclosure is negative.

Study description	This study applies random forest models to the French forest inventory data to identify the main drivers of tree mortality.
Research sample	The research sample consists of over 500,000 trees across 52 species from the French National Forest Inventory (2015–2023). The dataset includes a wide range of tree species, ages, and environmental conditions, representing forests across France.
Sampling strategy	The French National Forest Inventory (NFI) data, collected by the National Geographic Institute, encompasses a quasi-systematic sampling approach across France's climatically diverse forested regions. The dendrometric inventory spans a grid-based sampling system on a 10 km x 10 km grid, which shifts by 2 km each year, ensuring a 1 km coverage every 10 years. During annual sampling campaigns, about 7,000 new plots are visited, and another 7,000 plots first visited five years ago are re-visited once. Each visit assesses trees in three concentric subplots based on diameter classes (6, 9, and 15 m radii; minimum diameter at breast height of 7.5cm) alongside measurements on stand structure, ecology, and management.
Data collection	All data was collected by the "Institut national de l'information géographique et forestière" and is freely available at https://inventaire-forestier.ign.fr/dataIFN/
Timing and spatial scale	The dendrometric inventory spans a grid-based sampling system on a 10 km x 10 km grid, which shifts by 2 km each year, ensuring a 1 km coverage every 10 years. During annual sampling campaigns, about 7,000 new plots are visited, and another 7,000 plots first visited five years ago are re-visited once.
Data exclusions	We applied the following filters to detect multiple mortality pathways while reducing noise from errors in the dataset. We removed sites and trees that were not part of the sampling protocol (original variables in the IFN data PEUPNR and CIBLE), sites without coordinates information, and trees without live state assessment (original variables VEGET, VEGET5). Because of significant changes in the tree sampling protocol, we kept only data obtained in 2010 or later (i.e., first visits from 2010 to 2017, respectively, second visits from 2015 to 2023). To avoid introducing any bias to our analysis, we kept any sites with irregular mortality events (e.g., we

kept storm-affected sites because climate anomalies can increase vulnerability towards them) and because random forest models are generally robust against such outliers (e.g., we kept scarcely populated sites with less than four trees of a diameter at breast height above 7.5cm, making up 6% of sites). The final dataset included 566,840 trees (28,975 died between visits) from 49,544 sites.

Reproducibility	No experiments were conducted. We share code and data through GitHub, ensuring reproducibility of our code.
Randomization	Not relevant to our study because we did not conduct any experiments.
Blinding	Not relevant to our study because we did not conduct any experiments.

Did the study involve field work? ☐ Yes ☒ No

Reporting for specific materials, systems and methods

We require information from authors about some types of materials, experimental systems and methods used in many studies. Here, indicate whether each material, system or method listed is relevant to your study. If you are not sure if a list item applies to your research, read the appropriate section before selecting a response.

Materials & experimental systems

n/a	Involved in the study
☒	☐ Antibodies
☒	☐ Eukaryotic cell lines
☒	☐ Palaeontology and archaeology
☒	☐ Animals and other organisms
☒	☐ Clinical data
☒	☐ Dual use research of concern
☒	☐ Plants

Methods

n/a	Involved in the study
☒	☐ ChIP-seq
☒	☐ Flow cytometry
☒	☐ MRI-based neuroimaging

Plants

Seed stocks	N/A
Novel plant genotypes	N/A
Authentication	N/A
