# Supplementary material for: Rising tree mortality in France is associated with distinct seasonal climate anomalies
Source: Nat Commun. 2026 Jun 26;17:8016. doi: 10.1038/s41467-026-74613-9 (PMC13454586; doi:10.1038/s41467-026-74613-9)
Supplement: Supplementary file 4 [file 41467_2026_74613_MOESM5_ESM.pdf]

nature portfolio

Peer Review File

Rising tree mortality in France is associated with distinct seasonal climate anomalies

Corresponding Author: Mr Pascal Schneider

This file contains all reviewer reports in order by version, followed by all author rebuttals in order by version.

Attachments originally included by the reviewers as part of their assessment can be found at the end of this file.

Version 0:

Reviewer comments:

Reviewer #1

(Remarks to the Author)
Flush to Crush: The Paradox of Favourable Springs Leading to Tree Mortality

In this study, the authors analyse data from the French National Forest Inventory (2015–2023) to attribute tree mortality drivers. They found tree mortality primarily driven by forest dynamics and climate anomalies. While it was expected that warmer and drier summer will increase tree mortality, the impacts of wetter warmer springs associated with mortality was surprising.

Studying the associated drivers of tree mortality is an important and timely topic. The dataset from the French NFI seems also to be particularly well-suited to do so. While the authors show some really nice and intriguing results that are definitely worth publishing, the manuscript could gain much more impact if it would be written in more simple terms with a clearer structure and generally a lesser focus on the technical aspects of the analysis but on the actual findings of the study. My major concerns with the current manuscript are:

1) I think the title is somewhat misleading as it suggest a causality, which cannot be drawn from attributing tree mortality to environmental drivers. I would suggest to down-tune the title so that it actually better fits the actual results.
2) Methods:
i) I had difficulties to follow the applied methods and models used, which then also hindered to understand the derived results. For instance, it was difficult to understand the French NFI system from the three sentences provided (L384 ff), this needs to be explained better. Also it needs to mentioned that trees are tagged and re-measured. Further, I suggest to include a Methods flow diagram (similar to Fig S11, less detailed but including more info on the input). It would also be important to understand how annual tree mortality was derived (please explain explicitly why bootstrapping was done) if not all trees were measured but just a subsample of about 7000 plots / year and how the variation in space and time was addressed and has influenced the derived results.
ii) Consider also to move important information from the SI to the main ms, for instance the resolution of the feature data and why 138! features were tested and how this selection of features might have affected model results. I am not an expert on these RF analyses, but is it not comparable to other statistics the more you test the more the likelihood increases that you find positive results. E.g. most features were related to SPEI, if there is cross-correlation among different SPEI features then you will also increase the % importance of feature categories. Again, I do not fully understand how you addressed the spatial and temporal patterns of tree mortality, where they all lumped together in your RF analysis?
3) Results: Throughout the results it was not clear to me when warmer+wetter warmer+drier referred to the entire year or just the spring season? I think this needs to be clarified. Also try to be more consistent when addressing variables/models.
i) Figure 1 is really nice and could even benefit from splitting it in two Figures so that a,b and c can be displayed larger. You could also think about showing the SPEI per season, which should then also highlight the wetter-warmer springs - as this is a major part of your story? I found it difficult to grasp when/where springs were warmer and wetter - as annual precip apparently decreased. Also the range of the Y-axis of E should be similar for all species for comparison (eventually also add in one sentence how the annual tree mortality was derived if trees were only re-measured every 5th-year?).
ii) Fig 3 and 4 are really difficult to understand (at least for me...). Could you also think about other figure types that could present your findings in a more simple way? At least it needs to be clear when you are referring to absolute values/SPEI or

anomalies.
4) Structure and flow of discussion needs to be improved. Consider structuring alongside your research questions. Also I think you need a section on confounding factors and limitations of your analyses, particularly cross-correlation and integration of spatial and temporal patterns could provide bias.
5) I was surprised by this very short Introduction. I would like to see how the research questions were developed based on current literature. Also highlight novelty of your study compared to others.

(Remarks on code availability)

Reviewer #2

(Remarks to the Author)

(Remarks on code availability)
Overall, it looks OK, but running the code is time-consuming, so no running check.

Version 1:

Reviewer comments:

Reviewer #1

(Remarks to the Author)
The revised manuscript has improved substantially compared to the previous version, and I commend the authors for the depth and rigor of their analyses. Overall, the study demonstrates impressive statistical expertise. However, I still have several important concerns related to clarity, conciseness, and manuscript structure that should be addressed. In addition, some of my comments on the previous version appear to have been only partially addressed.

1) Given the complexity of the methods and data analyses, I strongly recommend including a graphical summary or schematic overview of the analytical workflow. This would greatly help readers to grasp the overall approach before engaging with the lengthy Methods section.

2) The Results section is scientifically very strong, and I congratulate the authors on the thoroughness of their work. That said, it would benefit from improved clarity and conciseness. Please ensure that wetter and drier periods are consistently referenced with the relevant season throughout the text. The figures are currently underutilized: references to them are often brief, and the connection between text and figures is not always clear. Please make more effective use of figure subpanels and better integrate the figures into the Results narrative. As a minor suggestion, Figures 3 and 4 could likely be merged. I also encourage a careful revision of all figure captions to improve accessibility for readers with less specialized statistical expertise.

3) I reiterate my previous suggestion that the Discussion would benefit from clearer structure, ideally using subheadings aligned with the main research questions. The Discussion is also relatively short compared to the Results section (approximately 2 versus 6 pages), and additional synthesis may be warranted.

4) The paragraph structure should be reconsidered throughout the manuscript. Paragraphs should generally develop a single coherent line of thought and be more substantial in length. This issue affects both the Methods and Discussion sections and would significantly improve readability and logical flow.

(Remarks on code availability)

Reviewer #3

(Remarks to the Author)
The authors tried to use inventory data from French National Forest and machine learning models to investigate reasons for tree mortality in Europe from 2015 to 2023. The research topic is timely. However, the author did not quantitatively justify different conditions, e.g., warm-dry, warm-wet, that are related to the mortality of different tree species. In other words, I don't think the readers could learn much from descriptions, such as “wetter conditions”, “wetter spring lows”. This hinders the impacts of this study in terms of early prevention and strategic mitigation of tree mortality. Additionally, I am not convinced that warm-dry conditions are the primary reasons for tree mortality, which is similar to the concern of Reviewer #1. I agree that warm-wet conditions are the reasons for overshoot. However, during the dry season or under drought conditions, trees could adjust and/or prevent their canopy water loss through stomata closure before reaching to the points of hydraulic failure. These processes could be justified through sapflow measurements. I don't think the authors reasoning: spring overshoot – summer hydraulic failure has a strong justification. Additionally, under a warm and wet conditions, trees could develop a stronger rooting system to support hydraulic uplift, facilitating water movements to a taller canopy. Thus, It could be that temperature is the key factor causing tree mortality. Finally, the definition of “drought” is unclear to me. Does it refer to

meteorological drought or hydrological drought? So, I don’t think is manuscript is ready to be published from Nature Communications. The detailed comments are as:
L135: “warmer-wetter”, to what extend? What are the temperature and precipitation anomaly values that could define “warmer-wetter” conditions for different species? A same comment for “warmer-drier” conditions.
L137: “topography” could be a factor determining temperature the precipitation conditions. “NDVI” is also highly related to environmental factors including topography, temperature the precipitation. Is it possible that the autocorrelations between these factors have accounted for the relative minor impacts of these factors?
L156: What are the quantitative definitions of these terms for different species, “warmer peaks”, “milder lows”, or “warmer averages”?
L171–178: This paragraph indicates that warm temperatures are the primary cause of tree mortality, regardless of whether conditions are dry or wet, isn’t it?
L190: What does “milder lows” mean? What are the temperature ranges for this term? A similar comment for the caption of Figure 3.
L233–235: The authors have addressed number of models in explaining the mortality under different conditions. I wonder whether the calculated mortality rates across the 500,000 trees (L442–443) differ significantly between warm-dry and warm-wet conditions, based on statistical significance tests. If so, I am not sure if the warm-dry and warm-wet based estimates are necessary.
L235–236: The physical implication of this sentence is confusing. If species are “longer drought tolerance”, how could wetter (a similar comment: how wet? | more quantitative definitions are needed) conditions are associated with the mortality of the species?
L240: What is the average height of “taller species”. With increased water availability, canopy diameters could expand, not just their heights. What’s the justification for the allometry used here?
L249–250: A conclusive or and transition sentence is needed. By reading this sentence, I am confused and wonder if this manuscript is trying to solve this issue or this is a suggestion for future research.
L281–283: Again, it is possible that either dry or wet is not important, and temperature is the dominant factor for tree mortality.
378–379: Sapflow data are needed to justify this statement.
L392: This sentence is hard to understand.

(Remarks on code availability)
I've checked the links in Data Availability. All the links are pointed me sources of data except for:
"Data on soil water conditions and soil pH is freely available at (https://silvae.agroparistech.fr). C.P. provided the temperature, precipitation, and evapotranspiration data (partially available at https://silvae.agroparistech.fr)." I am not sure if this is because of my VPN and/or IP address. I am leaving this to the editorial office to further explore.

Version 2:

Reviewer comments:

Reviewer #1

(Remarks to the Author)
This is the third time I have reviewed this manuscript, and I am very pleased to see that it has improved substantially compared to the earlier versions. All of my previous comments have now been fully addressed. I would like to sincerely thank the authors for their careful, thorough, and thoughtful revisions.

I would also like to congratulate the authors on a comprehensive, well-executed and impactful study, which makes a valuable contribution to the field.

(Remarks on code availability)

Reviewer #3

(Remarks to the Author)
The authors had thoroughly addressed all my comments. The manuscript is clear and well written, and I don't have additional comments.

(Remarks on code availability)

Open Access This Peer Review File is licensed under a Creative Commons Attribution 4.0 International License, which permits use, sharing, adaptation, distribution and reproduction in any medium or format, as long as you give appropriate credit to the original author(s) and the source, provide a link to the Creative Commons license, and indicate if changes were made.
In cases where reviewers are anonymous, credit should be given to 'Anonymous Referee' and the source.
The images or other third party material in this Peer Review File are included in the article's Creative Commons license, unless indicated otherwise in a credit line to the material. If material is not included in the article's Creative Commons license and your intended use is not permitted by statutory regulation or exceeds the permitted use, you will need to obtain permission directly from the copyright holder.
To view a copy of this license, visit https://creativecommons.org/licenses/by/4.0/

Response to Review of NCOMMS-25-10269A

Summary of Changes

**Title**	Changed to “Hidden Climate Stressors: A Complex Interplay of Climate Anomalies Shapes European Tree Mortality” to avoid implying causality of structural overshoot and to highlight the role of diverse seasonal climate anomalies. (The original title of the first submission was: “Flush to Crush: The Paradox of Favourable Springs Leading to Tree Mortality”)
**Abstract**	Adjusted wording to shift focus from structural overshoot to variability in climate anomalies.
**Introduction**	Partially rewritten to provide a clearer link to previous research, highlight the novelty of applying interpretable machine learning to forest inventory data, and state three guiding research questions.
**Results**	Largely rewritten to align with the three research questions and to contextualize tree mortality trends. Added sensitivity analyses demonstrating robustness, removed technical details, unified terminology, and restructured sections to present species-level results before tree-level results (swapping the positions of original Figures 5 and 6). Revised figure captions for clarity and changed section titles into concise summaries.
**Discussion**	Largely rewritten to focus on interpretation rather than repetition of results. Balanced discussion of seasonal climate anomalies, treated structural overshoot more cautiously, improved readability, and added a new paragraph on limitations and research opportunities.
**Methods**	Completely rewritten to improve clarity and precision. Added context on national forest inventory methodology, mortality rate estimation, and treatment of spatio-temporal correlation. Included additional requested details and sensitivity analyses of the modelling framework. Updated code and data repositories accordingly.
**Supplementary Information**	Expanded description of feature calculation and logistic regression analyses. Added multiple figures and tables for context and ensured cross-referencing in the main text.

Specific replies to reviewer comments below are written in blue color.

Reviewer #1

In this study, the authors analyse data from the French National Forest Inventory (2015-2023) to attribute tree mortality drivers. They found tree mortality primarily driven by forest dynamics and climate anomalies. While it was expected that warmer and drier summer will increase tree mortality, the impacts of wetter warmer springs associated with mortality was surprising.

Studying the associated drivers of tree mortality is an important and timely topic. The dataset from the French NFI seems also to be particularly well-suited to do so. While the authors show some really nice and intriguing results that are definitely worth publishing, the manuscript could gain much more impact if it would be written in more simple terms with a clearer structure and generally a lesser focus on the technical aspects of the analysis but on the actual findings of the study.

We thank the reviewer for their thoughtful and constructive feedback, which greatly improved our manuscript. In response, we (i) revised the title to avoid implying causality and better reflect the broad analysis of seasonal climate anomalies; (ii) expanded the Introduction to clarify research questions, novelty, and links to literature; (iii) rewrote the Methods to provide

a detailed explanation of the French NFI sampling scheme, bootstrapped mortality estimates, and how our modeling framework accounts for correlating features; (iv) revised the Discussion to better follow the main research questions and added a dedicated limitations section.

My major concerns with the current manuscript are:

R1.1: I think the title is somewhat misleading as it suggest a causality, which cannot be drawn from attributing tree mortality to environmental drivers. I would suggest to down-tune the title so that it actually better fits the actual results.

We agree that the original title implied a causal mechanism that our data do not allow us to establish. We therefore changed the title to "Hidden Climate Stressors: A Complex Interplay of Climate Anomalies Shapes European Tree Mortality." The revised title emphasizes associations and complexity rather than causality, which we believe better reflects the nature of our results.

R1.2: I had difficulties to follow the applied methods and models used, which then also hindered to understand the derived results. For instance, it was difficult to understand the French NFI system from the three sentences provided (L384 ff), this needs to be explained better. Also it needs to mentioned that trees are tagged and re-measured.

We revised the description of the French NFI extensively in the Methods (L424). The new version explains the sampling system in more detail (i.e., the two 5-year cycles of first and second visits, and that all trees are tagged and revisited once after five years, L432). We also added a schematic illustration of the NFI sampling procedure in the Supplementary Information (Fig. S17), but kept it in the Supplementary Information to maintain the manuscript's focus on the main results.

R1.3: Further, I suggest to include a Methods flow diagram (similar to Fig S11, less detailed but including more info on the input).

For more information on the input data, we directly improved the methodology description (L487ff), revised the feature description in the Supplementary Information, and added a new summary table of all features and their data sources (Table S2), and an explicit table for the meaning of the different climate anomaly types (Table S5). In place of an additional Figure, these changes should clarify the input data sufficiently.

R1.4: It would also be important to understand how annual tree mortality was derived (please explain explicitly why bootstrapping was done) if not all trees were measured but just a subsample of about 7000 plots / year and how the variation in space and time was addressed and has influenced the derived results.

As mentioned in response to R1.2, we state now explicitly that all trees are revisited after five years so that mortality rates can be estimated over an average five-year time period. Additionally, we clarified we used bootstrapping to quantify the uncertainty of mortality rates across space and time; similar to what was done in previous studies (L453). We also added additional analyses on mortality trends across regions, size classes, and using basal area changes, following the same rationale (see response to R2.2 below).

R1.5: Consider also to move important information from the SI to the main ms, for instance the resolution of the feature data and why 138! features were tested and how this selection of features might have affected model results. I am not an expert on these RF analyses, but is it not comparable to other statistics the more you test the more the likelihood increases that you find positive results. E.g. most features were related to SPEI, if there is cross-correlation among different SPEI features then you will also increase the % importance of feature categories. Again, I do not fully understand how you addressed the spatial and temporal patterns of tree mortality, where they all lumped together in your RF analysis?

We completely rewrote the Methods to address these concerns. We now describe the resolution and source of all feature data in more detail (L487) and provide a summary table in the new Supplementary Table S2. We emphasize two main ways in which our approach avoids over-inflated importance scores and aims at creating the most parsimonious models. First, we emphasize that spatial and temporal correlations in the data, as well as correlations among features, are broken up at multiple stages (random train-test splits, SMOTE oversampling, bootstrapping within the random forest routine, and the fact that only a random subset of features is considered at each split in a decision tree; L119, L543, and L582). Second, we highlight that the recursive feature selection ensures that only one feature per category (e.g., one temperature anomaly feature, or one tree size feature) ends up in the final random forest model, and that there is an additional removal of correlating features (L119, L569). This approach provides parsimonious, equifinal models of eleven non-correlated features (one per feature category) and ensures interpretability while avoiding bias or importance inflation due to the large initial feature set (L527).

R1.6: Throughout the results it was not clear to me when warmer+wetter warmer+drier referred to the entire year or just the spring season? I think this needs to be clarified. Also try to be more consistent when addressing variables/models.

We thank the reviewer for catching this inconsistency. In the original manuscript, the referencing of seasons was sometimes unclear, especially when “warmer–wetter” or “warmer–drier” models were used as shorthand. In the revision, we clarify that our analyses always refer to seasonal anomalies, never to whole-year conditions (L58, L153ff), and we now explicitly state the relevant season wherever appropriate (for example, seasonal timing was not pertinent and thus omitted when testing correlations with species traits in Figure 6). We also specify now when warmer and wetter anomalies co-occurred, noting that seasonality does not always overlap. For example, wetter springs coincided with warmer springs in only about 30% of warmer–wetter models (L176). Finally, we emphasize in the new limitations paragraph (L397) that connecting mortality more directly to specific seasonal anomalies requires higher-resolution data and should be a focus of future research.

R1.7: Figure 1 is really nice and could even benefit from splitting it in two Figures so that a,b and c can be displayed larger. You could also think about showing the SPEI per season, which should then also highlight the wetter-warmer springs - as this is a major part of your story? I found it difficult to grasp when/where springs were warmer and wetter - as annual precip apparently decreased. Also the range of the Y-axis of E should be similar for all species for comparison (eventually also add in one sentence how the annual tree mortality was derived if trees were only re-measured every 5th-year?).

We thank the reviewer for the constructive suggestions regarding Figure 1. We enlarged panels A-C as recommended, but decided against splitting the figure to maintain coherence between the mortality results and their spatial comparison with climate trends. For panel E, we retained different y-axis ranges to reflect variation in mortality magnitude across species, but now explicitly note this in the caption. We also clarified in the Methods that, because trees are revisited only once, mortality rates are estimated by grouping trees by revisit year and calculating a rolling five-year average. The figure caption now specifies that the regional mortality map is weighted by species occurrence due to the bootstrapping procedure.

To provide additional seasonal climate context, we added Supplementary Figure S16 showing SPEI variability over the past two decades, which shows differences in recent drought events and indicates that the 2018 drought was preceded by an unusually wet spring. In line with our revised interpretation of the structural overshoot hypothesis (see replies to Reviewer #2 below), we chose not to add maps of where wetter springs increased predicted mortality risk; instead, we emphasize in the new paragraph on limitations (L397) that further research is needed to attribute elevated mortality specifically to the 2018 spring and its geographic distribution.

R1.8: Fig 3 and 4 are really difficult to understand (at least for me...). Could you also think about other figure types that could present your findings in a more simple way? At least it needs to be clear when you are referring to absolute values/SPEI or anomalies.

We recognize the complexity of these figures, which reflect the multidimensional nature of the climate variables, i.e., season × anomaly type × temperature metric or event duration of SPEI (Standardized Precipitation-Evapotranspiration Index). However, we believe the pie chart approach is the most effective way to visualize model frequencies across these three dimensions. To aid comprehension, we simplified the language in the figure, caption, and text to be more intuitive (e.g., warmer temperature peak, drier low in water availability). We also revised the reading examples in the captions. In the Methods, we explicitly describe how climate variables were split and displayed (L663), along with a lookup table (Table S5) to clarify the meaning of the different combinations of anomaly type × temperature metric or SPEI event duration. We hope these revisions make the results easier to comprehend without changing the fundamental visualization. Climate anomalies always refer to site-specific deviations from the 1960-2020 long-term trend, as mentioned in the results (L129) and the methods (L509).

R1.9: Structure and flow of discussion needs to be improved. Consider structuring alongside your research questions.

We now clearly introduce three research questions (L67) and use them to guide the discussion (L307ff). In line with the revised title, the discussion has been restructured to balance attention across different climate anomalies, while still giving more detailed consideration to wetter springs as this represents the most novel and unexpected finding. The results were also reorganized to move from broad to narrow, with sections progressing from overall mortality trends to key drivers, then to the characteristics of climate anomalies, species-level differences, and finally tree-level responses. To support this logic, we switched the order of Figures 5 and 6. In addition, subsection titles were rewritten to be more descriptive, and technical details were transferred to the Methods to improve readability.

R1.10: Also I think you need a section on confounding factors and limitations of your analyses, particularly cross-correlation and integration of spatial and temporal patterns could provide bias.

As noted in our reply to R1.5, we consider spatio-temporal correlation and correlations among features to be of limited concern, because they are broken up at multiple steps in our modelling framework. We now also emphasize that our use of 50 model replicates per species directly addresses the challenge of potential biases from using different data subsets, thus accounting for spatio-temporal correlations (L76, L117). Furthermore, we added a dedicated paragraph that discusses constraints of the NFI design, the indirect role of biotic agents, and the need for finer-scale data to test whether the species-specific anomalies identified here reflect underlying mortality processes (L397).

R1.11: I was surprised by this very short Introduction. I would like to see how the research questions were developed based on current literature. Also highlight novelty of your study compared to others.

We revised and extended the Introduction. It now explains how our research questions build on previous studies (L48) and highlights three novel aspects of our approach: explicit consideration of distinct seasonal climate anomalies (L57), the use of random forest models on European NFI data (L60), and repeated analyses across multiple random data subsets to identify consistent mortality patterns (L57, L76). These additions provide a more explicit rationale and underscore the novelty of the study.

Reviewer #2

The authors present interesting findings regarding tree mortality patterns and the potential role of structural overshoot. The analysis is difficult to understand due to methodological ambiguities and explanatory gaps. Some of the data processing and analytical approaches are inadequately described, and lacking connections between different analytical steps, making the study difficult to comprehend and evaluate fully. Overall, I recommend the manuscript for major revision before it can be properly evaluated and make a substantive contribution to the field.

We thank the reviewer for their comprehensive and insightful feedback. In summary, we (i) revised the title and narrative (in line with our response to Reviewer #1) to avoid implying causality and to reflect the study's broader focus on seasonal climate anomalies; (ii) added requested context for mortality trends through new analyses of basal area change, size-class-specific mortality, and the role of harvest; (iii) substantially expanded the Methods, including detailed descriptions of the NFI protocol, the modeling framework, and the calculation of climate features; (iv) incorporated the requested analyses on model evaluation metrics and sensitivity analyses; (v) improved explanations of analyzing and presenting SHAP values at the tree-level; and (vi) added a dedicated limitations section.

Comments and Replies:

R2.1: Lines 25-27: You emphasize "background mortality" in the introduction, but Lines 395-396 reveal an ambiguity by including "sites with irregular mortality events" in your analysis. Without clear differentiation between background and catastrophic mortality, potential drivers could be confounded. Please either clarify how you operationally distinguished between these mortality types or explicitly acknowledge their combined analysis.

We revised the Methods to clarify that we did not distinguish between background and acute mortality events, as our aim was to capture the integrated mortality risk associated with seasonal climate anomalies across all event types (L480). This approach reflects the ecological reality that background stress and acute events often interact and cannot be reliably separated, given the five-year revisit interval of the French NFI. Treating all events together also avoids introducing bias from subsetting the dataset (L57, L76), while the use of random forests reduces sensitivity to outliers, ensuring that high-mortality site outliers do not disproportionately influence results (L550). We now additionally acknowledge in the Discussion that the strong role of tree size and light competition likely reflects background mortality (Figure 2), consistent with previous studies (L314).

R2.2: Lines 73-76: The 1.5-fold to 4.0-fold mortality rate increases are striking but lack ecological context. These shocking numbers need careful explanation. Are these increases balanced by changes in productivity and recruitment? Readers could misunderstand this if these patterns represent forest degradation or simply shifts in forest dynamics.

We contextualized the mortality trends through several additional analyses. We extended regional trend analyses to compare the nine most common species with all species, which showed similar overall patterns but higher uncertainty in less forested regions (L466, Figure S2). We also assessed alternative treatments of harvested trees (as survivors, dead, or excluded entirely; Figure S3), showing that true mortality rates could be even higher under the latter assumptions. To link mortality to broader forest dynamics, we quantified changes in basal area due to mortality, harvest, and growth (Figure S4), finding that mortality-related losses increased while growth gains declined, resulting in net basal area loss for most

species. Finally, size-class-specific analyses (Figure S5) revealed consistently rising mortality across all classes. We discuss these results and suggest that the observed mortality trends reflect a general decline in forest health rather than neutral shifts in forest dynamics (L92).

R2.3: L109: Before presenting the SHAP analysis, please report the overall performance (R^2) of the Random Forest models and quantify what percentage of mortality variation cannot be explained by your selected drivers.

Because our models are binary classifiers, R^2 is not an appropriate performance metric. Instead, in addition to the previously reported ROC-AUC and PR-AUC, we now also provide precision, recall, and F1-scores for all species (Table S4), as also requested in R2.9. These complementary metrics are described in the Methods and in the context of the additional sensitivity analyses (see replies to R2.9 and R2.10), and the main text refers readers to these additional metrics (L122).

R2.4: The method for SHAP analysis is inadequately explained. For example, in Figure 2A, you present a single "temperature anomaly" variable, but your methods indicate you used multiple temperature variables. How did you merge these different variables? Similarly, in Figure 2B, how did you handle correlations between temperature variables?

Thanks for raising this question, as our description of how Figures 2 and 3 were created was not adequately detailed. We revised the results (153ff) and the methodology (L529ff) section accordingly. To clarify, each random forest model contained only a single feature per category (e.g., one temperature feature, one climatic water balance (CWB) feature), selected through recursive feature selection, plus removal of remaining correlations if needed. This step affected mainly competition-related features and led to the removal in about 3% of the 450 models for the nine most common species and about 20% of models across all species (L576). Thus, there were no correlations among features within the final models used to produce our results. While climate features may correlate across models, each model fitting routine independently selected the single best temperature or CWB feature based on the randomness introduced by data heterogeneity and model fitting. This design ensures that the signals we interpret are not driven by within-model correlation structure.

To generate Figures 2 and 3, we aggregated results across the 50 models per species, covering the nine most common species (450 models in total, L529). For each model, the selected temperature feature was decomposed into its three dimensions (L153, L659): season, metric (monthly mean, minimum, maximum temperature), and anomaly type (seven-year mean, minimum, maximum anomalies prior to revisit). And analogously for CWB features: season, SPEI integration timescale (1-24 months), and anomaly type (seven-year mean, minimum, maximum anomalies prior to revisit). Figure 2 displays temperature features with season on the y-axis, metric on the x-axis, anomaly type shown by color shade, and the frequency of each combination represented by the size and slices of the pie charts. CWB features were decomposed and visualized in the same way (Figure 3). These figures therefore summarize consistent patterns that emerge from aggregating across all models rather than from any single temperature or CWB feature.

R2.5: You state, "For Fagus sylvatica, 72% of the models associated mortality with drier conditions and 28% with wetter" which conflicts with "84% of the models associated mortality with drier conditions and 16% with wetter conditions." Please clarify this discrepancy.

Thank you for spotting this inconsistency. This was a typographical error, which we have corrected (L273). These percentages (84% and 16%) refer exclusively to the 50 replicate models for Fagus sylvatica, not to all models across species.

R2.6: Figure 5 is difficult to understand without proper explanation. Define "vulnerability" before using it, and clarify how the scaled SHAP values calculated, and could show figures to explain it should be better, and how they relate to those in Figures S7/S8.

To avoid implying causality, we now refer to the correlation between SHAP values and feature values as “sensitivity” rather than “vulnerability,” as this term better reflects how increases in feature values affect predicted mortality risk. We added a clear definition of how each tree’s sensitivity to climate anomalies was calculated and explained how Figures 6 (previously Figure 5), S12, and S13 were generated (L272, L674ff). In brief, sensitivity is represented by SHAP values that quantify the marginal effect of a given temperature or climatic water balance feature on predicted mortality risk relative to other features in the same model. To calculate it, SHAP values for each temperature and CWB feature were normalized within each model and then averaged across all models showing a consistent mortality response (i.e., drier or wetter for CWB, and warmer, cooler for temperature), yielding a single tree-level sensitivity score. These scores were used in Figure 6 to illustrate cross-species patterns with bivariate density plots and Spearman correlations. Additional figures (S12, S13) show how sensitivity to temperature or CWB anomalies varied depending on the year of the second tree census.

R2.7: Figures S16 and S17 are not mentioned in the main text. Please ensure all supplementary materials are properly referenced and support the main text.

Thank you for catching this. We made sure to have all supplementary materials referenced in the main text.

R2.8: Lines 446-448: For the SMOTE, please specify the k-value used for the nearest neighbor algorithm and address how uncertainty due to choice of k value in synthetic sampling affects model reliability.

We agree that uncertainty in the choice of k for SMOTE could influence results and have addressed this in the revision. We now specify that the nearest-neighbor algorithm was run with the default setting (k = 5, L608ff). To test sensitivity, we re-ran the full analysis for the nine most common species using alternative values (k = 1 and k = 15; Figures S26 and S27). Model performance remained stable (Figure S29). Although higher k slightly increased the frequency of models linking warmer-wetter anomalies to higher mortality risk, the proportion among identified climate anomalies was unchanged. We also tested an approach without oversampling (k = 0, Figure S28), corresponding to simple class balancing by replicating mortality events. This substantially reduced recall on the test set and led to fewer models linking warmer-wetter conditions to increased mortality risk (Figure S27). We now note in the Methods that these analyses demonstrate our conclusions are robust to the choice of k in SMOTE, and that oversampling improved the models' ability to detect less common mortality events, as reflected by higher recall (L616).

R2.9: While you correctly applied SMOTE only to training data to prevent leakage, your validation approach needs strengthening. You must verify synthetic samples represent realistic mortality scenarios, not just statistical constructs. Include clear cross-validation procedures, proper metrics for imbalanced data (precision, recall, F1-score, AUPRC), and analyze how synthetic sampling uncertainty affects ecological interpretation.

We thank the reviewer for raising this important verification of SMOTE samples. In the revised manuscript, we now explicitly describe that we ensured the ecological plausibility of synthetic samples by comparing the data distribution of the original, the balanced training, and the test data using principal component analyses and Mahalanobis distances (Figures S20 and S21; L556).

Model validation during training relied on the out-of-bag error (L562), complemented by comparing predictions to the independent held-out test set. To strengthen evaluation under class imbalance, we complemented ROC-AUC and PR-AUC with additional metrics, including precision, recall, and F1-score (Table S4; L600). Finally, we assessed uncertainty due to synthetic sampling by repeating the analysis with alternative k-values for the SMOTE algorithm and without oversampling, which yielded similar results (see reply R2.8). These analyses confirm that synthetic samples remained ecologically realistic and that our conclusions are robust to the synthetic oversampling procedure.

R2.10: Lines 462-464: The decision to keep exactly two features per category during feature selection seems arbitrary. Was this empirically determined? Did you test whether some categories might deserve more features than others? What are these features, temperature itself has many variables for different seasons. Unclear enough and so hard to understand it well.

Our aim was to identify the single best predictor per category, thereby maintaining parsimony and interpretability while allowing robust signals to emerge across many plausible feature combinations (L71, L119). To achieve this, we retained two features per category prior to final selection as a pragmatic default to ensure that no feature category was excluded too early in the process. To test whether this choice influenced the results, we repeated the full modelling routine while retaining 4 or 6 features per category before reducing to the single best feature. Both the resulting feature sets and model performances were highly consistent across these tests. We now describe this sensitivity analysis in the Methods (L608) and provide the results in Figures S23-S25.

R2.11: Lines 425-429: For SPEI calculation, you mention testing timescales from 1-24 months but never specify which ones were most informative. The choice of evapotranspiration formula can significantly affect drought index values - which one did you use?

As specified in our reply to R1.5, each model contained only a single SPEI feature selected through recursive feature selection. Because different models could select different SPEI features, we compared results across models and decomposed the selected features into their attributes of season, timescale, and anomaly type (see reply to R2.4). The most frequently selected, and thus most informative, SPEI timescales are shown in Figure 4 and discussed in the Results section on drought-type groups (L163). The relevant timescale varies by drought type: on-average drier summers and reduced maximum water availability in spring or summer occur within one to three months, whereas a third group represents more severe multi-month droughts peaking in autumn.

For potential evapotranspiration, we relied on existing climate data for France calculated with Turc's formula. We did not perform new calculations, and all sources are listed in the Supplementary Methods and in the new Table S2. Although different evapotranspiration formulas may yield somewhat different drought indices, a systematic comparison was beyond the scope of this study.

R2.12: Lines 422-429: The climate data description seems lack crucial details about spatial resolution, data sources, and how you matched climate data to individual tree locations. For sites with complex terrain, was any microclimate adjustment performed?

We expanded the Methods (L487) and Supplementary Information (summarized in new Table S2) to provide more details on the sources, spatio-temporal resolution, and calculation of all features. The climate data were available at monthly resolution and 1 km spatial resolution. Microclimate variability is partly captured by the underlying climate data and directly by explicitly including topographic features as predictors. Nonetheless, we agree that microclimate can be important in heterogeneous landscapes, but plot-level adjustments were not feasible because French NFI plot coordinates are only provided within a 700 m uncertainty radius for privacy reasons (see Supplementary Information). Given this uncertainty, the 1 km resolution of the gridded climate data is appropriate and consistent with the spatial accuracy of the plot locations.

R2.13: Lines 487-492: Your SHAP analysis lacks technical specifics. Which SHAP algorithm variant did you use? How did you aggregate SHAP values across the ensemble of trees?

We now provide additional technical details on the SHAP analysis (L631). SHAP values were computed using the interventional TreeExplainer algorithm implemented in the Python "shap" package, which is specifically optimized for tree-based models such as random forests. The algorithm quantifies each feature's marginal contribution to the baseline expected value (the mean prediction across the dataset). Contributions are first calculated within each decision tree and then averaged across the ensemble, yielding a single SHAP value per feature for each observation. All calculations were performed on the held-out test data to ensure generalizable behavior.

R2.14: Lines 506-508: The 60% threshold for consistent responses across models seems arbitrary. A sensitivity analysis would strengthen confidence in your classification approach.

We clarified in the Methods that the 60% threshold was chosen to ensure that the majority of models for a given species agreed on the direction of the climate-mortality response for the same temperature-SPEI feature pair (L654). To test robustness, we repeated the analysis with a more conservative threshold of 90%. This increased the proportion of model pairs classified as inconsistent from 3% to 10% overall (and from 0% to 5% for the nine most common species), but had negligible effects on the main results.

R2.15: The structural overshoot hypothesis, central to your paper, is supported only by indirect evidence. Consider using available methods to directly quantify structural overshoot to strengthen your conclusions. There are literatures and data (in situ data or remote sensing prodcuts) to support your analysis.

In line with our replies to R1.1 and R2.26, we revised the manuscript title to avoid implying direct evidence for the structural overshoot hypothesis. While structural overshoot remains a plausible explanation for the observed link between favorable spring growth conditions and subsequent mortality, direct quantification would require datasets with annual growth and mortality resolution, which are not available at the scale of the French NFI. Explicitly testing this mechanism was therefore beyond the scope of our study. Instead, we strengthened the manuscript by citing global remote-sensing, vegetation modelling, and tree-level studies that provide independent support for the structural overshoot hypothesis (L358ff). We also expanded our analyses at both the species and tree level to place our findings in this broader context (see reply to R2.27).

R2.16: Lines 476-478: You mention comparing random forests with logistic regression models but provide no details or results from this comparison.

Logistic regression models are the standard approach in many tree mortality studies, and we therefore used them as a supplementary benchmark to validate the patterns identified by the random forests. To avoid overloading the Methods, we originally described this analysis only in the Supplementary Information, but we now also summarize the key results in the main text (L622). In brief, we fitted 2,600 logistic mixed-effects models using the same training/test splits and the same eleven features selected through recursive feature elimination for the final random forest models. These logistic models produced similar ROC-AUC values but substantially lower recall (Figure S30). GLMMs also tended to assign greater importance to competition-related variables while down-weighting climate variables (Figure S31), likely because

nonlinearities and interaction effects captured by random forests are not explicitly modelled in GLMMs. Despite these differences, both approaches yielded consistent results (Figure S31).

R2.17: Lines 519-521: For species trait correlations, did you consider phylogenetic non-independence? Closely related species often share trait values due to evolutionary history rather than independent adaptation.

We agree that closely related species may share traits due to evolutionary history rather than purely adaptive processes. Accounting for phylogenetic non-independence, however, would add considerable methodological complexity and was beyond the scope of our study. Instead, we relied on established drought and shade tolerance indices that directly capture functional adaptations relevant to our research questions. Importantly, our results show substantial variability within genera (e.g., Quercus in Figure 5, previously Figure 6), indicating that trait-mortality relationships are not solely explained by phylogenetic relatedness but also reflect ecological differentiation among closely related species.

R2.18: Lines 404-405: Your assumption that harvested trees were "survivors" is significant. How many trees fell into this category? In managed stands, this could seriously skew your mortality estimates, especially if salvage logging practices varied by region or species.

We now explicitly acknowledge that treating harvested trees as survivors provides a conservative estimate of mortality rates (L92, L445), since some may have died before removal through salvage logging. To evaluate potential bias, we conducted a sensitivity analysis in which harvested trees were alternatively (i) treated as dead or (ii) excluded from the mortality rate estimation (Figure S3). Assuming harvested trees had died yielded substantially higher rates, representing an upper-limit estimate, while excluding them produced results similar to treating them as survivors. Notably, Picea abies showed particularly elevated rates under the salvage logging assumption, consistent with intensive post-bark beetle harvesting in northeastern France. At the same time, harvest rates were relatively stable or declining for most species over the study period (Figure S4; see reply to R2.2), indicating that rising mortality cannot be attributed solely to management practices or demographic shifts. Thus, irrespective of how harvests were treated, the overall trend of increasing mortality rates remained consistent.

R2.19: Lines 383-389: The NFI sampling methodology is explained, but how the shifting grid pattern ensures complete coverage remains unclear. A simple diagram in the supplementary materials would help clarify this.

We revised the description of the French NFI sampling protocol to clarify how the rotating five-year grid ensures full spatial coverage in forested areas (L424). To aid comprehension, we also added a schematic adapted from IGN's official documentation (Figure S17) that illustrates the shifting grid and revisit design, showing how plots are revisited once after five years before being retired and replaced by nearby plots. In addition, we extended Figure S2 to show regional trends and acknowledge that less densely sampled regions (due to limited forest cover) exhibit wider uncertainty ranges than densely forested regions (L462).

R2.20: More discussion of potential sampling biases in the NFI dataset would be helpful. Were there changes in protocol during your study period? Were all forest types equally accessible for sampling? These factors could affect your interpretation of spatial and temporal trends.

We restricted our analysis to data collected after 2010 to ensure methodological consistency, as the French NFI mortality protocol changed substantially at that time (from expert judgment of whether a tree might have died within the past five years to direct reassessment of tree status at both visits; L475). To address potential regional sampling biases, we now present temporal mortality trends per region with bootstrapped uncertainty estimates, calculated both across all species and for the nine most common species (Figure S2). This bootstrapping explicitly accounts for variation in sampling intensity, with less densely forested or less accessible regions (e.g., Grand Ouest, Corse) showing wider uncertainty ranges (L464). It also highlights cases where mortality rates may be skewed by less common but highly vulnerable species, such as Fraxinus excelsior in Centre Nord and Grand Est. While absolute rates vary with species composition, the overall temporal increase in mortality is robust, and spatial patterns are consistent, irrespective of whether the most common or all species are considered (compare Figure 1 and Figure S2).

R2.21: Please clarify how you handle potential disturbances like fire that might affect mortality but aren't explicitly addressed in your analysis.

As noted in our reply to R2.1, we did not separate background mortality from disturbance-driven events such as fire. Our objective was to capture the integrated mortality risk associated with seasonal climate anomalies across all event types (L480). This approach reflects the ecological reality that disturbances and background stress often interact and cannot be reliably disentangled, given the five-year revisit interval of the French NFI. Treating all events together also avoids

introducing bias from subsetting the dataset (L483), while the use of random forests reduces sensitivity to high-mortality outliers, ensuring that individual disturbance events do not disproportionately influence results (L543).

R2.22: Line 480: You set ROC-AUC > 0.6 as your threshold for model retention but don't report how many models failed this test. This information helps assess whether certain species or conditions were particularly difficult to model.

We now report the number of models that did not meet the ROC-AUC > 0.6 threshold (L590) and visualize the performance of the retained models as boxplots for all evaluation metrics (Figure S22; see reply to R2.3). For the nine most common species, only about 2% of the 450 models were discarded, meaning the main results presented in the manuscript were unaffected. Across all 2,600 models, approximately 10% fell below the threshold, again without substantially influencing the main findings. Most of the discarded models corresponded to species with limited data availability and greater variability across random seeds (e.g., Ilex aquifolium), whereas abundant species yielded consistently higher and more stable predictive performance (Figure S22).

R2.23: Lines 411-421: Your feature categories are spread across several paragraphs with embedded references. A simple table listing each category with example features would improve clarity.

We restructured the Methods to present all feature categories in three consecutive paragraphs (NFI data, all categories, and climate features in detail, L473ff). We also revised how climate features are introduced in the Results to improve clarity and readability (L129, L155, L163). In addition, we created a table listing all features and their corresponding feature categories, which is provided in the Supplementary Information due to space constraints (Table S2).

R2.24: Do you include all SPEIs with different month scales in the machine learning model? How do you address the high correlations between them? Similar to the different temperature variables or other variables.

As detailed in our replies to R1.5 and R2.4, each model initially included all features (e.g., multiple SPEI timescales). Recursive feature selection then identified the single most important feature per category (L559). If correlations persisted across categories, we removed the less important feature (L569. This procedure ensured that the final feature set for each species contained only non-correlated predictors and at most one feature from each mortality category. Although random forests are generally robust to correlated predictors, we applied this additional filtering to improve interpretability and avoid inflated importance scores. Thus, while dozens of SPEI and temperature anomaly features were considered at the outset, only a single non-correlated feature from each category was retained in the final models. Robust response patterns were then evaluated across the ensemble of these models to test the consistency of outcomes (L645).

R2.25: Lines 430-434: The seven-year window for "short-term" climate analysis seems arbitrary. Is there ecological reasoning behind this choice? How do you account for lag effects in tree responses that vary by species and climate zone?

The choice of a seven-year window reflects both ecological reasoning and methodological constraints, as we now explain in more detail (L503ff). Tree mortality often lags several years behind climatic stress, and the NFI's five-year revisit interval prevents precise determination of the year of death. By extending the window two years prior to the first census, we consistently capture climate conditions two to seven years before tree death. This range is supported by previous mortality modeling studies and by observations of delayed mortality following events such as the 2018 European drought. We acknowledge that lag lengths undoubtedly vary among species and stressors. However, testing species-specific lags would require higher-resolution temporal data and was beyond the scope of this study. We now also emphasize in the new limitations paragraph that this constraint arises from the temporal resolution of the NFI data (L397).

R2.26: Your title emphasizes spring conditions ("Flush to Crush"), but the discussion section (Lines 270-288) focuses extensively on winter conditions affecting mortality with comparatively less attention to spring effects. This creates a disconnect between your title's emphasis and your discussion. Please provide more balanced analysis of spring conditions to better align with your title.

In line with our response to R1.1, we revised the manuscript title to avoid overemphasizing wetter springs and to reflect the broader focus on the interplay of seasonal climate anomalies. While favorable spring conditions remain an important part of our findings (strengthened by the additional analyses added in response to R2.8, R2.10, and R2.15), the revised title now emphasizes that multiple seasonal anomalies shape mortality risk. This brings the framing of the paper into closer alignment with the balance of the discussion.

R2.27: For Figure 6, you analyze vulnerability to wetter conditions and discuss this around L336-337: "Secondly, wetter springs disproportionately affected taller species (Figure 6C), suggesting that structural overshoot poses a greater challenge for canopy-forming trees." How about the analysis for vulnerability to drier conditions. Does the relationship between tree height and vulnerability to drier conditions also supports your structural overshoot hypothesis?

We extended our analysis to test whether the structural overshoot hypothesis also applied to drier conditions by correlating species' mean height with the frequency of models linking mortality to drier or warmer-drier anomalies. In contrast to the relationships observed under wetter or warmer-wetter conditions, no significant associations were found (L239). This likely reflects the broader range of mechanisms encompassed by drier anomalies, which act through diverse pathways not directly tied to tree size. At the tree level, we regressed normalized SHAP values of CWB features against individual tree height (Methods: L690; Results: L281). While some species showed plausible correlations between height and sensitivity to wetter conditions, no consistent response emerged across species (Table S9), and results remained similarly inconclusive when testing subsets of warmer-wetter, drier, and warmer-drier models. We acknowledge this limitation and argue that these findings suggest structural overshoot may be detectable at the species level, but individual-level heterogeneity can obscure these patterns (L406). Note that Figure 6 in the reviewer comments is Figure 5 in the revised manuscript.

R2.28: Remarks on code availability: Overall, it looks OK, but running the code is time-consuming, so no running check.

For a brief, less time-consuming test of the results for the most common nine species, we provided their data on Zenodo (https://doi.org/10.5281/zenodo.14941688; *all_runs.zip*) along with code usage instructions in the linked GitHub repository (see "README.md" file here: https://github.com/padasch/tree_mortality_french_nfi).

Response to Review of NCOMMS-25-10269A

Author replies are written in blue color below the respective reviewer comment.

Reviewer #1

The revised manuscript has improved substantially compared to the previous version, and I commend the authors for the depth and rigor of their analyses. Overall, the study demonstrates impressive statistical expertise. However, I still have several important concerns related to clarity, conciseness, and manuscript structure that should be addressed. In addition, some of my comments on the previous version appear to have been only partially addressed.

We thank the reviewer for this positive overall assessment and for acknowledging the rigor of the analyses. We carefully addressed the remaining concerns regarding clarity and manuscript structure through a comprehensive revision. In particular, we added a graphical overview of the analytical workflow (new Figure 1), substantially revised the Results section to improve figure integration and seasonal clarity, and revised paragraph structure throughout the Discussion to improve readability and logical flow. Below, we respond in detail to each specific comment.

1) Given the complexity of the methods and data analyses, I strongly recommend including a graphical summary or schematic overview of the analytical workflow. This would greatly help readers to grasp the overall approach before engaging with the lengthy Methods section.

We fully agree with this suggestion. We have added a new Figure 1 that provides a graphical overview of the full analytical workflow. The schematic summarizes the data sources, the construction of seasonal climate anomalies, the modeling framework, and the interpretation of model outputs. In addition, the figure explicitly links each analytical step to the corresponding figures in the main text.

2) The Results section is scientifically very strong, and I congratulate the authors on the thoroughness of their work. That said, it would benefit from improved clarity and conciseness. Please ensure that wetter and drier periods are consistently referenced with the relevant season throughout the text. The figures are currently underutilized: references to them are often brief, and the connection between text and figures is not always clear. Please make more effective use of figure subpanels and better integrate the figures into the Results narrative. As a minor suggestion, Figures 3 and 4 could likely be merged. I also encourage a careful revision of all figure captions to improve accessibility for readers with less specialized statistical expertise.

We thank the reviewer for this positive assessment of the Results section. We have carefully revised this section to improve readability and the integration of figures. Result statements are now more explicitly linked to the relevant figures and their new subpanels (see below), and figure references are used more frequently throughout the text. We also ensured that wetter and drier conditions are always explicitly mentioned alongside the relevant season.

In response to the suggestion regarding figure use, we revised Figures 4 and 5 (previously Figures 3 and 4) by simplifying displayed text and adding subpanels that are used in the text for clearer reference. In addition, we removed model-frequency percentages from the figure legends to avoid confusion and instead report them explicitly in the Results text (L182ff). We deliberately did not merge these figures, as they address distinct but complementary aspects of climate-related mortality risk: Figure 4 focuses on temperature-related effects and highlights the limited differences between models where the concurrent CWB anomaly feature linked mortality to wetter or drier anomalies. In contrast, Figure 5 examines the attributes of the climatic water balance anomalies, distinguishing between drought types and wetter spring conditions. Finally, we revised the captions of Figures 4 and 5 to reduce technical terminology and to more clearly explain how each figure should be interpreted, thereby improving accessibility for a broader readership.

3) I reiterate my previous suggestion that the Discussion would benefit from clearer structure, ideally using subheadings aligned with the main research questions. The Discussion is also relatively short compared to the Results section (approximately 2 versus 6 pages), and additional synthesis may be warranted.

We agree that subheadings help for reader guidance and structure but since they are not typically included in the Discussion section of *Nature Communications*, we leave the final decision regarding their inclusion to the Editor. Research Questions 1 and 2 are addressed jointly in the opening section due to their close connection. Therefore, we utilized headings to clearly distinguish the discussion of Research Question 3. Namely, the expected temperature and drought effects, the unexpected wetter spring effect, and finally the discussion of limitations and future outlook.

We note that the Results section remains longer than the Discussion in terms of page count, primarily because it contains all figures, while the word length of the two sections is more comparable (each approx. 2'300 words). The level of detail in the Results is necessary to document species-specific and seasonal patterns; however, we improved readability by better integrating figures into the text, and adding summarizing and transitional sentences that guide readers through the main findings. These changes were made to make the Results more accessible to a broader readership while retaining the necessary level of detail.

4) The paragraph structure should be reconsidered throughout the manuscript. Paragraphs should generally develop a single coherent line of thought and be more substantial in length. This issue affects both the Methods and Discussion sections and would significantly improve readability and logical flow.

We further revised the Discussion structure to enhance readability and thematic clarity; paragraphs were reorganized to ensure they begin with summarizing the key result before providing interpretation and synthesis in the context of existing literature. Additionally, in response to related comments from Reviewer #3, we expanded the Discussion to provide a more comprehensive synthesis of the results, including the integration and analysis of a new Figure 6 that quantifies the impacts of different seasonal climate anomalies. Given the technical density of the Methods section, we retained relatively short paragraphs to improve readability and navigability.

Reviewer #3

The authors tried to use inventory data from French National Forest and machine learning models to investigate reasons for tree mortality in Europe from 2015 to 2023. The research topic is timely. However, the author did not quantitatively justify different conditions, e.g., warm-dry, warm-wet, that are related to the mortality of different tree species. In other words, I don't think the readers could learn much from descriptions, such as “wetter conditions”, “wetter spring lows”. This hinders the impacts of this study in terms of early prevention and strategic mitigation of tree mortality. Additionally, I am not convinced that warm-dry conditions are the primary reasons for tree mortality, which is similar to the concern of Reviewer #1. I agree that warm-wet conditions are the reasons for overshoot. However, during the dry season or under drought conditions, trees could adjust and/or prevent their canopy water loss through stomata closure before reaching to the points of hydraulic failure. These processes could be justified through sapflow measurements. I don't think the authors reasoning: spring overshoot – summer hydraulic failure has a strong justification. Additionally, under a warm and wet conditions, trees could develop a stronger rooting system to support hydraulic uplift, facilitating water movements to a taller canopy. Thus, It could be that temperature is the key factor causing tree mortality. Finally, the definition of “drought” is unclear to me. Does it refer to meteorological drought or hydrological drought? So, I don't think is manuscript is ready to be published from Nature Communications. The detailed comments are as:

We thank the reviewer for this assessment and for their comments that helped to strengthen the manuscript. Below, we address the specific concerns in detail. Here, we first clarify several overarching points.

First, regarding the definition and quantification of climatic anomalies, we now explicitly clarify throughout the manuscript that terms such as “warmer”, “wetter”, or “drier” refer to standardized anomalies relative to site-specific long-term norms (see details below). We also added that the Standardized Evapotranspiration-Precipitation Index (SPEI) quantifies meteorological rather than hydrological conditions (L559). Also, the term “drought” in this study refers more broadly to anomalously dry climatic water balance conditions. Based on the seasonal and temporal structure of SPEI anomalies, we thus identified three drought types (L191ff; Figure 5 [previously Figure 4]), which provides an interpretation consistent with established frameworks of drought-induced tree mortality[1,2].

Second, we provide results that demonstrate that temperature anomalies and climatic water balance anomalies exert effects of comparable importance, with their influence differing seasonally and across species (see details below). This point is now made more explicit through revised wording and the addition of a new quantitative sensitivity analysis (new Figure 6), which places the relative contributions of warmer, drier, and wetter anomalies on a comparable scale.

Third, we agree that physiological measurements such as sapflow would be valuable to directly test mechanisms related to canopy regulation and structural overshoot. However, such data are beyond the scope of a large-scale inventory-based study covering hundreds of thousands of trees. We therefore deliberately use cautious wording when discussing mechanistic interpretations and frame structural overshoot as a working hypothesis that requires further investigation (L454).

Below, we grouped similar reviewer comments together and address them together.

Comments on the definition and quantification of different seasonal climatic anomalies:

L135: “warmer-wetter”, to what extend? What are the temperature and precipitation anomaly values that could define “warmer-wetter” conditions for different species? A same comment for “warmer-drier” conditions.

L156: What are the quantitative definitions of these terms for different species, “warmer peaks”, “milder lows”, or “warmer averages”?

We agree that the definition and quantification of climatic anomalies was not sufficiently clear in the previous version. We now clarify that climatic anomalies are site- and season-specific standardized anomalies relative to local long-term norm conditions (Results: L142; Methods: L551ff; and example in Figure 1). Thus, terms such as “warmer”, “wetter”, or “drier” are always in units of standard deviations. We chose standardized anomalies for two main reasons. First, they account for the strong heterogeneity in absolute climatic conditions across sites and species and reflect that deviations from local expectations may be more relevant for tree mortality than absolute values (e.g. °C or mm) - the same absolute anomaly may have very different ecological implications depending on the site and species[1,3]. Second, the Standardized Evapotranspiration-Precipitation Index (SPEI) that was used to quantify climatic water balance anomalies uses standardized indices by definition[4], and applying the same scaling rationale to temperature anomalies ensures comparability across climate drivers. Thus, we cannot report any absolute and generally applicable thresholds (e.g. a specific °C increase causing a certain change in mortality risk). In addition, random forest models are inherently non-linear, and the same change in climatic anomaly (irrespective if absolute or relative) does not lead to linearly interpretable mortality responses. For these reasons, we initially restrained from giving any potentially misleading quantification and instead focused on the relative importance and seasonal timing of climatic anomalies.

That said, we agree that clearer quantification of the effects of temperature and climatic water balance anomalies improves interpretability. We therefore added a new analysis estimating a "predicted additive mortality risk" by comparing observed climatic conditions to conditions that are one standard deviation less extreme (new Figure 6, L198 in the Results, 711 in the Methods). Consistent with the feature importance ranking in Figure 3 (previously Figure 2), this analysis shows that the effects of winter, spring, and summer warming are overall comparable, albeit slightly stronger, compared to the effects of wetter springs and the three drought types identified in Figures 5 (previously Figure 4). Alongside this new Figure 6, we provide and discuss species-level responses (L215, L393, Figure S10-S12). We also show that the co-occurrence of anomalies within the same season (e.g. warmer and wetter spring conditions) may lead to positive interactions that increase mortality risk even further (L202, Figure S8). Importantly, we clearly note that these results represent local sensitivity analyses and should not be interpreted in a linear manner (L204, L717). Random forest models are inherently non-linear and can produce unreliable responses when extrapolated beyond the range of observed feature combinations. To avoid such artefacts, all predictions were explicitly constrained to the observed feature space, ensuring that estimated changes in mortality risk reflect realistic local perturbations rather than extrapolations into unobserved climatic conditions (L716).

L190: What does "milder lows" mean? What are the temperature ranges for this term? A similar comment for the caption of Figure 3.

The term "milder lows" was indeed ambiguous and had been used inconsistently. It was intended to convey that an increase in a season's minimum temperature anomaly was associated with elevated mortality risk. To resolve this, we removed the term and harmonized the terminology throughout the manuscript (L171ff, Figure 4 [previously Figure 3]). We now clearly distinguish between the maximum anomaly, minimum anomaly, and average deviation, and explicitly link each to its ecological interpretation: the warmest (or wettest) event, the coldest (or driest) event, and average temperature (or water availability) conditions during the years preceding a tree's mortality assessment, respectively. These definitions are introduced and illustrated in the new Figure 1, and the consistent terminology is carried through Figures 4 and 5 (previously Figures 3 and 4) and the results section (L171ff).

As noted above, providing a specific temperature range is not meaningful in this context, since the predictors are site-specific standardized anomalies rather than absolute values.

Comments on the importance of temperature over water availability:

L171-178: This paragraph indicates that warm temperatures are the primary cause of tree mortality, regardless of whether conditions are dry or wet, isn't it?

L281-283: Again, it is possible that either dry or wet is not important, and temperature is the dominant factor for tree mortality.

We agree that temperature is an important driver of tree mortality, as it is now consistently mentioned as first driver in the revised abstract, results, and discussion - with explicit statements highlighting its importance (e.g., L200). However, we disagree with the interpretation that temperature alone explains the observed mortality patterns. Feature importance rankings show that anomalies in the climatic water balance are of comparable importance to temperature anomalies and forest competition across species. In addition, the new analysis of predicted additive mortality risk (new Figure 6) demonstrates that the effects of temperature and climatic water balance anomalies are of similar magnitude, indicating that neither driver can be considered secondary. As argued throughout the first revision, our approach ensures that the feature importance of temperature or climatic water balance anomalies is not inflated due to correlation (L583ff). We further clarify that the interpretation of L187 (previously L171-178) is not that temperature dominates over water availability. Rather, these results show that warming during winter and spring are selected consistently across models, irrespective of whether concurrent climatic water balance anomalies indicate drier or wetter conditions. This lack of seasonal overlap suggests distinct mortality pathways rather than a dominance of temperature over water availability.

We note that the reviewer agrees that warm-wet conditions can promote structural overshoot. By definition, however, structural overshoot implies an increase in canopy size and thus evaporative demand, which inherently increases vulnerability to subsequent drought[5,6] (L419ff). In this context, the suggestion that trees can generally prevent drought impacts through stomatal closure does not hold given well-established evidence on evaporative demand. Larger trees, which are consistently more vulnerable to mortality, have longer hydraulic pathways and greater leaf area and therefore higher evaporative demand[7,8]. This demand is directly driven by atmospheric conditions and cannot be fully offset by stomatal regulation alone: minimum conductance through incomplete stomatal closure and cuticular transpiration results in persistent water loss, which pushes trees closer to hydraulic safety thresholds under drought conditions[9–12]. Similarly, the argument that wet conditions would allow rapid compensatory root development is not given. Empirical evidence shows that canopy expansion in response to favorable conditions occurs relatively quickly (few years), whereas root system development to compensate for the increased water demand generally lags (up to a decade)[13–15]. As a result, increases in evaporative demand following rapid canopy growth under favorable growth conditions cannot be readily compensated by root growth, and thus, structural overshoot causes increased vulnerability to subsequent drought events. We are discussing this mechanism now in more detail (L396, L416). Finally, if water availability were not an important driver, an alternative explanation would be required not only for the extensive literature on drought-induced tree mortality[1,3,11,16–18], but also for the strong and consistent climatic water balance signal observed across species in our analyses, including the emergence of distinct drought types that match established drought-induced mortality frameworks.

378-379: Sapflow data are needed to justify this statement.

We agree that sapflow data would be valuable to further quantify the role of structural overshoot. However, such measurements are beyond the scope of this study, as they would require sapflow observations across the full set of National Forest Inventory sites, encompassing hundreds of thousands of trees. The worldwide most extensive sapflow database SAPFLUXNET (https://sapfluxnet.creaf.cat) contains, for example, only 4 sites in France (and two of them are only a few 100 m apart), showing that countrywide assessments and conclusions are elusive. For this reason, we deliberately use cautious wording when discussing structural overshoot and frame it as a working hypothesis rather than a demonstrated mechanism (L456). As discussed in the Limitations section, this hypothesis should be tested in future studies using in-situ physiological measurements. Where relevant, we therefore rely on existing literature for context rather than direct physiological validation, which is (as stated above) not possible at present and will require massive investment in sapflow sensors to obtain space-representative coverage in the future.

Remaining comments:

L137: "topography" could be a factor determining temperature the precipitation conditions. "NDVI" is also highly related to environmental factors including topography, temperature the precipitation. Is it possible that the autocorrelations between these factors have accounted for the relative minor impacts of these factors?

We acknowledge that topography and NDVI are closely linked to environmental gradients, including temperature and precipitation. However, autocorrelation among features does not bias feature importance estimates in our analyses. Random forests reduce the impact of correlated predictors by randomly sampling both observations and predictor subsets when building individual decision trees. In addition, we explicitly remove highly correlated features prior to the final model fitting to minimize any redundancy (see new Figure 1, L607). We further added that the relatively lower importance of topographic variables is consistent with previous studies that have applied similar modeling approaches to large-scale forest inventory data[19,20] (L153).

L233-235: The authors have addressed number of models in explaining the mortality under different conditions. I wonder whether the calculated mortality rates across the 500,000 trees (L442-443) differ significantly between warm-dry and warm-wet conditions, based on statistical significance tests. If so, I am not sure if the warm-dry and warm-wet based estimates are necessary.

The estimated mortality rates reported across the full inventory (L97ff) quantify observed mortality aggregated across all climatic conditions and are not intended to quantify differences between specific anomaly combinations such as warm-dry versus warm-wet conditions. Our analyses instead focus on model-based associations between mortality risk (i.e., the probability to die within two forest inventory censuses) and seasonal climate anomalies, assessed across many independently trained models. The number and proportion of models identifying a given anomaly combination as mortality-promoting therefore reflect the consistency and robustness of the association across species and sites, rather than differences in raw observed mortality rates across climatic gradients. Nonetheless, as argued above and shown in Figures 6 (new) and 8 (previously 6), anomalies of the climate water balance (irrespective of being drier or wetter) have a distinct fingerprint on tree mortality risk, which is comparable in magnitude as temperature anomalies.

L235-236: The physical implication of this sentence is confusing. If species are "longer drought tolerance", how could wetter (a similar comment: how wet? | more quantitative definitions are needed) conditions are associated with the mortality of the species?

We believe this comment reflects a misunderstanding of both the cited sentence and the definition of climatic anomalies used in this study. The original sentence in question stated:

> *"Consistently, species with lower drought tolerance (temperate species) had significantly more models associating mortality with wetter conditions than drought-tolerant (Mediterranean) species (p = 0.02, Figure 5B). Similar but weaker correlations showed with drought-tolerant species having fewer warmer-wetter models (p = 0.10) and, conversely, having more drier (p = 0.12) and warmer-drier models (p = 0.10)."*

This pattern indicates that wetter-than-usual conditions (see details on using standardized anomalies above) are less likely to promote mortality risk in drought-tolerant species than in temperate species. As explained in the Discussion (L409ff), one possible interpretation (which is supported by an independent remote-sensing study[6]) is that Mediterranean species are adapted to recurrent water limitation and thus less likely to rapidly expand their canopy under favourable growth conditions. However, we also highlight observations of structural overshoot in Mediterranean species[21,22] (L421). In contrast, temperate species have a shorter growing-season and thus may respond more strongly to wetter conditions, increasing canopy growth and vulnerability to subsequent drought stress[23]. To avoid further confusion of the sentence in question, we rewrote it to as follows (L262):

> *"Using a drought-tolerance index[46] as a proxy for climatic origin (see Methods), we found the same pattern: across all models, the percentage of models linking mortality to wetter spring conditions decreased significantly with increasing drought tolerance (p = 0.02; Figure 7B), whereas the percentage of models linking mortality to drier summer conditions increased, although not significantly (p = 0.12). Similarly, within the subset of models linking mortality to warmer anomalies, the model percentage linking mortality to wetter spring conditions decreased with increasing drought tolerance, respectively, the percentage linking mortality to drier summer conditions increased (however both non-significant with a p = 0.10)."*

L240: What is the average height of "taller species". With increased water availability, canopy diameters could expand, not just their heights. What's the justification for the allometry used here?

We clarified that a species' mean height refers to the mean height across all trees of a given species in the inventory (L732). When we refer to "taller species being more vulnerable", we specifically mean that there is a significant positive relationship between species mean height and the percentage of models associating mortality with wetter conditions (Figure 7C, previously Figure 5C). The closest available proxies for canopy size in the National Forest Inventory are tree height and diameter at breast height (DBH). We chose height because it more directly reflects evaporative demand and hydraulic vulnerability, which are well-established to increase with tree height[7,8]. In addition, height allowed us to simultaneously test the hypothesis of intensified light competition (due to canopy expansion during wet springs), for which height is a more direct proxy of canopy position than DBH.

To assess the robustness of our choice, we repeated the species-level analysis relating the percentage of models associating mortality with wetter conditions using species mean DBH instead of height, as well as the tree-level analysis relating sensitivity to wetter and drier conditions to DBH (Table S10, previously Table S9). The results are nearly identical: species with larger average diameters also show a higher proportion of models associating mortality with wetter conditions (see Figure below), and tree-level relationships remain similarly inconclusive whether height or DBH is used (lengthy table omitted here for readability). This shows that our conclusions are not sensitive to the specific allometric proxy chosen.

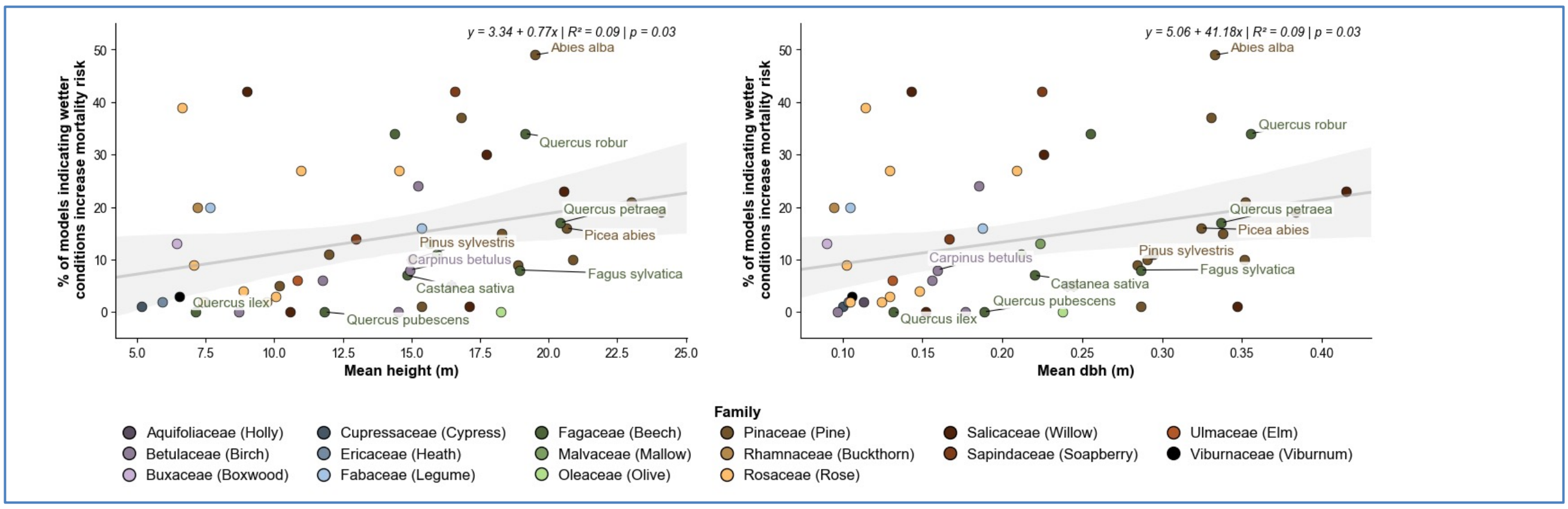

L249-250: A conclusive or and transition sentence is needed. By reading this sentence, I am confused and wonder if this manuscript is trying to solve this issue or this is a suggestion for future research.

We thank the reviewer for pointing out this loose end. The sentence was intended to guide the reader from across- to within-species analysis. We revised the paragraph to make this clearer now (L276):

> *"These results indicate that taller, temperate species were more frequently associated with mortality under anomalously wet spring conditions, possibly due to structural overshoot. The next section examines whether structural overshoot also emerges from tree-level responses within species."*

L392: This sentence is hard to understand.

We agree that the original sentence was difficult to follow and required clearer context. We therefore rewrote this passage to more explicitly explain that reduced spring water deficits relative to site-specific norms may relax growth constraints, potentially encouraging canopy overinvestment. The revised wording clarifies this better, whilst emphasizing that this interpretation is tentative (L426):

> *"Interestingly, wetter springs in our models were characterized by reduced spring water deficits relative to the site norm. This suggests that when spring water scarcity is less severe than what is usually to be expected at a given site, it may fail to provide a certain growth constraint, encouraging trees to over-invest in canopy growth. While this interpretation remains tentative, it supports the notion that relative deviations from local climatic expectations, rather than absolute water availability, shapes tree mortality risk[21,68]."*

Reviewer #3 (Remarks on code availability):

I've checked the links in Data Availability. All the links are pointed me sources of data except for:

"Data on soil water conditions and soil pH is freely available at (https://silvae.agroparistech.fr). C.P. provided the temperature, precipitation, and evapotranspiration data (partially available at https://silvae.agroparistech.fr)." I am not sure if this is because of my VPN and/or IP address. I am leaving this to the editorial office to further explore.

We were informed that there are currently technical issues affecting the hosting of the https://*silvae.agroparistech.fr* website, which is under maintenance. The site is reachable again, but data access is temporarily limited. Nonetheless, all data required to reproduce our analyses are fully available via the Zenodo archive referenced in the Data Availability statement.

References

1. McDowell, N. et al. Mechanisms of plant survival and mortality during drought: why do some plants survive while others succumb to drought? New Phytol. 178, 719–739 (2008).
2. Anderegg, L. D. L., Anderegg, W. R. L. & Berry, J. A. Not all droughts are created equal: translating meteorological drought into woody plant mortality. Tree Physiol. 33, 672–683 (2013).
3. Choat, B. et al. Global convergence in the vulnerability of forests to drought. Nature 491, 752–755 (2012).
4. Vicente-Serrano, S. M., Beguería, S. & López-Moreno, J. I. A Multiscalar Drought Index Sensitive to Global Warming: The Standardized Precipitation Evapotranspiration Index. J. Clim. 23, 1696–1718 (2010).
5. Jump, A. S. et al. Structural overshoot of tree growth with climate variability and the global spectrum of drought-induced forest dieback. Glob. Change Biol. 23, 3742–3757 (2017).
6. Zhang, Y., Keenan, T. F. & Zhou, S. Exacerbated drought impacts on global ecosystems due to structural overshoot. Nat. Ecol. Evol. 5, 1490–1498 (2021).
7. Bennett, A. C., McDowell, N. G., Allen, C. D. & Anderson-Teixeira, K. J. Larger trees suffer most during drought in forests worldwide. Nat. Plants 1, 15139 (2015).
8. McDowell, N. G. & Allen, C. D. Darcy's law predicts widespread forest mortality under climate warming. Nat. Clim. Change 5, 669–672 (2015).
9. Wang, S., Hoch, G., Grun, G. & Kahmen, A. Water loss after stomatal closure: quantifying leaf minimum conductance and minimal water use in nine temperate European tree species during a severe drought. Tree Physiol. 44, tpae027 (2024).
10. Hasanuzzaman, M., Zhou, M. & Shabala, S. How Does Stomatal Density and Residual Transpiration Contribute to Osmotic Stress Tolerance? Plants 12, (2023).
11. Anderegg, W. R. L. et al. Meta-analysis reveals that hydraulic traits explain cross-species patterns of drought-induced tree mortality across the globe. Proc. Natl. Acad. Sci. 113, 5024–5029 (2016).
12. Martin-StPaul, N., Delzon, S. & Cochard, H. Plant resistance to drought depends on timely stomatal closure. Ecol. Lett. 20, 1437–1447 (2017).
13. Bose, A. K. et al. Lessons learned from a long-term irrigation experiment in a dry Scots pine forest: Impacts on traits and functioning. Ecol. Monogr. 92, e1507 (2022).
14. Zweifel, R. et al. Determinants of legacy effects in pine trees – implications from an irrigation-stop experiment. New Phytol. 227, 1081–1096 (2020).
15. Gessler, A. et al. Shaping future forests: How can ecophysiology support climate-smart forest management? New Phytol. https://doi.org/10.1111/nph.71007 (2026) doi:10.1111/nph.71007.
16. Allen, C. D. et al. A global overview of drought and heat-induced tree mortality reveals emerging climate change risks for forests. For. Ecol. Manag. 259, 660–684 (2010).
17. Choat, B. et al. Triggers of tree mortality under drought. Nature 558, 531–539 (2018).
18. Schuldt, B. et al. A first assessment of the impact of the extreme 2018 summer drought on Central European forests. Basic Appl. Ecol. 45, 86–103 (2020).
19. Taccoen, A. et al. Background mortality drivers of European tree species: climate change matters. Proc. R. Soc. B Biol. Sci. 286, 20190386 (2019).
20. Etzold, S. et al. One Century of Forest Monitoring Data in Switzerland Reveals Species- and Site-Specific Trends of Climate-Induced Tree Mortality. Front. Plant Sci. 10, 307 (2019).
21. Italiano, S. S. P. et al. Drought legacies in mixed Mediterranean forests: Analysing the effects of structural overshoot, functional traits and site factors. Sci. Total Environ. 927, 172166 (2024).
22. Paz, D. B., Rey, A., Zavala, M. Á. & Araújo, M. B. Recent climate change reduced Spanish forests' carbon sink capacity. https://eartharxiv.org/repository/view/10795/ (2025).
23. Zavala, M. A. Excess plant growth worsens droughts. Nat. Ecol. Evol. 5, 1474–1475 (2021).

Review Comments

The authors present interesting findings regarding tree mortality patterns and the potential role of structural overshoot. The analysis is difficult to understand due to methodological ambiguities and explanatory gaps. Some of the data processing and analytical approaches are inadequately described, and lacking connections between different analytical steps, making the study difficult to comprehend and evaluate fully. Overall, I recommend the manuscript for major revision before it can be properly evaluated and make a substantive contribution to the field.

Specific comments:

1. Lines 25-27: You emphasize "background mortality" in the introduction, but Lines 395-396 reveal an ambiguity by including "sites with irregular mortality events" in your analysis. Without clear differentiation between background and catastrophic mortality, potential drivers could be confounded. Please either clarify how you operationally distinguished between these mortality types or explicitly acknowledge their combined analysis.
2. Lines 73-76: The 1.5-fold to 4.0-fold mortality rate increases are striking but lack ecological context. These shocking numbers need careful explanation. Are these increases balanced by changes in productivity and recruitment? Readers could misunderstand this if these patterns represent forest degradation or simply shifts in forest dynamics.
3. L109: Before presenting the SHAP analysis, please report the overall performance (R^2) of the Random Forest models and quantify what percentage of mortality variation cannot be explained by your selected drivers.
4. The method for SHAP analysis is inadequately explained. For example, in Figure 2A, you present a single "temperature anomaly" variable, but your methods indicate you used multiple temperature variables. How did you merge these different variables? Similarly, in Figure 2B, how did you handle correlations between temperature variables?
5. You state, "For Fagus sylvatica, 72% of the models associated mortality with drier conditions and 28% with wetter" which conflicts with "84% of the models associated mortality with drier conditions and 16% with wetter conditions." Please clarify this discrepancy.
6. Figure 5 is difficult to understand without proper explanation. Define "vulnerability" before using it, and clarify how the scaled SHAP values calculated, and could show figures to explain it shoulbe better, and how they relate to those in Figures S7/S8.
7. Figures S16 and S17 are not mentioned in the main text. Please ensure all supplementary materials are properly referenced and support the main text.
8. Lines 446-448: For the SMOTE, please specify the k-value used for the nearest neighbor algorithm and address how uncertainty due to choice of k value in synthetic sampling affects model reliability.

9. While you correctly applied SMOTE only to training data to prevent leakage, your validation approach needs strengthening. You must verify synthetic samples represent realistic mortality scenarios, not just statistical constructs. Include clear cross-validation procedures, proper metrics for imbalanced data (precision, recall, F1-score, AUPRC), and analyze how synthetic sampling uncertainty affects ecological interpretation.
10. Lines 462-464: The decision to keep exactly two features per category during feature selection seems arbitrary. Was this empirically determined? Did you test whether some categories might deserve more features than others? What are these features, temperature itself has many variables for different seasons. Unclear enough and so hard to understand it well.
11. Lines 425-429: For SPEI calculation, you mention testing timescales from 1-24 months but never specify which ones were most informative. The choice of evapotranspiration formula can significantly affect drought index values - which one did you use?
12. Lines 422-429: The climate data description seems lack crucial details about spatial resolution, data sources, and how you matched climate data to individual tree locations. For sites with complex terrain, was any microclimate adjustment performed?
13. Lines 487-492: Your SHAP analysis lacks technical specifics. Which SHAP algorithm variant did you use? How did you aggregate SHAP values across the ensemble of trees?
14. Lines 506-508: The 60% threshold for consistent responses across models seems arbitrary. A sensitivity analysis would strengthen confidence in your classification approach.
15. The structural overshoot hypothesis, central to your paper, is supported only by indirect evidence. Consider using available methods to directly quantify structural overshoot to strengthen your conclusions. There are literatures and data (in situ data or remote sensing prodcuts) to support your analysis.
16. Lines 476-478: You mention comparing random forests with logistic regression models but provide no details or results from this comparison.
17. Lines 519-521: For species trait correlations, did you consider phylogenetic non-independence? Closely related species often share trait values due to evolutionary history rather than independent adaptation.
18. Lines 404-405: Your assumption that harvested trees were "survivors" is significant. How many trees fell into this category? In managed stands, this could seriously skew your mortality estimates, especially if salvage logging practices varied by region or species.
19. Lines 383-389: The NFI sampling methodology is explained, but how the shifting grid pattern ensures complete coverage remains unclear. A simple diagram in the supplementary materials would help clarify this.

20. More discussion of potential sampling biases in the NFI dataset would be helpful. Were there changes in protocol during your study period? Were all forest types equally accessible for sampling? These factors could affect your interpretation of spatial and temporal trends.
21. Please clarify how you handle potential disturbances like fire that might affect mortality but aren't explicitly addressed in your analysis.
22. Line 480: You set ROC-AUC > 0.6 as your threshold for model retention but don't report how many models failed this test. This information helps assess whether certain species or conditions were particularly difficult to model.
23. Lines 411-421: Your feature categories are spread across several paragraphs with embedded references. A simple table listing each category with example features would improve clarity.
24. Do you include all SPEIs with different month scales in the machine learning model? How do you address the high correlations between them? Similar to the different temperature variables or other variables.
25. Lines 430-434: The seven-year window for "short-term" climate analysis seems arbitrary. Is there ecological reasoning behind this choice? How do you account for lag effects in tree responses that vary by species and climate zone?
26. Your title emphasizes spring conditions ("Flush to Crush"), but the discussion section (Lines 270-288) focuses extensively on winter conditions affecting mortality with comparatively less attention to spring effects. This creates a disconnect between your title's emphasis and your discussion. Please provide more balanced analysis of spring conditions to better align with your title.
27. For Figure 6, you analyze vulnerability to wetter conditions and discuss this around L336-337: "Secondly, wetter springs disproportionately affected taller species (Figure 6C), suggesting that structural overshoot poses a greater challenge for canopy-forming trees." How about the analysis for vulnerability to drier conditions. Does the relationship between tree height and vulnerability to drier conditions also support your structural overshoot hypothesis?
